# Using Phylogeny and a Conserved Genomic Neighborhood Analysis to Extract and Visualize Gene Sets Involved in Target Gene Function: The Case of [NiFe]-hydrogenase and Succinate Dehydrogenase

**DOI:** 10.1264/jsme2.ME25018

**Published:** 2025-11-13

**Authors:** Tomoyuki Kosaka, Minenosuke Matsutani

**Affiliations:** 1 Research Center for Thermotolerant Microbial Resources, Yamaguchi University, Yamaguchi 753–8515, Japan; 2 Graduate School of Science and Technology for Innovation, Yamaguchi University, Yamaguchi 753–8515, Japan; 3 Department of Food, Aroma and Cosmetic Chemistry, Faculty of Bioindustry, Tokyo University of Agriculture, Hokkaido 099–2493, Japan

**Keywords:** genome information, protein maturation, gene cluster, molecular phylogeny, gene neighborhoods

## Abstract

Several enzymes have subunits that require the addition of cofactors or maturation of the active center, which is facilitated by other genes. Information on these functionally-related genes not only aids in the functional anal­ysis of target genes, but is also useful for heterologous expression. In the present study, we analyzed the homologs of a target gene and their relationships with adjacent genes within the genome by constructing clusters of neighboring genes, quantifying the number of clustered genes, and examining their conservation in a taxonomic clade of target gene homologs. [NiFe]-hydrogenase was selected as the target because of the availability of a concrete database for subsequent evaluations in our anal­ysis. The present results indicate that genes associated with target gene function were conserved according to the molecular phylogeny of the target gene. We subsequently introduced automated clustering of the phylogenetic tree clade of clustered genes and applied this method to large datasets not yet analyzed and our previous data. The results obtained suggest that this approach provides insights into a comprehensive set of genes involved in cellular functions, particularly when the genes being analyzed are complex and require maturation. The procedure developed herein also provided similar and reproducible results on previously analyzed succinate dehydrogenase, which was not arbitrary.

Despite substantial advances in genome sequencing technology, which have facilitated the acquisition of genetic information on microorganisms (Shendure *et al.*, 2017; Uhlen and Quake, 2023), the real cellular functions of genes remain largely unknown, and novel methods for elucidating gene function are highly expected. The functions of numerous genes may be predicted from their encoded amino acid sequences using several bioinformatics techniques, such as BLAST+ (Camacho *et al.*, 2009) and HMMER tools (Eddy, 2011), in combination with highly informative databases, including UniProtKB (UniProt Consortium, 2025), eggNOG (Powell *et al.*, 2012), Pfam (Paysan-Lafosse *et al.*, 2025), InterPro (Blum *et al.*, 2025), dbCAN (Zheng *et al.*, 2023), KEGG (Kanehisa and Goto, 2000), TIGRFAMs (Haft *et al.*,‍ ‍2001), and CDD (Wang *et al.*, 2023). However, empirical validation and biological insights are essential for elucidating and confirming their actual cellular functions, particularly in uncultivable microorganisms and those lacking developed genetic techniques. A common strategy employed to investigate gene function involves functionalization using a cellular platform, such as the expression of heterologous genes in model microorganisms. While this approach is considered to be effective, its implementation involves numerous technical and conceptual challenges. The integration of bioinformatics-based computational methods may not only assist in predicting gene function, it also facilitates the rational selection of gene sets and host organisms for co-expression and provides crucial guidance for experimental design and interpretation in biochemical research.

Gene function in heterologous cells sometimes requires the co-expression of other genes, which may be classified into structural subunits, maturation factors, and proteins involved in cofactor biosynthesis (Thöny-Meyer *et al.*, 1995; Posewitz *et al.*, 2004; Sun *et al.*, 2010; Caetano *et al.*, 2011; Shiota and Kosaka, 2025). Structural subunits are directly associated with the cognate protein encoded by other genes, and these function together for protein maturation. Maturation factors, including chaperones and endopeptidases, are involved in post-translational target protein modifications and the formation of bonds with small molecules, such as prosthetic groups. Other proteins functionally related to target protein maturation using small molecules for prosthetic groups, flavins, and hemes, or small molecule and metal ion transporters require access to the catalytic reaction center. Therefore, these supporting genes encoding small molecule proteins need to be co-expressed with the target gene whose function is being exami­ned. Several enzymes require such genes for heterologous expression (in *Escherichia coli*), such as succinate dehydrogenase (SDH) (protein required: SdhE) (McNeil *et al.*, 2012), cytochrome *c* (the ccm system) (Inoue *et al.*, 2011), [NiFe]-hydrogenase (maturation factors) (Maier *et al.*, 2015), and [FeFe]-hydrogenase (factor required: HydEFG) (Kuchenreuther *et al.*, 2010). In bacteria and archaea, gene clusters related to the function of specific genes, such as subunits and maturation factors, have an operon structure (Lawrence and Roth, 1996; Fang *et al.*, 2008; Fondi *et al.*, 2024). Therefore, these genes may be located near the target gene as gene neighborhoods. An anal­ysis of these gene neighborhoods provides a rational strategy for identifying candidate genes required for target protein function, particularly in cases where experimental information is limited.

Computational methods for visualizing gene neighborhoods associated with gene function from genomic data have been a significant focus within bioinformatics. Analyses based on conserved gene neighborhoods (CGNs) have predominantly focused on elucidating protein-protein interactions (Robin *et al.*, 2022), and various prediction programs have been developed for this purpose (Anjos *et al.*, 2021). Additionally, CGN-based algorithms have been implemented in function prediction servers (Törönen and Holm, 2022) and in the prediction of protein homo- and hetero-dimers (Esch and Merkl, 2020). CGNs have also been applied to the identification of genes associated with similar metabolic functions (Zaharia *et al.*, 2019). The three key steps in a computational study on protein function currently include anal­yses of i) the relationships between homologous proteins, ii) the protein domain architecture, and iii) the CGNs of the target protein (Gumerov and Zhulin, 2020). Furthermore, methods have been developed to handle interconnected gene neighborhoods in bacterial and archaeal genomes (Rogozin *et al.*, 2002), and lists of CGNs have been used to categorize genes into paralogous and orthologous clusters (Fouts *et al.*, 2012). Based on these assumptions, databases integrating genes and related information, particularly the STRING database, which collects information on these interactions, have been constructed and are evaluated, scored, and subsequently transferred to less well-studied organisms using hierarchical orthology information (Szklarczyk *et al.*, 2023). While these resources are useful for visualizing potential gene interaction networks, they typically do not focus on identifying specific functionally-related genes required for protein maturation or expression. Although several methods exist for visualizing gene neighborhoods, they are often limited to showing the positional relationship between a specific gene and its neighboring genes within a single genome (Gumerov and Zhulin, 2020; Price and Arkin, 2024). Therefore, systematic procedures for comparing conserved functionally-related genes across genomes have not yet been thoroughly exami­ned.

Several concepts have been introduced to predict these supporting genes using CGNs. Genes required for the maturation of certain functional gene products are present in the genome, and proteins with a high degree of sequence similarity are expected to share similar maturation-related genes due to their molecular phylogeny. We previously reported the relationship between the molecular phylogeny of the gene encoding the flavoprotein subunit of SDH of *Pelotomaculum thermopropionicum* and CGNs (Kosaka *et al.*, 2023). In this study, we investigated the extraction and visualization of functionally and spatially related genes from genomic information based on [NiFe]-hydrogenase, a gene that has been extensively exami­ned. [NiFe]-hydrogenase is predicted to contribute to succinate oxidation by SDH in *P.‍ ‍thermopropionicum* (Kosaka *et al.*, 2023). [NiFe]-hydrogenase comprises four subunits (Beaton *et al.*, 2018) and requires maturation factors for its activation (Lacasse and Zamble, 2016). Therefore, it includes subunits, maturation factors, and other functionally-related proteins. Moreover, hydrogenases have well-documented information on gene classification and phylogeny, motifs for metal centers, and genetic organization (Greening *et al.*, 2016) as well as a database of aggregated advanced classification and anal­yses (Søndergaard *et al.*, 2016). Therefore, [NiFe]-hydrogenase represents a good target model for evaluating the effectiveness of the analytical method under consideration. In the present study, functionally-related genes to the target gene encoding the [NiFe]-hydrogenase large subunit were clarified using a phylogenetic classification of the target gene, the gathering of gene neighborhoods from genome sequences, a functional classification of the gene neighborhoods gathered from genomes, and a comparison of their conservation across genomes. The automation of this analytical workflow was then investigated to apply the same procedure to multiple functionally-related genes of the target, allowing us to visualize potential relationships among them. The applicability of the developed procedure to another enzyme, SDH, which we previously exami­ned, was also demonstrated.

## Materials and Methods

### Sequence datasets

To avoid redundant comparisons and reduce computational costs, we selected one representative genome per genus, ensuring both taxonomic balance and phylogenetic diversity. This yielded 1,969 genome information entries (Table S1) retrieved from the‍ ‍NCBI Reference Sequence FTP site (ftp.ncbi.nlm.nih.gov/genomes/refseq/). The genomes were selected to minimize taxonomic bias as much as possible. The genome database selected was used in a previous study (Kosaka *et al.*, 2023).

### Constructed cluster list and ID assignment

**Fig. 1.**
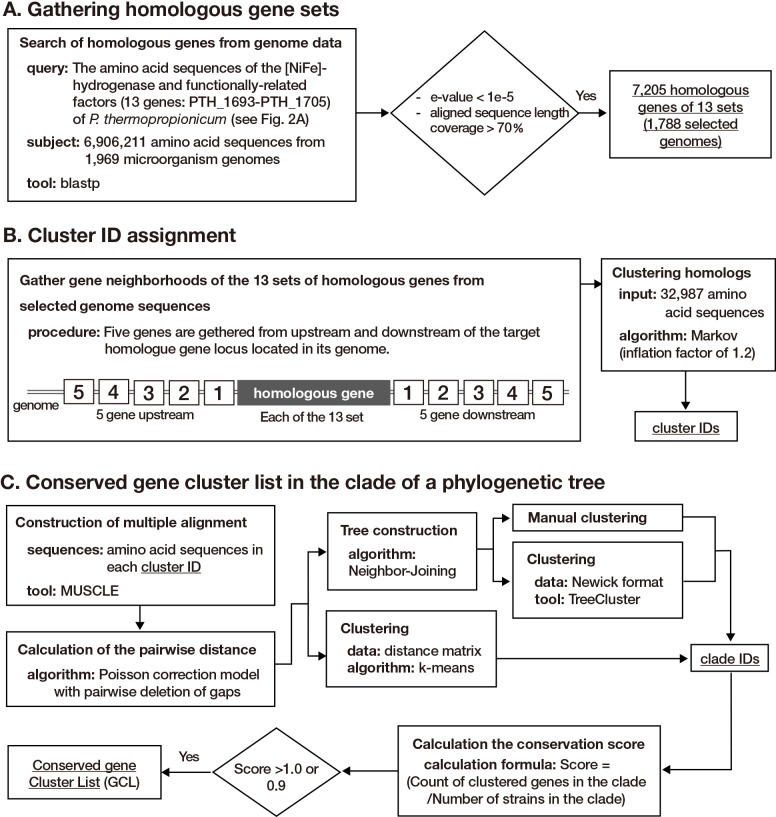
Procedure flowcharts for homologous gene set and conserved cluster ID list construction. A) Gathering homologous gene sets. B) Gathering gene neighborhoods and cluster ID assignment. C) Conserved gene cluster list in the clade of the phylogenetic tree.

The procedures for homologous gene set gathering and cluster ID assignment are shown in Fig. 1A and B. The homologous target gene set was constructed using BLASTp (Camacho *et al.*, 2009) against all protein-coding sequences from the selected genome database based on the criteria of an E-value cut-off of 1e–5 and a minimum aligned sequence length coverage of 70%. In the present study, we used the amino acid sequences of [NiFe]-hydrogenase and functionally-related factors (13 genes: PTH_1693–PTH_1705) of *P. thermopropionicum* as the query sequence because of its relevance for maturation and potential heterologous expression, which aligns well with the purpose of this anal­ysis. To examine genes that are functionally related to the target gene, we applied the reported procedure (Kosaka *et al.*, 2023) with some modifications. Briefly, 10 genes encoded in the region surrounding each hit (five genes each in the upstream and downstream regions) were collected. Therefore, each hit, along with the 10 surrounding genes, was defined as a gene neighborhood and used in the clustering anal­ysis. These candidate-encoded proteins were constructed by comparing the all-against-all protein sequences of 7,205 hits and their surrounding proteins using BLASTp with the same cut-off criteria for homologous gene set construction, followed by Markov clustering with an inflation factor of 1.2 (van Dongen and Abreu-Goodger, 2012). The clusters were then listed and assigned identical numbers in order of the number of genes in the cluster as cluster IDs.

### Phylogenetic conservation of constructed clusters

The procedure for constructing the conserved cluster ID list, involving the phylogenetic tree clade of target genes, is shown in Fig. 1C. Multiple sequence alignments (MSA) of the clustered sequences were constructed using MUSCLE at the amino acid sequence level and were then used for phylogenetic construction (Edgar, 2004a, 2004b). The MEGA X package was employed to generate a phylogenetic tree in order to examine phylogenetic relationships using the neighbor-joining (NJ) approach (Tamura *et al.*, 2007; Kumar *et al.*, 2018). A K-means clustering anal­ysis of the pairwise distance matrix, from the MSA computed using the Poisson correction model in MEGA X (Kumar *et al.*, 2018) with the pairwise deletion of the gap treatment, was performed using the Clustering package in Julia language. Manual clade assignment was initially performed to analyze the constructed phylogenetic tree. Computational clade assignment was performed using TreeCluster (Balaban *et al.*, 2019), which used the output of the Newick tree format from the constructed phylogenetic trees. These constructed clades were used to assemble a conserved gene cluster list (GCL) by counting the number of cluster IDs and the strains in each clade. The criteria score was then calculated by dividing the count of the cluster genes in the clade by the number of strains in the clade. The cluster IDs listed with criteria scores >1.0 indicated conserved clusters in each clade of the tree. The phylogenetic tree was colored using Ruby and R scripts with the ggtree package (Yu *et al.*, 2017). Scripts related to conserved GCL in the clade of a phylogenetic tree are provided at the following repository (https://github.com/tkosaka1976/ME25018-supplemental_files). Hydrogenase class, taxonomy, and Pfam ID were assigned using a taxonomy database (https://www.ncbi.nlm.nih.gov/taxonomy), HydDB (Søndergaard *et al.*, 2016), and HMMER3 hmmscan with the Pfam database (accessed via https://www.uniprot.org), respectively. In Pfam domain searches, hmmscan was run with an E-value cut-off of 0.001.

### Evaluation of clustering using HydDB

To evaluate the results of the classification of the hydrogenase large subunit within each homologous sequence cluster, we used the HydDB dataset (http://www.greeninglab.com/teaching/). HydDB has already classified the hydrogenase large subunit into 8‍ ‍major and 38‍ ‍minor classes using an advanced anal­ysis (Søndergaard *et al.*, 2016). After clustering from the MSA and phylogenetic tree clade, two indices were used in the anal­ysis: the degree to which hydrogenase large subunit sequences (classified into classes in HydDB) were clustered by the anal­ysis (h_class2cluster) and which HydDB class was contained in the cluster divided by the anal­ysis (cluster2h_class). Since each cluster always contains at least one class and each cluster always classifies one, 1 was subtracted from the number of classified classes and used as the count. For example, Fig. 3 shows that Clade A involved [NiFe] Groups 1d, 1e, and 1f; therefore, the count was 3. On the other hand, since Clade B involved only [NiFe] Group 1b, the count was 1. Therefore, the count was always ≥1. If clustering is performed well, the count will be close to 1. To normalize the count by the number of clusters classified, we defined the ratio as the total count divided by the number of clusters.

## Results and Discussion

### Constructed clusters of genes located near the [NiFe]-hydrogenase large subunit gene

**Fig. 2.**
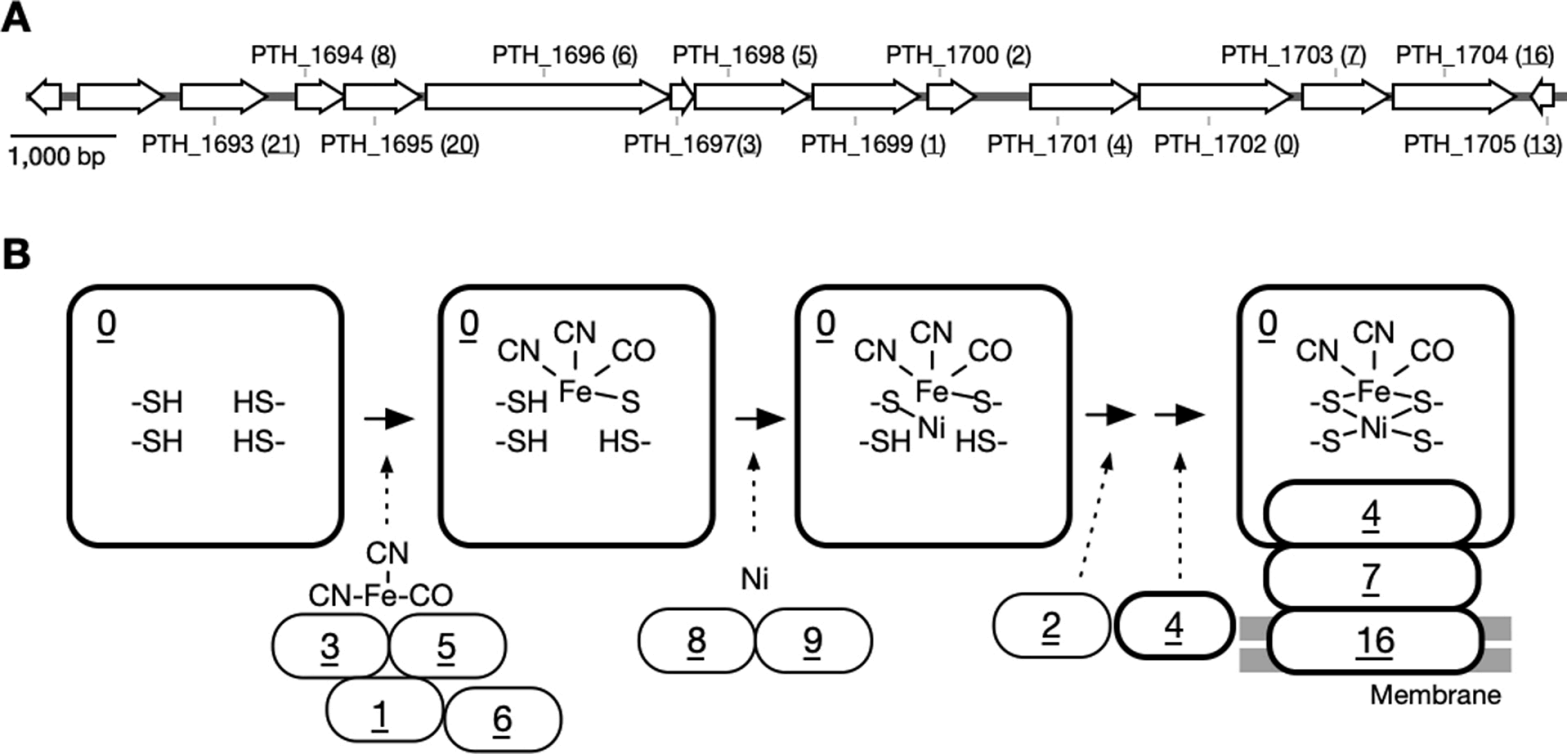
[NiFe]-hydrogenase maturation and related gene clusters. A) Location of the 13 genes encoding [NiFe]-hydrogenase and its candidate maturation factors of *Pelotomaculum thermopropionicum* (HYD4) positioned from 1,781,258 to 1,795,720 of the genome sequence (Accession No.: AP009389.1). The attached locus tags and accompanying cluster ID in parentheses are listed in Table 1. B) Putative maturation mechanisms of the [NiFe]-hydrogenase large subunit and associated gene clusters based on the presumed mechanisms of the [NiFe]-hydrogenase of *E. coli* (Hyd-2) (Soboh *et al.*, 2022). Underlined numbers indicate the gene cluster ID listed in Table 1, and the area enclosed by the bold line and the normal line in the diagram indicates the subunit and maturation factor, respectively.

We performed a gene cluster anal­ysis within a microorganism genome database on the 13 genes (PTH_1693–PTH_1705 from the propionate-oxidizing bacterium *P. thermopropionicum*; Fig. 2A) encoding [NiFe]-hydrogenase and its candidate maturation factors. A total of 7,205 homologous gene sets and 32,986 genes were compiled (Fig. 1A and B), and 7,879 cluster IDs were then assigned to the gene neighborhoods, as shown in Fig. 1B. The list of cluster IDs (Table 1 and S2) shows the clusters of genes predicted to be associated with the target genes. The arrangement of clusters containing [NiFe]-hydrogenase subunit genes was as follows: large subunit (0: cluster ID)>small subunit (4)>Fe–S cluster subunit (7)>membrane subunit (16), representing the complex structural sequence of these subunits from the large subunit (Fig. 2B). In contrast, the orders of clusters, including factors, indicate their importance to the large subunit. The order of factor-including clusters 1, 2, 3, 5, 6, 8, and 9 suggests that the maturation of the [NiFe]-hydrogenase large subunit was affected by Fe(CN)_2_CO insertion, C-terminal extension cleavage, and Ni incorporation. Upon Ni incorporation, the number of genes in clusters 8 and 9 was approximately half that in cluster 0, suggesting a low requirement for the maturation of these genes or a common feature between different hydrogenases in the same genome. Although the gene size of cluster 1 (aminoimidazole ribonucleotide [AIR] synthase related protein: PF00586) was larger than those of clusters 3, 5, and 6, they function together to insert the Fe(CN)_2_(CO) moiety into the [NiFe]-hydrogenase large subunit (Fig. 2B). Cluster 1 plays an active role in Fe and CN–CN–CO insertion into the large subunit, particularly CN, suggesting its higher functional importance in the gene than clusters 3, 5, and 6, which are responsible for similar reactions. The Fe(CN)_2_(CO) moiety may require the turnover of two CN groups for an additional reaction with cluster 1 functioning as a dimer (Miki *et al.*, 2020). In addition, the number of genes in cluster 1 was similar to that in cluster 0, indicating the essential requirement of cluster 1 in the maturation of the large subunit (cluster 0) (Table 1). This significance, based on the gene number, was also observed in cluster 2, which is involved in the cleavage of the C-terminal extension. Higher cluster IDs include subunits (10, 12, and 16) and transcriptional regulators and sensors (11, 17, 18, and 22), but do not include factors (Table 1). These results suggest that the positional proximity of the genes analyzed in the microorganism genome was in the order of functionalization>structure>regulation.

Although genes in each cluster may be compared among individual microorganisms, difficulties are associated with analyzing and understanding them comprehensively on a genome-wide basis. As shown in the list of homologs of *P. thermopropionicum* and *E. coli* in these clusters (Table 1), [NiFe]-hydrogenase as a whole and in individual microorganisms may be compared and understood by moving back and forth between the genetic information obtained on related gene clusters and individual microorganisms. For example, clusters 11 and 12 had high gene numbers, but no homologs in *P. thermopropionicum* or *E. coli* (Table 1). In addition, some clusters were conserved only in *P. thermopropionicum* and *E. coli*, such as clusters 9 and 13. However, the taxonomic classification of microorganisms within each cluster revealed inconsistencies when compared with the whole cluster composition (Table 1 and S2). For example, in clusters 9–13, *Bacillota* were almost completely absent in cluster 12, whereas *Pseudomonadota* were uniformly present in these clusters (Table S2). Therefore, there appear to be phylogenetic differences among the microorganisms in each gene cluster, whereas identifying them in the list is difficult, and detecting relationships among the clusters is also challenging because the presence or absence of genes in the clusters differs for each microorganism.

### Conserved gene cluster distribution in the molecular phylogeny of [NiFe]-hydrogenase large subunit homologs

**Fig. 3.**
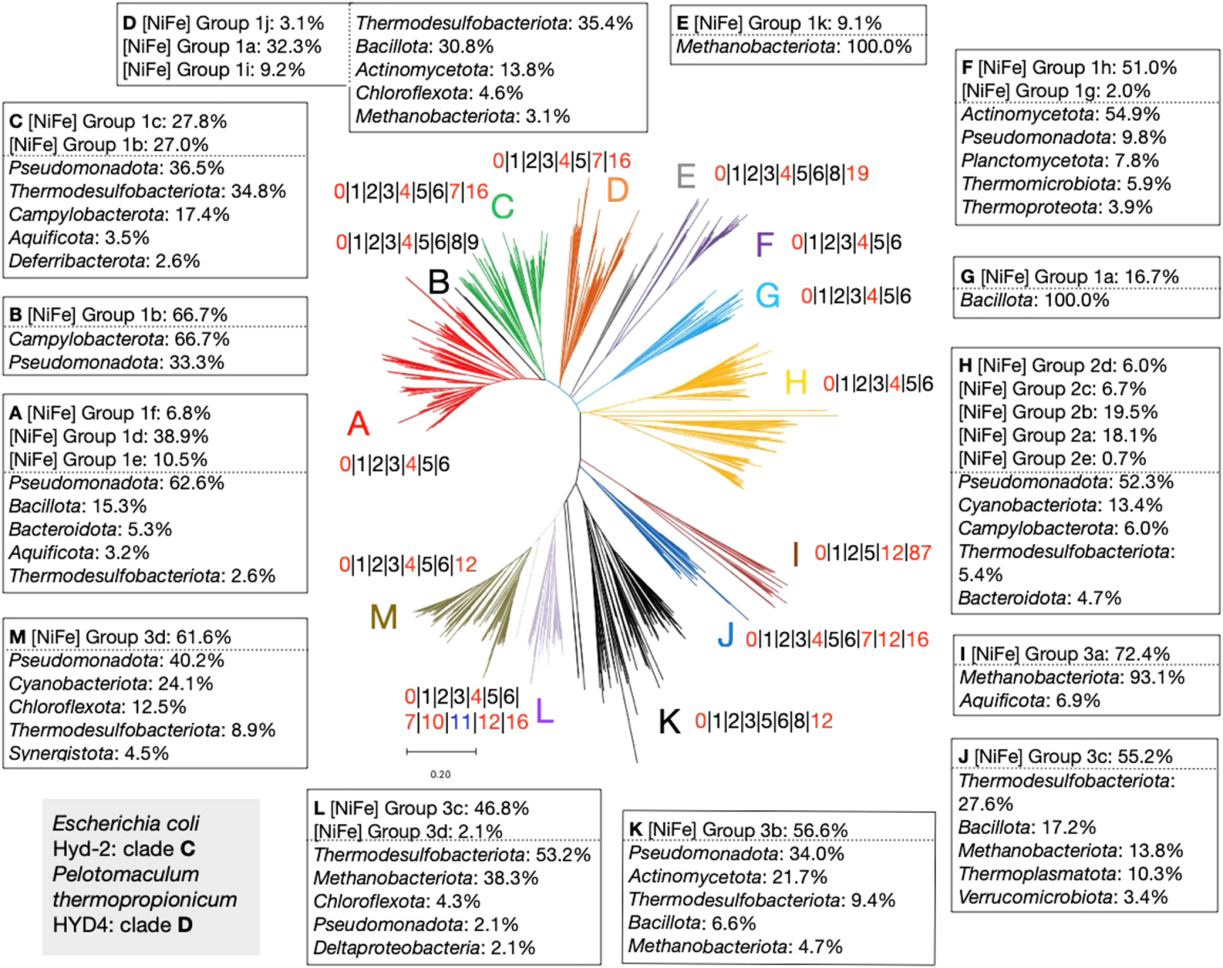
Conserved gene cluster ID list distribution based on the [NiFe]-hydrogenase large subunit (cluster 0) phylogenetic tree. MEGA, including MUSCLE (Edgar, 2004a), is used to create a multiple sequence alignment and the tree of cluster 0 based on 951 amino acid sequences, which includes homologs of the [NiFe]-hydrogenase large subunit. Refer to Fig. 1 for a description of these methods. The numbers, which are cluster IDs from Table 1, separated by “|”, are attached to each clade shown by a letter. These indicate the conserved gene cluster IDs within each clade. The square boxes indicate the percentage of HydDB (Søndergaard *et al.*, 2016) classes assigned in each clade in the top row and the phylum classification of microorganisms with the gene and its percentage in the bottom row.

To analyze the conservation of gene clusters related to the molecular phylogeny of the target gene, a phylogenetic tree of the amino acid sequences of homologous gene sets of cluster 0, such as the [NiFe]-hydrogenase large subunit, was constructed. MSA was performed using MUSCLE, and a tree was constructed using the NJ method. The leaves of the‍ ‍cluster 0 tree were manually classified into 13 clades, clades A–M, and the conserved GCL was assigned (Fig. 3).‍ ‍Comparisons of our classification with HydDB (Søndergaard *et al.*, 2016) revealed that our homologous gene set of cluster 0 corresponded to the [NiFe] groups 1–3 of HydDB, with the separation aligning well with the HydDB classification (Fig. 3). However, clade H was not separated into [NiFe] Group 2 subclass 2a–2e (Fig. 3). Moreover, the gene clusters obtained in each clade revealed the correspondence of the class separation of hydrogenase, whereas the microbial taxonomic classification of genomes encoding genes was not necessarily clustered together, but rather scattered across several clades (Fig. 3). Multiple homologs may be encoded in the same genome and have evolved to perform different functions. These results suggest that the taxonomies of functional proteins and microorganisms need to be analyzed separately.

Our procedure for constructing the GCL is shown in Fig. 1C. Briefly, cluster IDs were plotted if the count of genes assigned to a classified clade in the tree was higher than the count of strains assigned to the clade, namely, a cut-off value >1.0 (Fig. 3). Observations of the GCL in the whole clades of the constructed phylogenetic tree of [NiFe]-hydrogenase large subunit homologs revealed that the clusters for factors conserved in this tree were clusters 1, 2, 3, 5, 6, 8, and 9, which have already been reported as maturation factors for the [NiFe]-hydrogenase large subunit, cluster 0 (Lacasse and Zamble, 2016). Other conserved clusters were clusters 4, 7, 10, 12, 16, and 87, which were subunits that function together with cluster 0, except for cluster 11, a signal sensor protein. These conserved clusters were partly different in each clade, and conservation patterns correlated with the phylogenetic distribution of cluster 0. For example, cluster 12 was conserved in clades I–M, but not in clades A–H (Fig. 3). This result indicates that the functional interaction of the cluster 12 subunit with cluster 0 was from the specific amino acid sequence pattern, and phylogenetically classified amino acid sequences were functionally conserved. This clade classification and cluster conservation were identical to the classification of HydDB, namely, [NiFe] group 3 of HydDB interacted with cluster 12 (Fig. 3). The conservation of clusters 8 and 9, which are required for Ni acquisition in [NiFe]-hydrogenase large subunit maturation, was very low, specifically in clades B, E, and K, which showed only cluster 8 conservation. These results suggest the following: i) some [NiFe]-hydrogenase large subunits do not require maturation factors for Ni acquisition; ii) clusters 8 and 9 may compensate for each other for this function; and iii) the function dependent on each cluster has already been incorporated into some clusters. Among the conserved clusters within the tree of cluster 0, the functional redundancy of clusters 8 and 9 was not consistently observed in this set, indicating a low possibility. Therefore, functional compensation by other clusters is highly likely.

We also analyzed the proteins encoded by the genomes of specific microorganisms, *P. thermopropionicum* HyaB and *E. coli* HybC. HyaB of *P. thermopropionicum* was located in clade D, while HybC of *E. coli* was located in clade C (Fig. 3). These proteins were clearly separated into different clades; however, the GCL patterns in these clades were similar to 0|1|2|3|4|5|7|16, except for cluster 6, which was only present in clade C (Fig. 3). These results suggest that *P. thermopropionicum* HyaB shares a nearly identical set of related gene clusters with *E. coli* HybC and that the heterologous expression and maturation of *P. thermopropionicum* HyaB in host *E. coli* cells may be accomplished by the presence of similar maturation factors.

### Comparison of phylogenetic tree clade clustering methods for automation

**Fig. 4.**
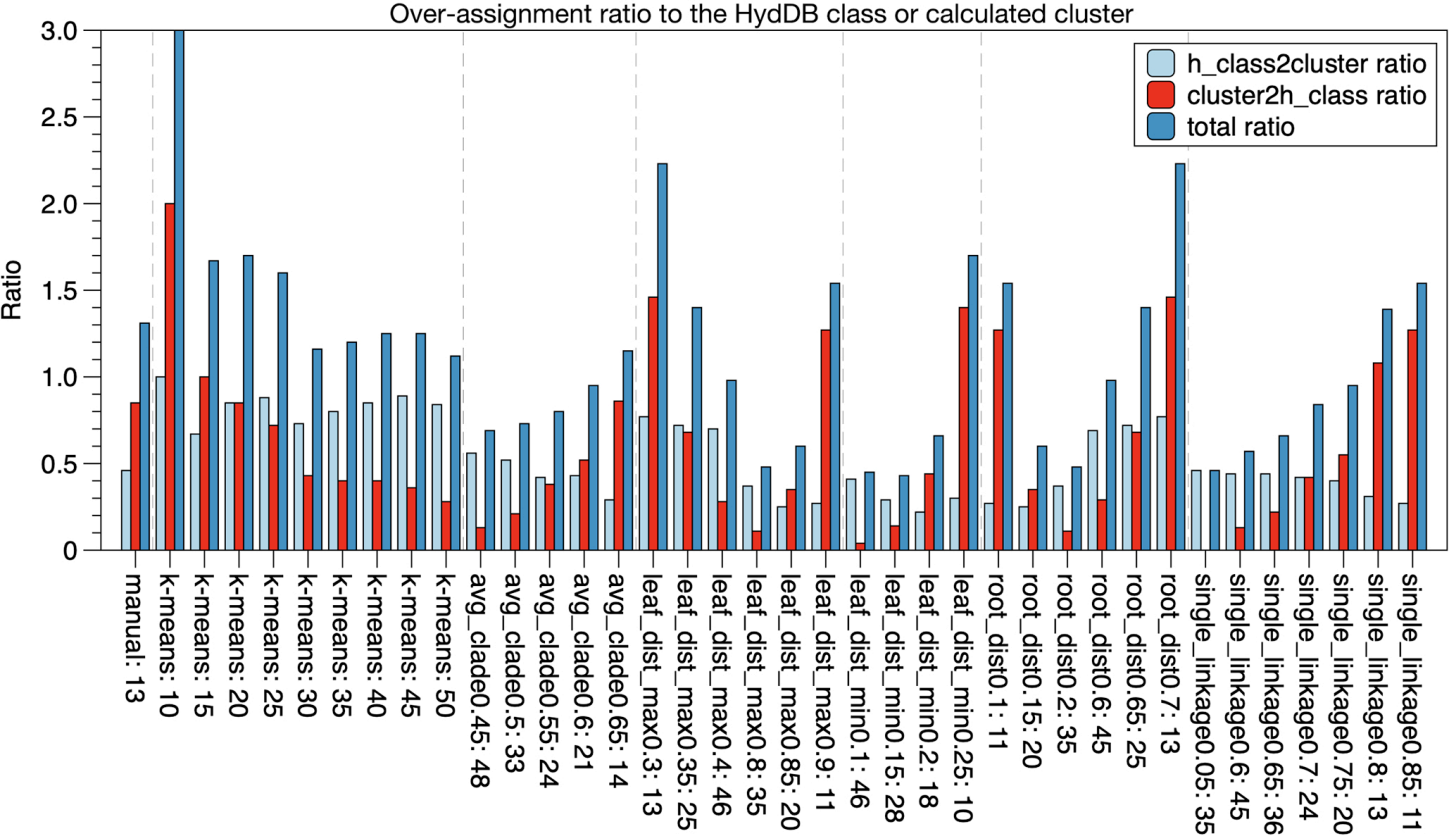
Evaluation of clustering methods for phylogenetic clade classification using HydDB. Cluster 0 homologs are clustered using k-means clustering and TreeCluster (Balaban *et al.*, 2019) using the ave_clade, leaf_dis_max, leaf_dist_min, root_dist, and single_linkage algorithms. h_class2cluster assigns a HydDB (Søndergaard *et al.*, 2016) class to the cluster created by each algorithm, and cluster2h_class assigns a cluster to a HydDB class. The number of classes classified is 1, since one is generally expected for each to be classified and then summed. The counts are added and divided by the number of clusters to obtain a ratio. Total refers to the sum of both values.

**Fig. 5.**
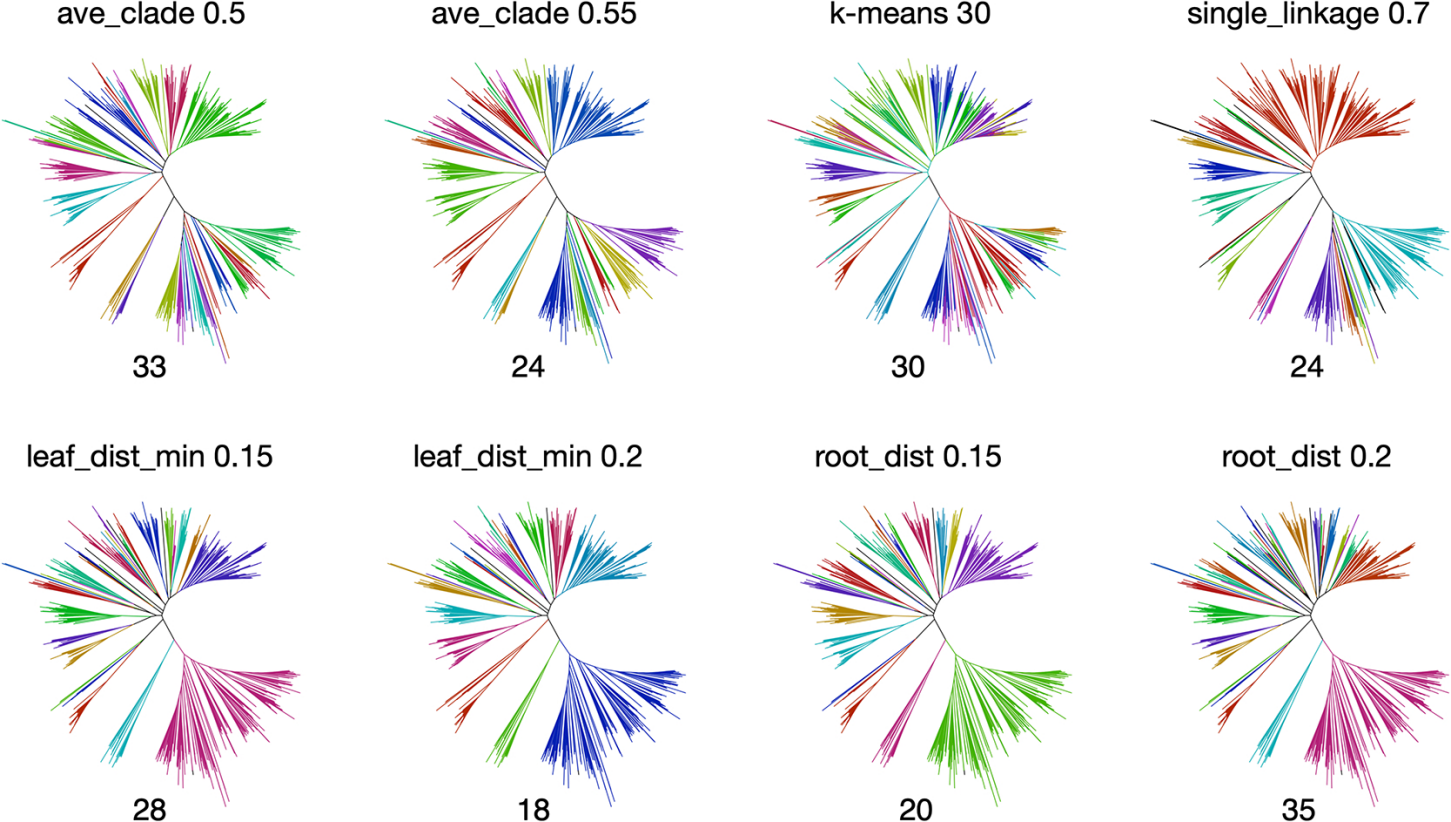
Comparison of clustering methods for phylogenetic clade classification by color. The same dataset of the homologous gene set of cluster 0 was used as in Fig. 3. Colored trees are constructed using the ggtree of R package (Xu *et al.*, 2022). The upper portion of the tree indicates the algorithm used and distance threshold, and the bottom portion shows the cluster number of the result.

**Fig. 6.**
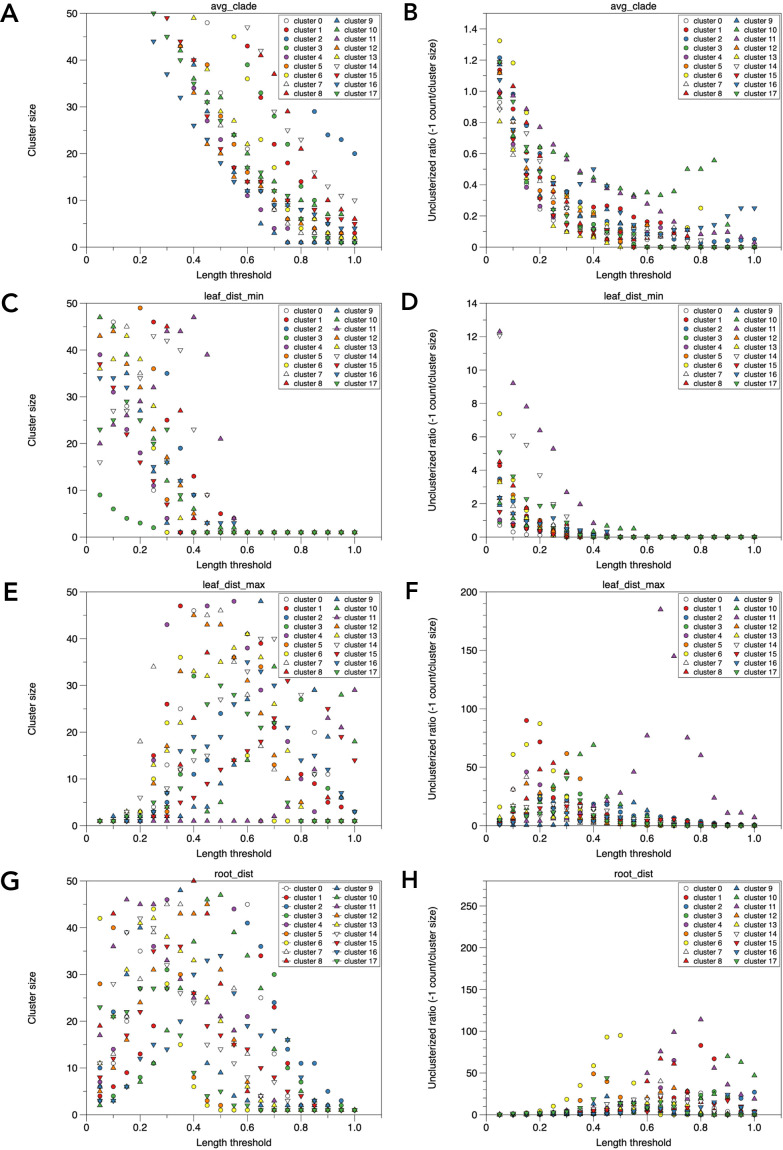
Comparison of cluster sizes and unclassified numbers among different algorithms of TreeCluster for gene clusters. Clustering is performed for clusters 0–17 using specific algorithms, varying the distance threshold from 0.05 to 1.0 in increments of 0.05. The cluster size (A, C, E, G) and resulting –1 (unclassified) ratio (divided by the cluster size) (B, D, F, H) are shown. The algorithms used are avg_clade (A, B), leaf_dist_min (C, D), leaf_dist_max (E, F), and root_dist (G, H).

The results of the GCL anal­ysis for the [NiFe]-hydrogenase large subunit showed that gene cluster distribution among clades was related to target protein function, and our constructed anal­ysis procedure showed its conservation. Although this anal­ysis is useful, it presents some challenges, particularly in the manual separation of clades because of the complexity of the criteria and unclear validity unless other highly analyzed databases for target proteins, such as HydDB, are available. In addition, performing the same anal­ysis on multiple target genes requires considerable effort and time. We introduced a computational algorithmic anal­ysis for clustering the clades of a tree. Two different algorithms for computationally separating the clades were compared. One was k-means clustering using the pairwise distance matrices of MSAs, and the other was TreeCluster (Balaban *et al.*, 2019) using constructed tree data (Newick format). A clustering anal­ysis for the clade separation of trees is generally performed by k-means clustering (Czech and Stamatakis, 2019), while TreeCluster may be useful due to the setting of universal criteria, which is not dependent on the cluster number as in k-means. Comparative clustering anal­yses of the [NiFe]-hydrogenase large subunit (Fig. 3) were performed using these algorithms (Fig. 1C). In k-means, clustering was performed on 2–50 clusters. In TreeCluster, several implemented algorithms were compared, and the results obtained were summarized as avg_clade, leaf_dist_max, leaf_dist_min, root_dist, and single_linkage. To evaluate differences between the algorithms and method conditions, the agreement of clustered genes with the HydDB class of the assigned clade ID by clustering and the control of the HydDB class distribution in the clusters were analyzed. In k-means, the over-assignment ratio to the HydDB class remained constant as the number of clusters increased (Fig. 4). The distribution of the HydDB class to the cluster ID was less likely to decrease beyond a certain point for k-means, but was even lower for the specific algorithm of TreeCluster (Fig. 4). These results suggest that avg_clade, leaf_dist, leaf_dist_min, and root_dist of TreeCluster construct favorable clusters. In addition, the clustering results shown by the colored tree indicated that when the number of clusters was increased by changing the alpha value, the relatively good increase in the classification pattern was due to ave_clade of TreeCluster rather than the k-means cluster size of 30 (Fig. 5). This procedure of constructed clustering using TreeCluster was applied to the constructed tree of gene clusters 1–17. A comparison of the results of all conditions varying the alpha factor from 0–1.0 showed that avg_clade with threshold alpha of 0.3–0.8 resulted in a range of cluster sizes from 5 to 50 (Fig. 6A). In addition, the ave_clade method showed that the number of out-groups in the cluster was always <2 (Fig. 6B). These results indicate that in contrast to the other methods, the ave_clade method constantly obtains data from all samples (Fig. 6).

### GCL anal­ysis of constructed gene clusters for the [NiFe]-hydrogenase large subunit

**Fig. 7.**
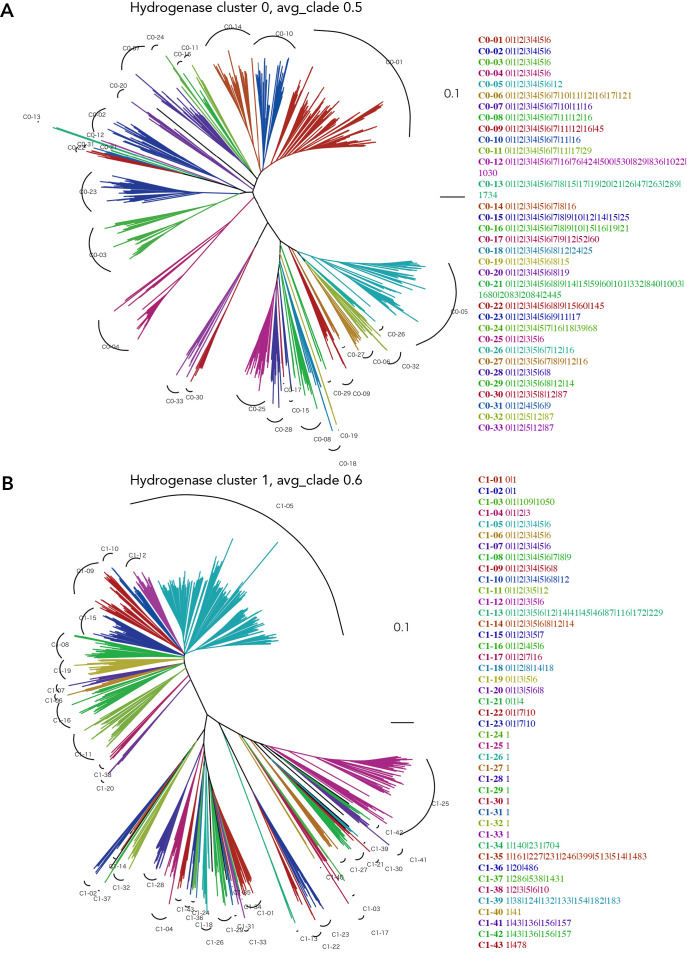
Clade clustering of the phylogenetic tree for gene clusters 0 and 1, with its conserved gene cluster list (GCL). Clade clustering is performed by TreeCluster using avg_clade with distance thresholds of 0.5 and 0.6 for clusters 0 and 1, respectively. In each cluster, 951 and 953 amino acid sequences are used for clusters 0 and 1, respectively. The clustered clade lists on the right side show the clade number and the GCL as numbers separated by “|”. The colors of the tree are identical to those of the listed individual clades. A) cluster 0. B) cluster 1.

Using TreeCluster, an automated method of clustering that showed the best performance, we analyzed the extent to which manual and automated classifications differed in their GCL anal­ysis of cluster 0. The GCL anal­ysis of cluster 0 by the applied computational automatic classification indicated separation in Group 2 of HydDB (Fig. 7A). Cluster 10, a formate dehydrogenase subunit, was identified in several clades, including 6, 7, 15, and 16 (Fig. 7A), suggesting a relationship between [NiFe]-hydrogenase and formate dehydrogenase, which forms a large complex that converts hydrogen and carbon dioxide to formate. However, the conservation of cluster 10 was scattered in the tree (Fig. 7A). Since the results of the GCL anal­ysis by manual and automatic clustering were similar (Fig. 3 and 7A), we applied this method to a GCL anal­ysis of cluster 1, which has the highest number of genes in the cluster, to consider the significance of a GCL anal­ysis of each gene cluster. The GCL anal­ysis of cluster 1 including a maturation factor for cluster 0 showed that genes may be divided into those highly related and those not related to cluster 0 (Fig. 7B). The GCL of clades 5, 6, and 7 were 0|1|2|3|4|5|6, and those of clades 1–23 also involved cluster 0. However, the GCL of other clades showed no involvement of cluster 0. In addition, the GCL including 2|3|4|5|6 in clades 5–10 exhibited high similarity to the GCL of cluster 0 (Fig. 7). These results suggest that the GCL includes gene clusters related to maturation and subunit cooperation for the genes involved in the clades, while if the gene cluster is not involved in the GCL, it indicates a weaker relationship with the target genes and the genes in the clade. We then constructed the GCL of gene clusters 4, 7, and 16, which comprise the subunits for [NiFe]-hydrogenase, to examine the relationship between each subunit and the cluster containing the maturation gene.

**Fig. 8.**
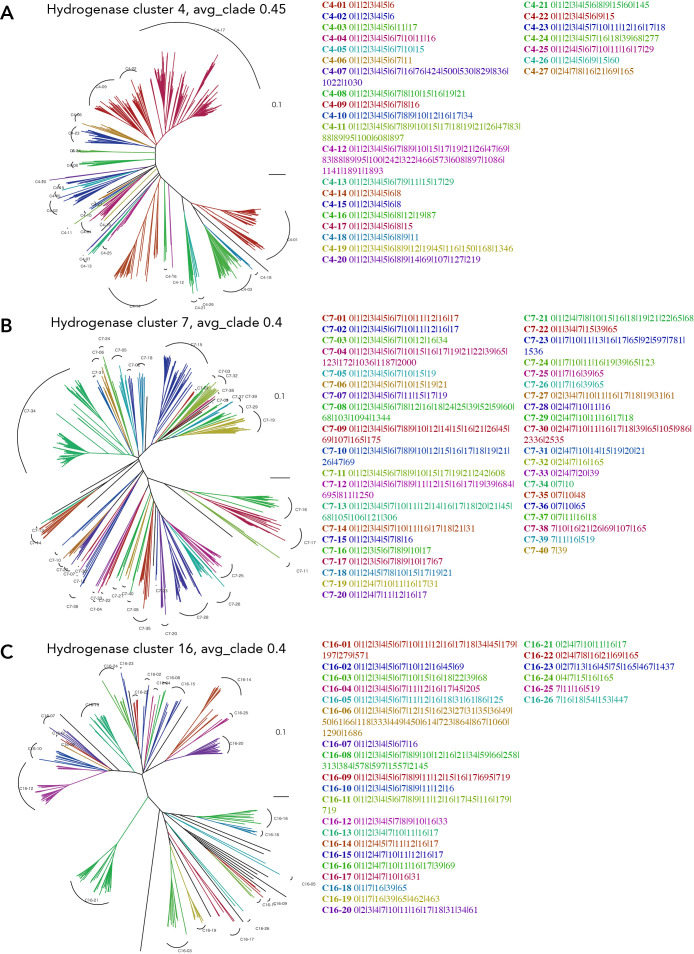
Clade clustering of the phylogenetic tree for gene clusters 4, 7, and 16, with their gene cluster list. Clustering is performed by TreeCluster with avg_clade at distance thresholds of 0.45, 0.4, and 0.4 for clusters 4, 7, and 16, respectively. In each cluster, 633, 554, and 222 amino acid sequences are used for clusters 4, 7, and 16, respectively. A) cluster 4. B) cluster 7. C) cluster 16.

The GCL of the tree of cluster 4, the [NiFe]-hydrogenase small subunit, indicated the co-conservation of gene clusters 7 and 16 (7/10) and clusters 7 and 15 (3/10) (Fig. 8A). These results suggest that the genes in cluster 4 were functionally related to clusters 7 and 16 or 15 and may be classified. However, there was no conservation tendency in cluster 12, as observed in the GCL of cluster 0, suggesting that cluster 4 was not involved in the relationship between clusters 0 and 12. The GCL of cluster 7 was classified into relationships among clusters 10, 15, 16, and 17 (Fig. 8B). In addition, the genes of cluster 7 may be clustered as relating to cluster 39, while cluster 12 was not observed as in the results on the GCL of cluster 4. In the GCL anal­ysis of cluster 16, most genes of cluster 16 were related to clusters 0, 4, and 7; however, the clade clustering of the tree of cluster 16 was unclear (Fig. 8C), suggesting that cluster 16 always works together with clusters 0, 4, and 7, and the relationship with them is required for its overall function in redox reactions with its substrates. However, within the GCL of cluster 16, clusters 33 and 39 were distinctly present in several clades (Fig. 8C), indicating the identification of other genes highly related to [NiFe]-hydrogenase by this anal­ysis.

**Fig. 9.**
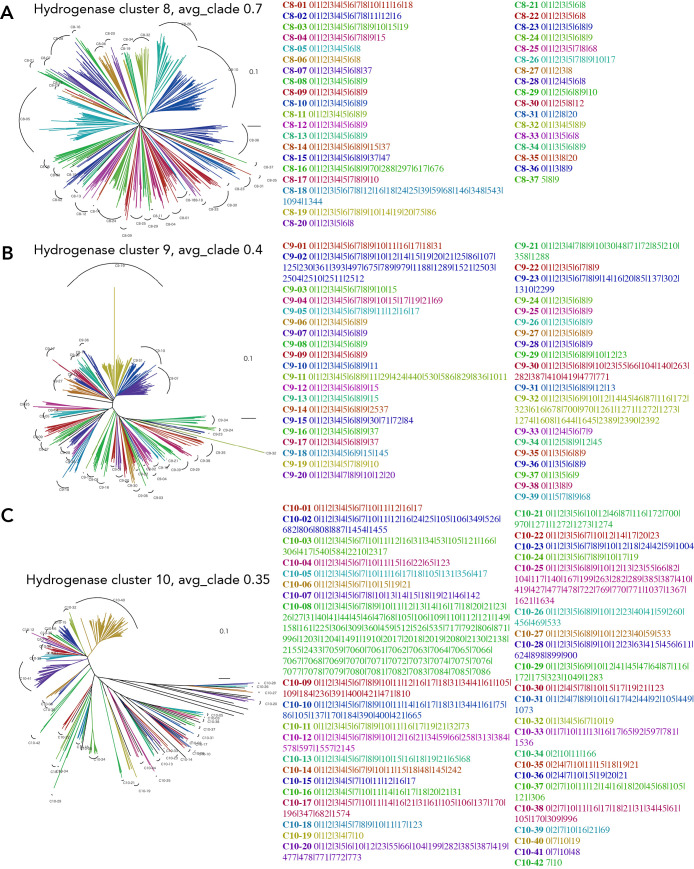
Clade clustering of the phylogenetic tree for gene clusters 8, 9, and 10, with their gene cluster list. Clustering was performed using TreeCluster with avg_clade at distance thresholds of 0.7, 0.4, and 0.35 for clusters 8, 9, and 10, respectively. In each cluster, 527, 445, and 339 amino acid sequences are used for clusters 8, 9, and 10, respectively. A) cluster 8. B) cluster 9. C) cluster 10.

In contrast, the GCL anal­ysis of cluster 0 showed the lower conservation of gene clusters 8, 9, and 10 than that of clusters 1–3, 5, and 6 (Fig. 3 and 7A). The GCLs of these less-conserved clusters were analyzed to clarify how their low conservation is related to the conservation of other gene clusters. The GCL of cluster 8 showed that almost all clustered clades conserved cluster 0, which may be classified as either with or without the inclusion of cluster 4 (Fig. 9A). In addition, all clustered clades showed the weak conservation of clusters 1–3 and 7, and a uniform distribution of clusters 5 and 6 in all clades (Fig. 9A). These results suggest that cluster 8 is a factor highly responsible for its target gene cluster (cluster 0), and the conservation of other gene clusters is not essential for its function. This was also observed in the GCL of cluster 9 (Fig. 9B), corresponding to the functional similarity of clusters 9 and 8 (Table 1). The GCL of cluster 10 showed the lower conservation of factors, but higher conservation of subunits, particularly in clusters 0, 7, and 19 observed in clustered clades 5, 9, and 15. However, cluster 4 was not observed in several clades (Fig. 9C). These results suggest a significant relationship between the respective functions of genes in each cluster and their conservation tendency, and also that functions may be considered in terms of their relationship to the target gene for anal­ysis by searching for conservation tendency around the gene to be analyzed. Therefore, a GCL anal­ysis provides advanced information for understanding the comprehensive set of genes involved in performing practical functions in cells if the genes to be analyzed are complex and require maturation.

### Automated GCL anal­ysis of the SDH subunits

**Fig. 10.**
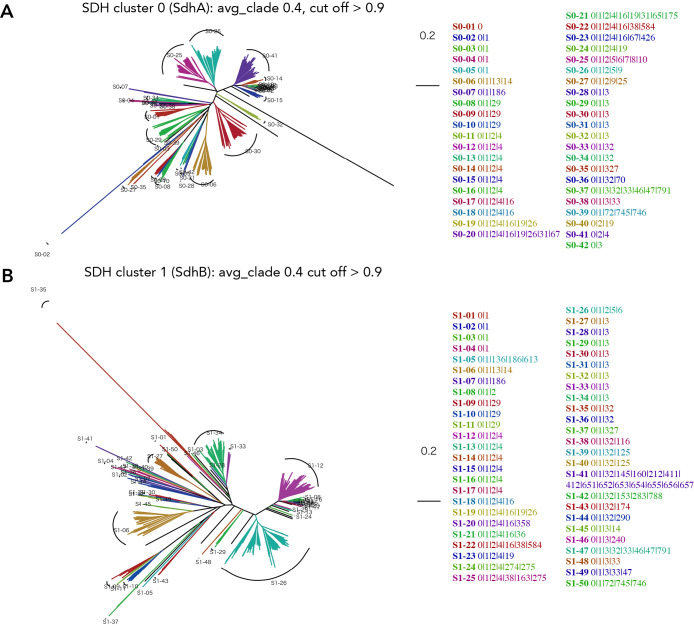
Clade clustering of the phylogenetic tree for homologs of the flavoprotein and Fe–S subunit of succinate dehydrogenase (SDH) and its gene cluster list. Clustering is performed using TreeCluster with avg_clade at a distance threshold of 0.4 with a cut-off of 0.9. The amino acid sequences used are 1,163 and 1,047 for SdhA and SdhB, respectively. Homologs for A) SdhA (cluster 0) and B) SdhB (cluster 1).

We applied our constructed procedure to a GCL anal­ysis of SDH, which has already been performed (Kosaka *et al.*, 2023). In the previous anal­ysis, the phylogenetic tree of flavoprotein subunit homolog amino acid sequences was manually separated into 8 clades (Kosaka *et al.*, 2023). However, using this procedure, it was almost automatically separated into 42 clades (Fig. 10A). Therefore, the cluster structure (the GCL in the present anal­ysis), which was unclear in the previous study, was produced by a number-based calculation, successfully reducing any subjective judgment by the analyst. The clustered clade containing SdhA of *P. thermopropionicum* showed the GCL as 0|1|29, which existed in phylogenetically close clades 8, 9, and 10 (Fig. 10A). This result is consistent with previous findings (Kosaka *et al.*, 2023). However, the cluster 9 including chaperons for flavin adenine dinucleotide (FAD) binding subunit was not observed in the results obtained with a cut-off >1.0 (data not shown). We then changed the cut-off to >0.9 and observed cluster 9 in the GCL of clades 26 and 27 (Fig. 10A), suggesting that lowering the cut-off criteria is sometimes required. In addition, we previously considered cluster 9 to be distributed among many flavoprotein subunit homologs; however, this anal­ysis revealed its conservation in specific clades (Fig. 10A), suggesting that cluster 9 was specifically acquired. Moreover, we assumed that cluster 9 was not present in other flavoprotein subunits, and also that FAD covalently binding to flavoprotein subunits, flavinylation, may be aided by other subunits, such as clusters 1, 2, 3, 4, and 29. Therefore, cluster 9, the chaperone, may not be required for the flavinylation of all flavoprotein subunits, and those flavoprotein subunits may self-flavinylate. These results support previous findings indicating that the phylogenetic distribution of flavoprotein subunits strongly correlated with the conservation of functionally important membrane subunits. In addition to the GCL anal­ysis of SDH flavoprotein subunits, we performed an anal­ysis of cluster 1, including the SDH Fe–S cluster subunits. A similar GCL pattern to that of flavoprotein subunits was observed, particularly in relation to clusters 2 and 4 or cluster 3, but not for cluster 9 (Fig. 10B), suggesting that the GCL anal­ysis reveals high functional interactions between flavoproteins and Fe–S cluster subunits, but not with the chaperone or its flavinylation.

### Additional discussion

The anal­ysis of gene-pair connections in the genome has historically been an important area of research (Rogozin *et al.*, 2002), revealing that similar genes are often located in close proximity and typically form clusters in the genome. A concept relevant to our approach is the development of the pan-genomic ortholog clustering tool that uses CGNs to cluster recurrently diverged paralogs into orthologous clusters (Fouts *et al.*, 2012). In contrast, our procedure in the present study takes the reverse strategy: rather than using CGNs to group genes, genes are clustered phylogenetically and divided into clades to analyze how CGNs are distributed, which is an unprecedented approach. Our procedure is also compatible with TreeCluster, enabling automation of the process. Previous studies already proposed methods for‍ ‍visualizing the CGNs of genes in a single genome (Gumerov and Zhulin, 2020; Price and Arkin, 2024). However, our procedure is more informative because it enables the visualization of CGNs along each gene’s molecular lineage, thereby providing insights into how gene neighborhoods evolve and also how genes may be functionally classified based on their genomic context.

In biological research, individual genes involved in specific phenomena sometimes need to be analyzed. The provision of information on what family a gene belongs to and what type of gene it is related to in advance, as well as what types of domains and motifs it has, may provide a more detailed understanding of its function. In addition, information on the relationship between genes and the target gene is sometimes required for heterologous expression in other cells to achieve functional expression. The present results indicate that the visualization of functionally-related genes to a target gene conserved in genomes combined may be achieved by the phylogenetic classification of a target gene with our constructed procedure using genomic information. The information obtained on related conserved genetic clusters for the target gene is for heterologous expression, host cell selection for expression, and gene expression platform development for desired functions within cells. This procedure may be used for already known as well as unknown genes because genomic information is static, the anal­ysis method implemented in this study basically consists of homology and clustering, and relationships are revealed independent of functional information concerning the genes. In addition, an almost automated anal­ysis may facilitate the collection of important biological information, such as the specific amino acids of SdhA and SdhC observed in a previous anal­ysis (Kosaka *et al.*, 2023). Although these functionally-related genes were only involved in the individual maturation function for the target gene, the [NiFe]-hydrogenase large subunit, genes involved in the maturation of the Fe–S cluster in many other enzymes and the biosynthetic pathway for quinone were not highly observed in the list (Table 1). Since genes directly associated with the physical function of a target gene may be extracted and visualized using this procedure, in contrast to gene clusters that are required for multiple proteins, we need other procedures to consider these gene clusters.

The construction of homologous gene sets, MSA, and tree generation require computing power and memory for calculations; however, subsequent anal­yses do not require as much machine power. Regarding phylogenetic trees constructed from protein homologs, TreeCluster (Balaban *et al.*, 2019) is a good program for automatic clade clustering, and the ave_clade method and 0.4–0.7 threshold are the optimal settings for this purpose. Further studies are needed to establish whether the criteria for listing needs to be ≤1.0. Clustering may be further reduced by considering the results of the list, and conversely, singularities may be searched. In addition, although the tree calculation is only based on the NJ method and the distance method in this study, it may be interesting to compare the same with the trait state method.

## Citation

Kosaka, T., and Matsutani, M. (2025) Using Phylogeny and a Conserved Genomic Neighborhood Analysis to Extract and Visualize Gene Sets Involved in Target Gene Function: The Case of [NiFe]-hydrogenase and Succinate Dehydrogenase. *Microbes Environ ***40**: ME25018.

https://doi.org/10.1264/jsme2.ME25018

## Supplementary Material

Supplementary Material

## Figures and Tables

**Table 1.** Constructed conserved gene clusters (ID ≤50)

Cluster ID	Gene count	Name (highest Pfam hit)	Pfam short (%) >10%	*P. thermopropionicum*	*E. coli*
0	951	Nickel-dependent hydrogenase	NiFeSe_Hases (100)	PTH_1702	b0973:b2994
1	953	AIR synthase related protein, N-terminal domain	AIRS (100); AIRS_C (99)	PTH_1699	b2730
2	758	Hydrogenase maturation protease	HycI (87); - (13)	PTH_1700	b0975:b2717:b2993
3	748	HupF/HypC family	HupF_HypC (100)	PTH_1697	b2728:b2990
4	633	NADH ubiquinone oxidoreductase, 20 Kd subunit	Oxidored_q6 (100); NiFe_hyd_SSU_C (100); TAT_signal (19)	PTH_1701	b0972:b2997
5	615	Hydrogenase formation hypA family	HypD (100)	PTH_1698	b2729
6	600	HypF Kae1-like domain	HypF_C (100); Sua5_yciO_yrdC (97); zf-HYPF (96); Acylphosphatase (96); TsaD (40)	PTH_1696	b2712
7	554	4Fe-4S dicluster domain	Fer4_7 (100); Fer4_11 (100); Fer4_9 (97); Fer4_10 (97); Fer4_6 (94); Fer4 (94); Fer4_2 (94); Fer4_21 (75); Fer4_4 (61); Fer4_3 (40); Fer4_8 (34); Fer4_16 (31); Form-deh_trans (22); Fer4_17 (17)	PTH_0669: PTH_1703: PTH_1713	b2713:b2724:b2996:b3893
8	527	Hydrogenase/urease nickel incorporation, metallochaperone, hypA	HypA (100)	PTH_1694	b2726:b2991
9	445	CobW/HypB/UreG, nucleotide-binding domain	cobW (100); MeaB (18); MobB (11); RsgA_GTPase (11)		b2727
10	339	Molybdopterin oxidoreductase	Molybdopterin (100); Molydop_binding (93); Molybdop_Fe4S4 (65); TAT_signal (14)	PTH_1712	b3894
11	312	Histidine kinase-, DNA gyrase B-, and HSP90-like ATPase	HATPase_c (99); HisKA (88); PAS (49); PAS_4 (48); PAS_9 (47); PAS_8 (32); Response_reg (27); HATPase_c_5 (24); HAMP (23); PAS_3 (20); HATPase_c_3 (14)		
12	306	NADH ubiquinone oxidoreductase, 20 Kd subunit	Oxidored_q6 (100)		
13	300	Tautomerase enzyme	Tautomerase (100); Tautomerase_2 (93)	PTH_1705	
14	260	ABC transporter	ABC_tran (100); AAA_21 (89); SMC_N (65); AAA_29 (37); AAA_16 (22); AAA_22 (21); AAA_23 (14); RsgA_GTPase (13)		
15	224	Prokaryotic cytochrome b561	Ni_hydr_CYTB (100); DUF4405 (84)		b0974
16	222	Polysulphide reductase, NrfD	NrfD (82); - (18)	PTH_1704: PTH_1714	b2995
17	216	Sigma-54 interaction domain	Sigma54_activat (100); Sigma54_activ_2 (100); AAA_5 (94); HTH_8 (87); AAA (76); Response_reg (56); Mg_chelatase (38); HTH_50 (19); PAS (19); PAS_4 (18); GAF (18); GAF_2 (18); PAS_9 (17); PAS_8 (16); AAA_2 (15); GAF_3 (15)		b2709:b2731
18	183	Response regulator receiver domain	Response_reg (97); Trans_reg_C (25); GerE (20); Sigma70_r4_2 (16); LytTR (11)		
19	185	Prokaryotic cytochrome b561	Ni_hydr_CYTB (97); DUF4405 (66)		b3892
20	172	NUBPL iron-transfer P-loop NTPase	ParA (100); CbiA (99); AAA_31 (98); MipZ (88); ArsA_ATPase (81); Fer4_NifH (74); FeS_assembly_P (26); VirC1 (22); AAA_26 (20); CBP_BcsQ (17); AAA_25 (12); MeaB (10)	PTH_1695	b2113
21	172	Protein involved in formate dehydrogenase formation	FdhE (100)	PTH_1693	b3891
22	128	Bacterial regulatory helix-turn-helix protein, lysR family	HTH_1 (99); LysR_substrate (98)		
23	105	NADH-ubiquinone oxidoreductase-F iron-sulfur binding region	NADH_4Fe-4S (100); Complex1_51K (100); 2Fe-2S_thioredx (84); SLBB (77); Fer4_7 (14); Fer4 (14); Fer4_9 (13); Fer4_10 (13); Fer4_21 (12); Fer4_2 (11); Fer4_6 (11)		
24	107	Oxidoreductase NAD-binding domain	NAD_binding_1 (98); DHODB_Fe-S_bind (92); FAD_binding_6 (45)		
25	95	4Fe-4S dicluster domain	Fer4_22 (100); Fer4_7 (92); Fer4_2 (91); Fer4_10 (88); Fer4_8 (86); Fer4_9 (84); Fer4_17 (77); Fer4_18 (68); Fer4_21 (66); Fer4 (54); Fer4_16 (41); Fer4_6 (26)		
26	96	Molybdopterin oxidoreductase Fe4S4 domain	Molybdop_Fe4S4 (100); Molybdopterin (59); TAT_signal (20)	PTH_1711	
27	88	2Fe-2S iron-sulfur cluster binding domain	Fer2_4 (100); NADH-G_4Fe-4S_3 (99); Fer4_15 (80); Fer2 (74); Fer4 (65); Fer4_10 (59); Fer4_9 (59); Fer4_7 (59); Fer4_6 (59); Fer4_16 (59); Fer4_2 (53); Fer4_8 (49); Fer4_21 (48); Fer4_18 (22); Fer4_4 (19); Fer4_13 (15); Fer4_17 (13)		
28	87	Enoyl-(Acyl carrier protein) reductase	adh_short_C2 (100); adh_short (100); KR (89); Epimerase (40); NAD_binding_10 (14); DUF1776 (10)		
29	87	Methyl-accepting chemotaxis protein (MCP) signalling domain	MCPsignal (99); HAMP (69); 4HB_MCP_1 (20); TarH (11); Cache_3-Cache_2 (10)	PTH_0667	
30	75	NifU-like domain	NifU (91); Rieske (49); Rieske_2 (43)		
31	75	4Fe-4S dicluster domain	Fer4_8 (97); Fer4_17 (97); CCG (96); Fer4_7 (79); Fer4_10 (71); Fer4_9 (65); Fer4 (57); Fer4_21 (35); Fer4_2 (33); Fer4_6 (32); Fer4_18 (20); Fer4_16 (20); Fer4_4 (17)		
32	75	FdhD/NarQ family	FdhD-NarQ (100)		b3895
33	75	[NiFe]-hydrogenase assembly, chaperone, HybE	NiFe-hyd_HybE (100); Rubredoxin (21); PHD_4 (13)		b2992
34	71	Universal stress protein family	Usp (100)		
35	69	Nickel-dependent hydrogenase	NiFeSe_Hases (65); - (35)		
36	70	Alpha/beta hydrolase family	Abhydrolase_6 (100); Abhydrolase_1 (99); Hydrolase_4 (99); Ndr (57); Thioesterase (56); Abhydrolase_5 (46); Esterase (33); PGAP1 (26); DUF915 (19); UPF0227 (19); LIDHydrolase (17); Ser_hydrolase (17); Abhydrolase_11 (14); Peptidase_S9 (14); DUF1057 (13)		
37	68	SIS domain	SIS_2 (94); SIS (66)		
38	65	Fumarylacetoacetate (FAA) hydrolase family	FAA_hydrolase (92)		
39	64	Molydopterin dinucleotide binding domain	Molydop_binding (100); Molybdopterin (100); Molybdop_Fe4S4 (58); TAT_signal (28)		
40	57	Thioredoxin-like [2Fe-2S] ferredoxin	2Fe-2S_thioredx (100)		
41	58	CBS domain	CBS (100); CP12 (24)		
42	56	NAD dependent epimerase/dehydratase family	Epimerase (96); GDP_Man_Dehyd (84); 3Beta_HSD (82); RmlD_sub_bind (82); NAD_binding_4 (77); NAD_binding_10 (75); Polysacc_synt_2 (63); NmrA (48); KR (32); adh_short (32)		
43	56	Acetyltransferase (GNAT) domain	Acetyltransf_7 (100); Acetyltransf_1 (100); Acetyltransf_10 (98); FR47 (77); Acetyltransf_3 (59); Acetyltransf_9 (55); Acetyltransf_4 (36); Acetyltransf_8 (21); Acetyltransf_6 (14); Acetyltransf_CG (13)		
44	54	Haloacid dehalogenase-like hydrolase	HAD_2 (100); Hydrolase (96); Hydrolase_like (83); HAD (52)		
45	52	Methyl-viologen-reducing hydrogenase, delta subunit	FlpD (100)		
46	54	4Fe-4S dicluster domain	Fer4_9 (96); Fer4_7 (94); Fer4_10 (93); Fer4 (93); Fer4_21 (91); Fer4_2 (87); Fer4_6 (87); Fer4_16 (85); Fer4_4 (65); Fer4_17 (59); Fer4_8 (56); Fer4_13 (48); Fer4_15 (39); Fer4_22 (39); Fer4_3 (26)		
47	54	Radical SAM superfamily	Radical_SAM (100); Fer4_14 (78); Fer4_12 (70); SPASM (63); Mob_synth_C (22)		
48	53	Polysulphide reductase, NrfD	NrfD (100); NrfD_2 (30)		
49	46	HupH hydrogenase expression protein, C-terminal conserved region	HupH_C (100)		b0977
50	50	EAL domain	EAL (98); GGDEF (96); PAS_9 (56); PAS_4 (56); PAS (56); PAS_3 (50); PAS_8 (44); GAF_2 (16)	PTH_0666	

## References

[B1] Anjos, W.F., Lanes, G.C., Azevedo, V.A., and Santos, A.R. (2021) GENPPI: standalone software for creating protein interaction networks from genomes. BMC Bioinf 22: 596. 10.1186/s12859-021-04501-0 PMC8680239 34915867

[B2] Balaban, M., Moshiri, N., Mai, U., Jia, X., and Mirarab, S. (2019) TreeCluster: Clustering biological sequences using phylogenetic trees. PLoS One 14: e0221068. 31437182 10.1371/journal.pone.0221068 PMC6705769

[B3] Beaton, S.E., Evans, R.M., Finney, A.J., Lamont, C.M., Armstrong, F.A., Sargent, F., and Carr, S.B. (2018) The structure of hydrogenase-2 from *Escherichia coli*: implications for H_2_-driven proton pumping. Biochem J 475: 1353–1370. 29555844 10.1042/BCJ20180053 PMC5902676

[B4] Blum, M., Andreeva, A., Florentino, L.C., Chuguransky, S.R., Grego, T., Hobbs, E., et al. (2025) InterPro: the protein sequence classification resource in 2025. Nucleic Acids Res 53: D444–D456. 39565202 10.1093/nar/gkae1082 PMC11701551

[B5] Caetano, T., Krawczyk, J.M., Mösker, E., Süssmuth, R.D., and Mendo, S. (2011) Heterologous expression, biosynthesis, and mutagenesis of type II lantibiotics from *Bacillus licheniformis* in *Escherichia coli*. Chem Biol 18: 90–100. 21276942 10.1016/j.chembiol.2010.11.010

[B6] Camacho, C., Coulouris, G., Avagyan, V., Ma, N., Papadopoulos, J., Bealer, K., and Madden, T.L. (2009) BLAST+: architecture and applications. BMC Bioinf 10: 421. 10.1186/1471-2105-10-421 PMC2803857 20003500

[B7] Czech, L., and Stamatakis, A. (2019) Scalable methods for analyzing and visualizing phylogenetic placement of metagenomic samples. PLoS One 14: e0217050. 31136592 10.1371/journal.pone.0217050 PMC6538146

[B8] Eddy, S.R. (2011) Accelerated profile HMM searches. PLoS Comput Biol 7: e1002195. 22039361 10.1371/journal.pcbi.1002195 PMC3197634

[B9] Edgar, R.C. (2004a) MUSCLE: multiple sequence alignment with high accuracy and high throughput. Nucleic Acids Res 32: 1792–1797. 15034147 10.1093/nar/gkh340 PMC390337

[B10] Edgar, R.C. (2004b) MUSCLE: a multiple sequence alignment method with reduced time and space complexity. BMC Bioinf 5: 113. 10.1186/1471-2105-5-113 PMC517706 15318951

[B11] Esch, R., and Merkl, R. (2020) Conserved genomic neighborhood is a strong but no perfect indicator for a direct interaction of microbial gene products. BMC Bioinf 21: 5. 10.1186/s12859-019-3200-z PMC6941341 31900122

[B12] Fang, G., Rocha, E.P.C., and Danchin, A. (2008) Persistence drives gene clustering in bacterial genomes. BMC Genomics 9: 4. 18179692 10.1186/1471-2164-9-4 PMC2234087

[B13] Fondi, M., Pini, F., Riccardi, C., Gemo, P., and Brilli, M. (2024) A new selective force driving metabolic gene clustering. mSystems 9: e0096024. 39465945 10.1128/msystems.00960-24 PMC11629862

[B14] Fouts, D.E., Brinkac, L., Beck, E., Inman, J., and Sutton, G. (2012) PanOCT: automated clustering of orthologs using conserved gene neighborhood for pan-genomic anal­ysis of bacterial strains and closely related species. Nucleic Acids Res 40: e172. 22904089 10.1093/nar/gks757 PMC3526259

[B15] Greening, C., Biswas, A., Carere, C.R., Jackson, C.J., Taylor, M.C., Stott, M.B., et al. (2016) Genomic and metagenomic surveys of hydrogenase distribution indicate H2 is a widely utilised energy source for microbial growth and survival. ISME J 10: 761–777. 26405831 10.1038/ismej.2015.153 PMC4817680

[B16] Gumerov, V.M., and Zhulin, I.B. (2020) TREND: a platform for exploring protein function in prokaryotes based on phylogenetic, domain architecture and gene neighborhood anal­yses. Nucleic Acids Res 48: W72–W76. 32282909 10.1093/nar/gkaa243 PMC7319448

[B17] Haft, D.H., Loftus, B.J., Richardson, D.L., Yang, F., Eisen, J.A., Paulsen, I.T., and White, O. (2001) TIGRFAMs: a protein family resource for the functional identification of proteins. Nucleic Acids Res 29: 41–43. 11125044 10.1093/nar/29.1.41 PMC29844

[B18] Inoue, H., Wakai, S., Nishihara, H., and Sambongi, Y. (2011) Heterologous synthesis of cytochrome *c*’ by *Escherichia coli* is not dependent on the System I cytochrome *c* biogenesis machinery. FEBS J 278: 2341–2348. 21554540 10.1111/j.1742-4658.2011.08155.x

[B19] Kanehisa, M., and Goto, S. (2000) KEGG: kyoto encyclopedia of genes and genomes. Nucleic Acids Res 28: 27–30. 10592173 10.1093/nar/28.1.27 PMC102409

[B20] Kosaka, T., Tsushima, Y., Shiota, Y., Ishiguchi, T., Matsushita, K., Matsutani, M., and Yamada, M. (2023) Membrane potential-requiring succinate dehydrogenase constitutes the key to propionate oxidation and is unique to syntrophic propionate-oxidizing bacteria. Microbes Environ 38: ME22111. 37081625 10.1264/jsme2.ME22111 PMC10308238

[B21] Kuchenreuther, J.M., Grady-Smith, C.S., Bingham, A.S., George, S.J., Cramer, S.P., and Swartz, J.R. (2010) High-yield expression of heterologous [FeFe] hydrogenases in *Escherichia coli*. PLoS One 5: e15491. 21124800 10.1371/journal.pone.0015491 PMC2991362

[B22] Kumar, S., Stecher, G., Li, M., Knyaz, C., and Tamura, K. (2018) MEGA X: Molecular Evolutionary Genetics Analysis across Computing Platforms. Mol Biol Evol 35: 1547–1549. 29722887 10.1093/molbev/msy096 PMC5967553

[B23] Lacasse, M.J., and Zamble, D.B. (2016) [NiFe]-hydrogenase maturation. Biochemistry 55: 1689–1701. 26919691 10.1021/acs.biochem.5b01328

[B24] Lawrence, J.G., and Roth, J.R. (1996) Selfish operons: horizontal transfer may drive the evolution of gene clusters. Genetics 143: 1843–1860. 8844169 10.1093/genetics/143.4.1843 PMC1207444

[B25] Maier, J.A., Ragozin, S., and Jeltsch, A. (2015) Identification, cloning and heterologous expression of active [NiFe]-hydrogenase 2 from *Citrobacter* sp. SG in *Escherichia coli*. J Biotechnol 199: 1–8. 25678135 10.1016/j.jbiotec.2015.01.025

[B26] McNeil, M.B., Clulow, J.S., Wilf, N.M., Salmond, G.P., and Fineran, P.C. (2012) SdhE is a conserved protein required for flavinylation of succinate dehydrogenase in bacteria. J Biol Chem 287: 18418–18428. 22474332 10.1074/jbc.M111.293803 PMC3365757

[B27] Miki, K., Atomi, H., and Watanabe, S. (2020) Structural insight into [NiFe] hydrogenase maturation by transient complexes between Hyp proteins. Acc Chem Res 53: 875–886. 32227866 10.1021/acs.accounts.0c00022

[B28] Paysan-Lafosse, T., Andreeva, A., Blum, M., Chuguransky, S.R., Grego, T., Pinto, B.L., et al. (2025) The Pfam protein families database: embracing AI/ML. Nucleic Acids Res 53: D523–D534. 39540428 10.1093/nar/gkae997 PMC11701544

[B29] Posewitz, M.C., King, P.W., Smolinski, S.L., Zhang, L., Seibert, M., and Ghirardi, M.L. (2004) Discovery of two novel radical S-adenosylmethionine proteins required for the assembly of an active [Fe] hydrogenase. J Biol Chem 279: 25711–25720. 15082711 10.1074/jbc.M403206200

[B30] Powell, S., Szklarczyk, D., Trachana, K., Roth, A., Kuhn, M., Muller, J., et al. (2012) eggNOG v3.0: orthologous groups covering 1133 organisms at 41 different taxonomic ranges. Nucleic Acids Res 40: D284–D289. 22096231 10.1093/nar/gkr1060 PMC3245133

[B31] Price, M.N., and Arkin, A.P. (2024) A fast comparative genome browser for diverse bacteria and archaea. PLoS One 19: e0301871. 38593165 10.1371/journal.pone.0301871 PMC11003636

[B32] Robin, V., Bodein, A., Scott-Boyer, M.-P., Leclercq, M., Périn, O., and Droit, A. (2022) Overview of methods for characterization and visualization of a protein-protein interaction network in a multi-omics integration context. Front Mol Biosci 9: 962799. 36158572 10.3389/fmolb.2022.962799 PMC9494275

[B33] Rogozin, I.B., Makarova, K.S., Murvai, J., Czabarka, E., Wolf, Y.I., Tatusov, R.L., et al. (2002) Connected gene neighborhoods in prokaryotic genomes. Nucleic Acids Res 30: 2212–2223. 12000841 10.1093/nar/30.10.2212 PMC115289

[B34] Shendure, J., Balasubramanian, S., Church, G.M., Gilbert, W., Rogers, J., Schloss, J.A., and Waterston, R.H. (2017) DNA sequencing at 40: past, present and future. Nature 550: 345–353. 29019985 10.1038/nature24286

[B35] Shiota, Y., and Kosaka, T. (2025) Insight on flavinylation and functioning factor in Type B succinate dehydrogenase from Gram-positive bacteria. Biosci Biotechnol Biochem 89: 832–840. 40053489 10.1093/bbb/zbaf026

[B36] Soboh, B., Adrian, L., and Stripp, S.T. (2022) An in vitro reconstitution system to monitor iron transfer to the active site during the maturation of [NiFe]-hydrogenase. J Biol Chem 298: 102291. 35868564 10.1016/j.jbc.2022.102291 PMC9418501

[B37] Søndergaard, D., Pedersen, C.N., and Greening, C. (2016) HydDB: A web tool for hydrogenase classification and anal­ysis. Sci Rep 6: 34212. 27670643 10.1038/srep34212 PMC5037454

[B38] Sun, J., Hopkins, R.C., Jenney, F.E., McTernan, P.M., and Adams, M.W. (2010) Heterologous expression and maturation of an NADP-dependent [NiFe]-hydrogenase: a key enzyme in biofuel production. PLoS One 5: e10526. 20463892 10.1371/journal.pone.0010526 PMC2865534

[B39] Szklarczyk, D., Kirsch, R., Koutrouli, M., Nastou, K., Mehryary, F., Hachilif, R., et al. (2023) The STRING database in 2023: protein-protein association networks and functional enrichment anal­yses for any sequenced genome of interest. Nucleic Acids Res 51: D638–D646. 36370105 10.1093/nar/gkac1000 PMC9825434

[B40] Tamura, K., Dudley, J., Nei, M., and Kumar, S. (2007) MEGA4: Molecular Evolutionary Genetics Analysis (MEGA) software version 4.0. Mol Biol Evol 24: 1596–1599. 17488738 10.1093/molbev/msm092

[B41] Thöny-Meyer, L., Fischer, F., Künzler, P., Ritz, D., and Hennecke, H. (1995) *Escherichia coli* genes required for cytochrome c maturation. J Bacteriol 177: 4321–4326. 7635817 10.1128/jb.177.15.4321-4326.1995 PMC177179

[B42] Törönen, P., and Holm, L. (2022) PANNZER-A practical tool for protein function prediction. Protein Sci 31: 118–128. 34562305 10.1002/pro.4193 PMC8740830

[B43] Uhlen, M., and Quake, S.R. (2023) Sequential sequencing by synthesis and the next-generation sequencing revolution. Trends Biotechnol 41: 1565–1572. 37482467 10.1016/j.tibtech.2023.06.007

[B44] UniProt Consortium (2025) UniProt: the Universal Protein Knowledgebase in 2025. Nucleic Acids Res 53: D609–D617. 39552041 10.1093/nar/gkae1010 PMC11701636

[B45] van Dongen, S., and Abreu-Goodger, C. (2012) Using MCL to extract clusters from networks. Methods Mol Biol 804: 281–295. 22144159 10.1007/978-1-61779-361-5_15

[B46] Wang, J., Chitsaz, F., Derbyshire, M.K., Gonzales, N.R., Gwadz, M., Lu, S., et al. (2023) The conserved domain database in 2023. Nucleic Acids Res 51: D384–D388. 36477806 10.1093/nar/gkac1096 PMC9825596

[B47] Xu, S., Li, L., Luo, X., Chen, M., Tang, W., Zhan, L., et al. (2022) Ggtree: A serialized data object for visualization of a phylogenetic tree and annotation data. iMeta 1: 82. 10.1002/imt2.56 PMC10989815 38867905

[B48] Yu, G., Smith, D.K., Zhu, H., Guan, Y., and Lam, T.T. (2017) GGTREE: an R package for visualization and annotation of phylogenetic trees with their covariates and other associated data. Methods Ecol Evol 8: 28–36.

[B49] Zaharia, A., Labedan, B., Froidevaux, C., and Denise, A. (2019) CoMetGeNe: mining conserved neighborhood patterns in metabolic and genomic contexts. BMC Bioinf 20: 19. 10.1186/s12859-018-2542-2 PMC6327494 30630411

[B50] Zheng, J., Ge, Q., Yan, Y., Zhang, X., Huang, L., and Yin, Y. (2023) dbCAN3: automated carbohydrate-active enzyme and substrate annotation. Nucleic Acids Res 51: W115–W121. 37125649 10.1093/nar/gkad328 PMC10320055

