# Supplementary material for: Using Phylogeny and a Conserved Genomic Neighborhood Analysis to Extract and Visualize Gene Sets Involved in Target Gene Function: The Case of [NiFe]-hydrogenase and Succinate Dehydrogenase
Source: Microbes Environ. 2025 Nov 13;40(4):ME25018. doi: 10.1264/jsme2.ME25018 (PMC12727194; doi:10.1264/jsme2.ME25018)
Supplement: Supplementary file 1 — Supplementary Material [file 40_25018_s1.pdf]

Fig. S1

	max number
Cluster ID	7879
Gene count	953

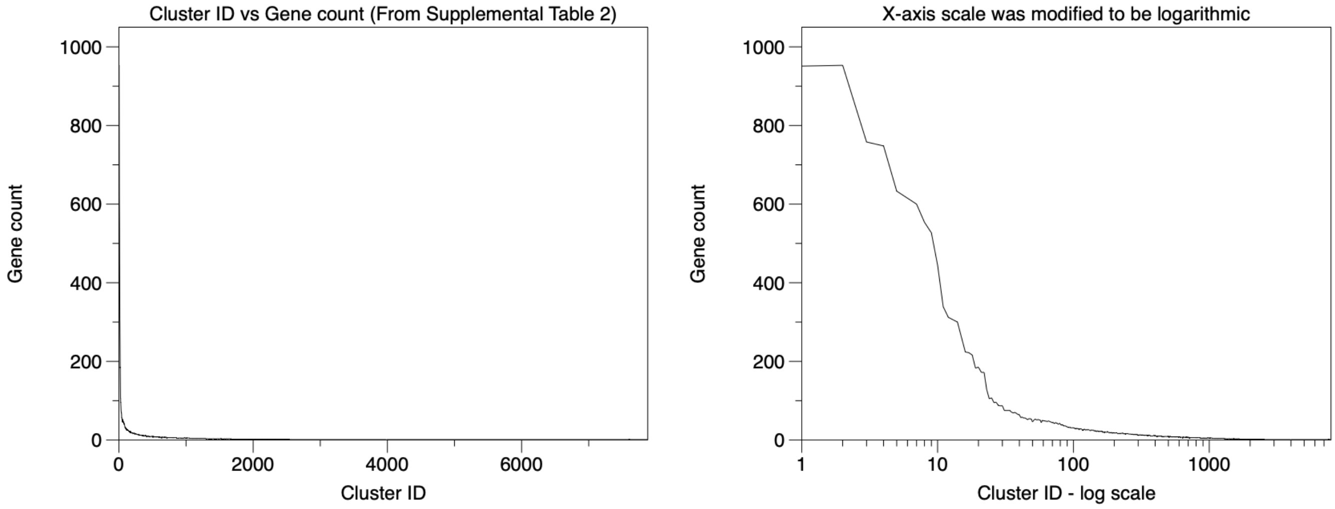

Fig. S1. Summary of constructed gene clusters. Top panel: Table for max number of cluster ID and gene count. Left panel: The plot graph for Cluster ID vs Gene count. Right panel: The modified left panel altered the x-axis to a logarithmic scale.

Table S1. Content of selected genome data

Name	Phylum	Accession
Bacillus pseudofirmus OF4	Bacillota	GCF_000005825.2
Escherichia coli str. K-12 substr. MG1655	Pseudomonadota	GCF_000005845.2
Methanococcus voltae A3	Methanobacteriota	GCF_000006175.1
Corynebacterium jeikeium K411	Actinomycetota	GCF_000006605.1
Chlamydia muridarum str. Nigg	Chlamydiota	GCF_000006685.1
Xylella fastidiosa 9a5c	Pseudomonadota	GCF_000006725.1
Vibrio cholerae O1 biovar El Tor str. N16961	Pseudomonadota	GCF_000006745.1
Pseudomonas aeruginosa PAO1	Pseudomonadota	GCF_000006765.1
Streptococcus pyogenes M1 GAS	Bacillota	GCF_000006785.2
Halobacterium salinarum NRC-1	Methanobacteriota	GCF_000006805.1
Neisseria gonorrhoeae FA 1090	Pseudomonadota	GCF_000006845.1
Lactococcus lactis subsp. lactis Il1403	Bacillota	GCF_000006865.1
Caulobacter crescentus CB15	Pseudomonadota	GCF_000006905.1
Shigella flexneri 2a str. 301	Pseudomonadota	GCF_000006925.2
Salmonella enterica subsp. enterica serovar Typhimurium str. LT2	Pseudomonadota	GCF_000006945.2
Sinorhizobium meliloti 1021	Pseudomonadota	GCF_000006965.1
Chlorobium tepidum TLS	Chlorobiota	GCF_000006985.1
Rickettsia conorii str. Malish 7	Pseudomonadota	GCF_000007025.1
Caldanaerobacter subterraneus subsp. tengcongensis MB4	Bacillota	GCF_000007085.1
Brucella melitensis bv. 1 str. 16M	Pseudomonadota	GCF_000007125.1
Xanthomonas campestris pv. campestris str. ATCC 33913	Pseudomonadota	GCF_000007145.1
Pyrococcus furiosus DSM 3638	Methanobacteriota	GCF_000007305.1
Fusobacterium nucleatum subsp. nucleatum ATCC 25586	Fusobacteriota	GCF_000007325.1
Buchnera aphidicola str. Sg (Schizaphis graminum)	Pseudomonadota	GCF_000007365.1
Tropheryma whipplei str. Twist	Actinomycetota	GCF_000007485.1
Bifidobacterium longum NCC2705	Actinomycetota	GCF_000007525.1
Pseudomonas putida KT2440	Pseudomonadota	GCF_000007565.2
Clostridium tetani E88	Bacillota	GCF_000007625.1
Staphylococcus epidermidis ATCC 12228	Bacillota	GCF_000007645.1
Chromobacterium violaceum ATCC 12472	Pseudomonadota	GCF_000007705.1
Mannheimia succiniciproducens MBEL55E	Pseudomonadota	GCF_000007745.1
Coxiella burnetii RSA 493	Pseudomonadota	GCF_000007765.2
Enterococcus faecalis V583	Bacillota	GCF_000007785.1
Pseudomonas syringae pv. tomato str. DC3000	Pseudomonadota	GCF_000007805.1
Bacillus cereus ATCC 14579	Bacillota	GCF_000007825.1
Mycobacterium avium subsp. paratuberculosis K-10	Actinomycetota	GCF_000007865.1
Helicobacter hepaticus ATCC 51449	Campylobacterota	GCF_000007905.1
Prochlorococcus marinus subsp. marinus str. CCMP1375	Cyanobacteriota	GCF_000007925.1
[Haemophilus] ducreyi 35000HP	Pseudomonadota	GCF_000007945.1
Geobacter sulfurreducens PCA	Thermodesulfobacteriota	GCF_000007985.2
Wolbachia endosymbiont of Drosophila melanogaster	Pseudomonadota	GCF_000008025.1
Rickettsia typhi str. Wilmington	Pseudomonadota	GCF_000008045.1
Treponema denticola ATCC 35405	Spirochaetota	GCF_000008185.1
Mycoplasma hyopneumoniae J	Mycoplasmatota	GCF_000008205.1
Picrophilus torridus DSM 9790	Thermoplasmatota	GCF_000008265.1
Mesoplasma florum L1	Mycoplasmatota	GCF_000008305.1
Methylococcus capsulatus str. Bath	Pseudomonadota	GCF_000008325.1
Propionibacterium acnes KPA171202	Actinomycetota	GCF_000008345.1
Idiomarina loihiensis L2TR	Pseudomonadota	GCF_000008465.1
Legionella pneumophila subsp. pneumophila str. Philadelphia 1	Pseudomonadota	GCF_000008485.1

Thermotoga maritima MSB8	Thermotogota	GCF_000008545.1
Deinococcus radiodurans R1	Deinococcota	GCF_000008565.1
Aquifex aeolicus VF5	Aquificota	GCF_000008625.1
Methanothermobacter thermautotrophicus str. Delta H	Methanobacteriota	GCF_000008645.1
Archaeoglobus fulgidus DSM 4304	Methanobacteriota	GCF_000008665.1
Borrelia burgdorferi B31	Spirochaetota	GCF_000008685.2
Wigglesworthia glossinidia endosymbiont of Glossina brevipalpis	Pseudomonadota	GCF_000008885.1
Lactobacillus salivarius UCC118	Bacillota	GCF_000008925.1
Francisella tularensis subsp. tularensis SCHU S4	Pseudomonadota	GCF_000008985.1
Bacillus subtilis subsp. subtilis str. 168	Bacillota	GCF_000009045.1
Yersinia pestis CO92	Pseudomonadota	GCF_000009065.1
Campylobacter jejuni subsp. jejuni NCTC 11168 = ATCC 700819	Campylobacterota	GCF_000009085.1
Ralstonia solanacearum GMI1000	Pseudomonadota	GCF_000009125.1
Nitrosomonas europaea ATCC 19718	Pseudomonadota	GCF_000009145.1
Clostridioides difficile 630	Bacillota	GCF_000009205.2
Yersinia enterocolitica subsp. enterocolitica 8081	Pseudomonadota	GCF_000009345.1
Alcanivorax borkumensis SK2	Pseudomonadota	GCF_000009365.1
Streptomyces avermitilis MA-4680 = NBRC 14893	Actinomycetota	GCF_000009765.2
Onion yellows phytoplasma OY-M	Mycoplasmatota	GCF_000009845.1
Symbiobacterium thermophilum IAM 14863	Bacillota	GCF_000009905.1
Bacteroides fragilis YCH46	Bacteroidota	GCF_000009925.1
Thermococcus kodakarensis KOD1	Methanobacteriota	GCF_000009965.1
Magnetospirillum magneticum AMB-1	Pseudomonadota	GCF_000009985.1
Synechococcus elongatus PCC 6301	Cyanobacteriota	GCF_000010065.1
Brevibacillus brevis NBRC 100599	Bacillota	GCF_000010165.1
Finegoldia magna ATCC 29328	Bacillota	GCF_000010185.1
Kocuria rhizophila DC2201	Actinomycetota	GCF_000010285.1
Gemmatimonas aurantiaca T-27	Gemmatimonadota	GCF_000010305.1
Candidatus Vesicomyosocius okutanii HA	Pseudomonadota	GCF_000010405.1
Porphyromonas gingivalis ATCC 33277	Bacteroidota	GCF_000010505.1
Azorhizobium caulinodans ORS 571	Pseudomonadota	GCF_000010525.1
Streptomyces griseus subsp. griseus NBRC 13350	Actinomycetota	GCF_000010605.1
Microcystis aeruginosa NIES-843	Cyanobacteriota	GCF_000010625.1
Desulfovibrio magneticus RS-1	Thermodesulfobacteriota	GCF_000010665.1
Hydrogenobacter thermophilus TK-6	Aquificota	GCF_000010785.1
Deferribacter desulfuricans SSM1	Deferribacterota	GCF_000010985.1
Rothia mucilaginosa DY-18	Actinomycetota	GCF_000011025.1
Thermoplasma volcanium GSS1	Thermoplasmatota	GCF_000011185.1
Sulfolobus tokodaii str. 7	Thermoproteota	GCF_000011205.1
Oceanobacillus iheyensis HTE831	Bacillota	GCF_000011245.1
Thermosynechococcus elongatus BP-1	Cyanobacteriota	GCF_000011345.1
Bradyrhizobium diazoefficiens USDA 110	Pseudomonadota	GCF_000011365.1
Gloeobacter violaceus PCC 7421	Cyanobacteriota	GCF_000011385.1
Mycoplasma mycoides subsp. mycoides SC str. PG1	Mycoplasmatota	GCF_000011445.1
Burkholderia pseudomallei K96243	Pseudomonadota	GCF_000011545.1
Gluconobacter oxydans 621H	Pseudomonadota	GCF_000011685.1
Vibrio fischeri ES114	Pseudomonadota	GCF_000011805.1
Dehalococcoides mccartyi 195	Chloroflexota	GCF_000011905.1
Ruegeria pomeroyi DSS-3	Pseudomonadota	GCF_000011965.2
Borrelia turicatae 91E135	Spirochaetota	GCF_000012085.2
Pseudomonas syringae pv. syringae B728a	Pseudomonadota	GCF_000012245.1
Psychrobacter arcticus 273-4	Pseudomonadota	GCF_000012305.1

Colwellia psychrerythraea 34H	Pseudomonadota	GCF_000012325.1
Rickettsia bellii RML369-C	Pseudomonadota	GCF_000012385.1
Thermobifida fusca YX	Actinomycetota	GCF_000012405.1
Dechloromonas aromatica RCB	Pseudomonadota	GCF_000012425.1
Chlorobium luteolum DSM 273	Chlorobiota	GCF_000012485.1
Methanosphaera stadtmanae DSM 3091	Methanobacteriota	GCF_000012545.1
Ehrlichia canis str. Jake	Pseudomonadota	GCF_000012565.1
Chlorobium chlorochromatii CaD3	Chlorobiota	GCF_000012585.1
Hydrogenovibrio crunogenus XCL-2	Pseudomonadota	GCF_000012605.1
Myxococcus xanthus DK 1622	Myxococcota	GCF_000012685.1
Nitrobacter winogradskyi Nb-255	Pseudomonadota	GCF_000012725.1
Thiobacillus denitrificans ATCC 25259	Pseudomonadota	GCF_000012745.1
Nitrosococcus oceani ATCC 19707	Pseudomonadota	GCF_000012805.1
Parabacteroides distasonis ATCC 8503	Bacteroidota	GCF_000012845.1
Carboxydothermus hydrogenoformans Z-2901	Bacillota	GCF_000012865.1
Pelobacter carbinolicus DSM 2380	Thermodesulfobacteriota	GCF_000012885.1
Rhodobacter sphaeroides 2.4.1	Pseudomonadota	GCF_000012905.2
Sulfurimonas denitrificans DSM 1251	Campylobacterota	GCF_000012965.1
Hahella chejuensis KCTC 2396	Pseudomonadota	GCF_000012985.1
Erythrobacter litoralis HTCC2594	Pseudomonadota	GCF_000013005.1
Hyphomonas neptunium ATCC 15444	Pseudomonadota	GCF_000013025.1
Salinibacter ruber DSM 13855	Rhodothermota	GCF_000013045.1
Rhodospirillum rubrum ATCC 11170	Pseudomonadota	GCF_000013085.1
Moorella thermoacetica ATCC 39073	Bacillota	GCF_000013105.1
Anaplasma phagocytophilum str. HZ	Pseudomonadota	GCF_000013125.1
Neorickettsia sennetsu str. Miyayama	Pseudomonadota	GCF_000013165.1
Novosphingobium aromaticivorans DSM 12444	Pseudomonadota	GCF_000013325.1
Frankia casuarinae	Actinomycetota	GCF_000013345.1
Rhodopseudomonas palustris HaA2	Pseudomonadota	GCF_000013365.1
Syntrophus aciditrophicus SB	Thermodesulfobacteriota	GCF_000013405.1
Methanospirillum hungatei JF-1	Methanobacteriota	GCF_000013445.1
Rhodoferax ferrireducens T118	Pseudomonadota	GCF_000013605.1
Paraburkholderia xenovorans LB400	Pseudomonadota	GCF_000013645.1
Saccharophagus degradans 2-40	Pseudomonadota	GCF_000013665.1
Methanococcoides burtonii DSM 6242	Methanobacteriota	GCF_000013725.1
Shewanella denitrificans OS217	Pseudomonadota	GCF_000013765.1
Leptospira borgpetersenii serovar Hardjo-bovis str. L550	Spirochaetota	GCF_000013945.1
Sphingopyxis alaskensis RB2256	Pseudomonadota	GCF_000013985.1
Arcobacter butzleri RM4018	Campylobacterota	GCF_000014025.1
Roseobacter denitrificans OCh 114	Pseudomonadota	GCF_000014045.1
Cytophaga hutchinsonii ATCC 33406	Bacteroidota	GCF_000014145.1
Rubrobacter xylanophilus DSM 9941	Actinomycetota	GCF_000014185.1
Pseudoalteromonas atlantica T6c	Pseudomonadota	GCF_000014225.1
Trichodesmium erythraeum IMS101	Cyanobacteriota	GCF_000014265.1
Granulibacter bethesdensis CGDNIH1	Pseudomonadota	GCF_000014285.2
Oenococcus oeni PSU-1	Bacillota	GCF_000014385.1
Leuconostoc mesenteroides subsp. mesenteroides ATCC 8293	Bacillota	GCF_000014445.1
Pediococcus pentosaceus ATCC 25745	Bacillota	GCF_000014505.1
Lactobacillus paracasei ATCC 334	Bacillota	GCF_000014525.1
Rhodococcus jostii RHA1	Actinomycetota	GCF_000014565.1
Syntrophomonas wolfei subsp. wolfei str. Goettingen G311	Bacillota	GCF_000014725.1
Maricaulis maris MCS10	Pseudomonadota	GCF_000014745.1

Alkalilimnicola ehrlichii MLHE-1	Pseudomonadota	GCF_000014785.1
Aeromonas hydrophila subsp. hydrophila ATCC 7966	Pseudomonadota	GCF_000014805.1
Magnetococcus marinus MC-1	Pseudomonadota	GCF_000014865.1
Paenarthrobacter aurescens TC1	Actinomycetota	GCF_000014925.1
Syntrophobacter fumaroxidans MPOB	Thermodesulfobacteriota	GCF_000014965.1
Mycobacterium smegmatis str. MC2 155	Actinomycetota	GCF_000015005.1
Acidothermus cellulolyticus 11B	Actinomycetota	GCF_000015025.1
Hyperthermus butylicus DSM 5456	Thermoproteota	GCF_000015145.1
Psychromonas ingrahamii 37	Pseudomonadota	GCF_000015285.1
Dichelobacter nodosus VCS1703A	Pseudomonadota	GCF_000015345.1
Bartonella bacilliformis KC583	Pseudomonadota	GCF_000015445.1
Polaromonas naphthalenivorans CJ2	Pseudomonadota	GCF_000015505.1
Verminephrobacter eiseniae EF01-2	Pseudomonadota	GCF_000015565.1
Halorhodospira halophila SL1	Pseudomonadota	GCF_000015585.1
Methylibium petroleiphilum PM1	Pseudomonadota	GCF_000015725.1
Geobacillus thermodenitrificans NG80-2	Bacillota	GCF_000015745.1
Methanocorpusculum labreanum Z	Methanobacteriota	GCF_000015765.1
Methanoculleus marisnigri JR1	Methanobacteriota	GCF_000015825.1
Ruminiclostridium thermocellum ATCC 27405	Bacillota	GCF_000015865.1
Desulfotomaculum reducens MI-1	Bacillota	GCF_000016165.1
Agrobacterium vitis S4	Pseudomonadota	GCF_000016285.1
Polynucleobacter asymbioticus QLW-P1DMWA-1	Pseudomonadota	GCF_000016345.1
Salinispora tropica CNB-440	Actinomycetota	GCF_000016425.1
Methanobrevibacter smithii ATCC 35061	Methanobacteriota	GCF_000016525.1
Caldicellulosiruptor saccharolyticus DSM 8903	Bacillota	GCF_000016545.1
Metallosphaera sedula DSM 5348	Thermoproteota	GCF_000016605.1
Flavobacterium johnsoniae UW101	Bacteroidota	GCF_000016645.1
Sphingomonas wittichii RW1	Pseudomonadota	GCF_000016765.1
Thermosipho melanesiensis BI429	Thermotogota	GCF_000016905.1
Alkaliphilus metalliredigens QYMF	Bacillota	GCF_000016985.1
Actinobacillus succinogenes 130Z	Pseudomonadota	GCF_000017245.1
Phenylobacterium zucineum HLK1	Pseudomonadota	GCF_000017265.1
Kineococcus radiotolerans SRS30216 = ATCC BAA-149	Actinomycetota	GCF_000017305.1
Ochrobactrum anthropi ATCC 49188	Pseudomonadota	GCF_000017405.1
Fervidobacterium nodosum Rt17-B1	Thermotogota	GCF_000017545.1
Parvibaculum lavamentivorans DS-1	Pseudomonadota	GCF_000017565.1
Methanoregula boonei 6A8	Methanobacteriota	GCF_000017625.1
Xanthobacter autotrophicus Py2	Pseudomonadota	GCF_000017645.1
Vibrio campbellii ATCC BAA-1116	Pseudomonadota	GCF_000017705.1
Roseiflexus castenholzii DSM 13941	Chloroflexota	GCF_000017805.1
Pseudothermotoga lettingae TMO	Thermotogota	GCF_000017865.1
Ignicoccus hospitalis KIN4/I	Thermoproteota	GCF_000017945.1
Acaryochloris marina MBIC11017	Cyanobacteriota	GCF_000018105.1
Dinoroseobacter shibae DFL 12 = DSM 16493	Pseudomonadota	GCF_000018145.1
Caldivirga maquilingensis IC-167	Thermoproteota	GCF_000018305.1
Desulfococcus oleovorans Hxd3	Thermodesulfobacteriota	GCF_000018405.1
Sinorhizobium fredii NGR234	Pseudomonadota	GCF_000018545.1
Petrotoga mobilis SJ95	Thermotogota	GCF_000018605.1
Delftia acidovorans SPH-1	Pseudomonadota	GCF_000018665.1
Lachnoclostridium phytofermentans ISDg	Bacillota	GCF_000018685.1
Acholeplasma laidlawii PG-8A	Mycoplasmatota	GCF_000018785.1
Chloroflexus aurantiacus J-10-fl	Chloroflexota	GCF_000018865.1

Renibacterium salmoninarum ATCC 33209	Actinomycetota	GCF_000018885.1
Heliobacterium modesticaldum Ice1	Bacillota	GCF_000019165.1
Cellvibrio japonicus Ueda107	Pseudomonadota	GCF_000019225.1
Ureaplasma parvum serovar 3 str. ATCC 27815	Mycoplasmatota	GCF_000019345.1
Haemophilus somnus 2336	Pseudomonadota	GCF_000019405.1
Methylacidiphilum infernorum V4	Verrucomicrobiota	GCF_000019665.1
Methylobacterium radiotolerans JCM 2831	Pseudomonadota	GCF_000019725.1
Leptothrix cholodnii SP-6	Pseudomonadota	GCF_000019785.1
Beijerinckia indica subsp. indica ATCC 9039	Pseudomonadota	GCF_000019845.1
Exiguobacterium sibiricum 255-15	Bacillota	GCF_000019905.1
Opitutus terrae PB90-1	Verrucomicrobiota	GCF_000019965.1
Natranaerobius thermophilus JW/NM-WN-LF	Bacillota	GCF_000020005.1
Nostoc punctiforme PCC 73102	Cyanobacteriota	GCF_000020025.1
Elusimicrobium minutum Pei191	Elusimicrobiota	GCF_000020145.1
Akkermansia muciniphila ATCC BAA-835	Verrucomicrobiota	GCF_000020225.1
Anaplasma marginale str. Florida	Pseudomonadota	GCF_000020305.1
Desulfobacterium autotrophicum HRM2	Thermodesulfobacteriota	GCF_000020365.1
Halothermothrix orenii H 168	Bacillota	GCF_000020485.1
Chloroherpeton thalassium ATCC 35110	Chlorobiota	GCF_000020525.1
[Eubacterium] rectale ATCC 33656	Bacillota	GCF_000020605.1
Prosthecochloris aestuarii DSM 271	Chlorobiota	GCF_000020625.1
Desulfurococcus amylolyticus 1221n	Thermoproteota	GCF_000020905.1
Coprothermobacter proteolyticus DSM 5265	Coprothermobacterota	GCF_000020945.1
Dictyoglomus thermophilum H-6-12	Dictyoglomota	GCF_000020965.1
Thermodesulfovibrio yellowstonii DSM 11347	Nitrospirota	GCF_000020985.1
Laribacter hongkongensis HLHK9	Pseudomonadota	GCF_000021025.1
Gluconacetobacter diazotrophicus PA1 5	Pseudomonadota	GCF_000021325.1
Rhizobium leguminosarum bv. trifolii WSM2304	Pseudomonadota	GCF_000021345.1
Acidithiobacillus ferrooxidans ATCC 23270	Pseudomonadota	GCF_000021485.1
Sulfurihydrogenibium azorense Az-Fu1	Aquificota	GCF_000021545.1
Persephonella marina EX-H1	Aquificota	GCF_000021565.1
Thermomicrobium roseum DSM 5159	Thermomicrobiota	GCF_000021685.1
Nautilia profundicola AmH	Campylobacterota	GCF_000021725.1
Methylocella silvestris BL2	Pseudomonadota	GCF_000021745.1
Desulfitobacterium hafniense DCB-2	Bacillota	GCF_000021925.1
Methanosphaerula palustris E1-9c	Methanobacteriota	GCF_000021965.1
Thioalkalivibrio sulfidiphilus HL-EbGr7	Pseudomonadota	GCF_000021985.1
Pseudarthrobacter chlorophenolicus A6	Actinomycetota	GCF_000022025.1
Anaeromyxobacter dehalogenans 2CP-1	Myxococcota	GCF_000022145.1
Halorubrum lacusprofundi ATCC 49239	Methanobacteriota	GCF_000022205.1
Acidobacterium capsulatum ATCC 51196	Acidobacteriota	GCF_000022565.1
Teredinibacter turnerae T7901	Pseudomonadota	GCF_000023025.1
Tolumonas auensis DSM 9187	Pseudomonadota	GCF_000023065.1
Beutenbergia cavernae DSM 12333	Actinomycetota	GCF_000023105.1
Dyadobacter fermentans DSM 18053	Bacteroidota	GCF_000023125.1
Micrococcus luteus NCTC 2665	Actinomycetota	GCF_000023205.1
Desulfomicrobium baculatum DSM 4028	Thermodesulfobacteriota	GCF_000023225.1
Actinosynnema mirum DSM 43827	Actinomycetota	GCF_000023245.1
Acidimicrobium ferrooxidans DSM 10331	Actinomycetota	GCF_000023265.1
Capnocytophaga ochracea DSM 7271	Bacteroidota	GCF_000023285.1
Kosmotoga olearia TBF 19.5.1	Thermotogota	GCF_000023325.1
Brachybacterium faecium DSM 4810	Actinomycetota	GCF_000023405.1

Zunongwangia profunda SM-A87	Bacteroidota	GCF_000023465.1
Pectobacterium carotovorum subsp. carotovorum PC1	Pseudomonadota	GCF_000023605.1
Methylotenera mobilis JLW8	Pseudomonadota	GCF_000023705.1
Methylovorus glucosetrophus SIP3-4	Pseudomonadota	GCF_000023745.1
Hirschia baltica ATCC 49814	Pseudomonadota	GCF_000023785.1
Pedobacter heparinus DSM 2366	Bacteroidota	GCF_000023825.1
Cryptobacterium curtum DSM 15641	Actinomycetota	GCF_000023845.1
Saccharomonospora viridis DSM 43017	Actinomycetota	GCF_000023865.1
Slackia heliotrinireducens DSM 20476	Actinomycetota	GCF_000023885.1
Leptotrichia buccalis C-1013-b	Fusobacteriota	GCF_000023905.1
Kytococcus sedentarius DSM 20547	Actinomycetota	GCF_000023925.1
Halorhabdus utahensis DSM 12940	Methanobacteriota	GCF_000023945.1
Halomicrobium mukohataei DSM 12286	Methanobacteriota	GCF_000023965.1
Methanocaldococcus fervens AG86	Methanobacteriota	GCF_000023985.1
Chitinophaga pinensis DSM 2588	Bacteroidota	GCF_000024005.1
Catenulispora acidiphila DSM 44928	Actinomycetota	GCF_000024025.1
Jonesia denitrificans DSM 20603	Actinomycetota	GCF_000024065.1
Kangiella koreensis DSM 16069	Pseudomonadota	GCF_000024085.1
Anaerococcus prevotii DSM 20548	Bacillota	GCF_000024105.1
Robiginitalea biformata HTCC2501	Bacteroidota	GCF_000024125.1
Atopobium parvulum DSM 20469	Actinomycetota	GCF_000024225.1
Eggerthella lenta DSM 2243	Actinomycetota	GCF_000024265.1
Alicyclobacillus acidocaldarius subsp. acidocaldarius DSM 446	Bacillota	GCF_000024285.1
Desulfohalobium retbaense DSM 5692	Thermodesulfobacteriota	GCF_000024325.1
Kribbella flavida DSM 17836	Actinomycetota	GCF_000024345.1
Nakamurella multipartita DSM 44233	Actinomycetota	GCF_000024365.1
Thermomonospora curvata DSM 43183	Actinomycetota	GCF_000024385.1
Sebaldella termitidis ATCC 33386	Fusobacteriota	GCF_000024405.1
Meiothermus ruber DSM 1279	Deinococcota	GCF_000024425.1
Stackebrandtia nassauensis DSM 44728	Actinomycetota	GCF_000024545.1
Streptobacillus moniliformis DSM 12112	Fusobacteriota	GCF_000024565.1
Ammonifex degensii KC4	Bacillota	GCF_000024605.1
Halothiobacillus neapolitanus c2	Pseudomonadota	GCF_000024765.1
Gordonia bronchialis DSM 43247	Actinomycetota	GCF_000024785.1
Rhodothermus marinus DSM 4252	Rhodothermota	GCF_000024845.1
Streptosporangium roseum DSM 43021	Actinomycetota	GCF_000024865.1
Sulfurospirillum deleyianum DSM 6946	Campylobacterota	GCF_000024885.1
Thermanaerovibrio acidaminovorans DSM 6589	Synergistota	GCF_000024905.1
Sanguibacter keddieii DSM 10542	Actinomycetota	GCF_000024925.1
Veillonella parvula DSM 2008	Bacillota	GCF_000024945.1
Xylanimonas cellulosilytica DSM 15894	Actinomycetota	GCF_000024965.1
Sphaerobacter thermophilus DSM 20745	Thermomicrobiota	GCF_000024985.1
Thermobaculum terrenum ATCC BAA-798	Chloroflexota	GCF_000025005.1
Dickeya zeae Ech586	Pseudomonadota	GCF_000025065.1
Pirellula staleyi DSM 6068	Planctomycetota	GCF_000025185.1
Gardnerella vaginalis 409-05	Actinomycetota	GCF_000025205.1
Mageeibacillus indolicus UPII9-5	Bacillota	GCF_000025225.2
Conexibacter woesei DSM 14684	Actinomycetota	GCF_000025265.1
Acidaminococcus fermentans DSM 20731	Bacillota	GCF_000025305.1
Haloterrigena turkmenica DSM 5511	Methanobacteriota	GCF_000025325.1
Geodermatophilus obscurus DSM 43160	Actinomycetota	GCF_000025345.1
Pantoea ananatis LMG 20103	Pseudomonadota	GCF_000025405.2

Allochromatium vinosum DSM 180	Pseudomonadota	GCF_000025485.1
Ferroglobus placidus DSM 10642	Methanobacteriota	GCF_000025505.1
Enterobacter cloacae subsp. cloacae ATCC 13047	Pseudomonadota	GCF_000025565.1
Thermocrinis albus DSM 14484	Aquificota	GCF_000025605.1
Natrialba magadii ATCC 43099	Methanobacteriota	GCF_000025625.1
Thermoanaerobacter italicus Ab9	Bacillota	GCF_000025645.1
Aciduliprofundum boonei T469	Thermoplasmatota	GCF_000025665.1
Haloferax volcanii DS2	Methanobacteriota	GCF_000025685.1
Sideroxydans lithotrophicus ES-1	Pseudomonadota	GCF_000025705.1
Denitrovibrio acetiphilus DSM 12809	Deferribacterota	GCF_000025725.1
Methanohalophilus mahii DSM 5219	Methanobacteriota	GCF_000025865.1
Aminobacterium colombiense DSM 12261	Synergistota	GCF_000025885.1
Coraliomargarita akajimensis DSM 45221	Verrucomicrobiota	GCF_000025905.1
Prevotella ruminicola 23	Bacteroidota	GCF_000025925.1
Desulfotalea psychrophila LSv54	Thermodesulfobacteriota	GCF_000025945.1
Aromatoleum aromaticum EbN1	Pseudomonadota	GCF_000025965.1
Natronomonas pharaonis DSM 2160	Methanobacteriota	GCF_000026045.1
Herminiimonas arsenicoxydans	Pseudomonadota	GCF_000026125.1
Streptococcus equi subsp. zooepidemicus	Bacillota	GCF_000026605.1
Xenorhabdus bovienii SS-2004	Pseudomonadota	GCF_000027225.1
Chromohalobacter salexigens DSM 3043	Pseudomonadota	GCF_000055785.1
Lawsonia intracellularis PHE/MN1-00	Thermodesulfobacteriota	GCF_000055945.1
Gramella forsetii KT0803	Bacteroidota	GCF_000060345.1
Saccharopolyspora erythraea NRRL 2338	Actinomycetota	GCF_000062885.1
Methanocella arvoryzae MRE50	Methanobacteriota	GCF_000063445.1
Orientia tsutsugamushi str. Boryong	Pseudomonadota	GCF_000063545.1
Sorangium cellulosum So ce56	Myxococcota	GCF_000067165.1
Bordetella petrii	Pseudomonadota	GCF_000067205.1
Mycobacteroides abscessus	Actinomycetota	GCF_000069185.1
Clavibacter sepedonicus	Actinomycetota	GCF_000069225.1
Proteus mirabilis HI4320	Pseudomonadota	GCF_000069965.1
Stenotrophomonas maltophilia K279a	Pseudomonadota	GCF_000072485.1
Sphingobium japonicum UT26S	Pseudomonadota	GCF_000091125.1
Thermus thermophilus HB8	Deinococcota	GCF_000091545.1
Erwinia amylovora CFBP1430	Pseudomonadota	GCF_000091565.1
Agrobacterium fabrum str. C58	Pseudomonadota	GCF_000092025.1
Planctopirus limnophila DSM 3776	Planctomycetota	GCF_000092105.1
Desulfurivibrio alkaliphilus AHT 2	Thermodesulfobacteriota	GCF_000092205.1
Tsukamurella paurometabola DSM 20162	Actinomycetota	GCF_000092225.1
Moraxella catarrhalis BBH18	Pseudomonadota	GCF_000092265.1
Arcanobacterium haemolyticum DSM 20595	Actinomycetota	GCF_000092365.1
Syntrophothermus lipocalidus DSM 12680	Bacillota	GCF_000092405.1
Truepera radiovictrix DSM 17093	Deinococcota	GCF_000092425.1
Thermobispora bispora DSM 43833	Actinomycetota	GCF_000092645.1
Waddlia chondrophila WSU 86-1044	Chlamydiota	GCF_000092785.1
Segniliparus rotundus DSM 44985	Actinomycetota	GCF_000092825.1
Brachyspira murdochii DSM 12563	Spirochaetota	GCF_000092845.1
Cellulomonas flavigena DSM 20109	Actinomycetota	GCF_000092865.1
Kyrpidia tusciae DSM 2912	Bacillota	GCF_000092905.1
Starkeya novella DSM 506	Pseudomonadota	GCF_000092925.1
Nocardiopsis dassonvillei subsp. dassonvillei DSM 43111	Actinomycetota	GCF_000092985.1
[Bacillus] selenitireducens MLS10	Bacillota	GCF_000093085.1

Hyphomicrobium denitrificans ATCC 51888	Pseudomonadota	GCF_000143145.1
Dehalogenimonas lykanthroporepellens BL-DC-9	Chloroflexota	GCF_000143165.1
Sphingobacterium spiritivorum ATCC 33861	Bacteroidota	GCF_000143765.1
Olsenella uli DSM 7084	Actinomycetota	GCF_000143845.1
Desulfarculus baarsii DSM 2075	Thermodesulfobacteriota	GCF_000143965.1
Sediminispirochaeta smaragdinae DSM 11293	Spirochaetota	GCF_000143985.1
Brevundimonas subvibrioides ATCC 15264	Pseudomonadota	GCF_000144605.1
Thermosediminibacter oceani DSM 16646	Bacillota	GCF_000144645.1
Acetohalobium arabaticum DSM 5501	Bacillota	GCF_000144695.1
Acidilobus saccharovorans 345-15	Thermoproteota	GCF_000144915.1
Butyrivibrio proteoclasticus B316	Bacillota	GCF_000145035.1
Micromonospora aurantiaca ATCC 27029	Actinomycetota	GCF_000145235.1
Gallionella capsiferriformans ES-2	Pseudomonadota	GCF_000145255.1
Thermoanaerobacterium thermosaccharolyticum DSM 571	Bacillota	GCF_000145615.1
uncultured Termite group 1 bacterium phylotype Rs-D17	Elusimicrobiota	GCF_000146025.2
[Eubacterium] eligens ATCC 27750	Bacillota	GCF_000146185.1
Peptoniphilus duerdenii ATCC BAA-1640	Bacillota	GCF_000146345.1
Fibrobacter succinogenes subsp. succinogenes S85	Fibrobacterota	GCF_000146505.1
Peptostreptococcus stomatis DSM 17678	Bacillota	GCF_000147675.1
Mesotoga prima MesG1.Ag.4.2	Thermotogota	GCF_000147715.2
Methanolacinia petrolearia DSM 11571	Methanobacteriota	GCF_000147875.1
Ferrimonas balearica DSM 9799	Pseudomonadota	GCF_000148645.1
Listeria grayi DSM 20601	Bacillota	GCF_000148995.1
Oscillochloris trichoides DG-6	Chloroflexota	GCF_000152145.1
Ferroplasma acidarmanus fer1	Thermoplasmatota	GCF_000152265.2
Roseovarius nubinhibens ISM	Pseudomonadota	GCF_000152625.1
Pseudooceanicola batsensis HTCC2597	Pseudomonadota	GCF_000152725.1
Maritimibacter alkaliphilus HTCC2654	Pseudomonadota	GCF_000152805.1
Parvularcula bermudensis HTCC2503	Pseudomonadota	GCF_000152825.2
Leeuwenhoekiella blandensis MED217	Bacteroidota	GCF_000152985.1
Blastopirellula marina DSM 3645	Planctomycetota	GCF_000153105.1
Congregibacter litoralis KT71	Pseudomonadota	GCF_000153125.2
marine actinobacterium PHSC20C1	Actinomycetota	GCF_000153145.1
Reinekea blandensis MED297	Pseudomonadota	GCF_000153185.1
Nitrococcus mobilis Nb-231	Pseudomonadota	GCF_000153205.1
Polaribacter irgensii 23-P	Bacteroidota	GCF_000153225.1
Oceanicola granulosus HTCC2516	Pseudomonadota	GCF_000153305.1
Aurantimonas manganoxydans SI85-9A1	Pseudomonadota	GCF_000153465.1
Psychroflexus torquis ATCC 700755	Bacteroidota	GCF_000153485.2
Bermanella marisrubri	Pseudomonadota	GCF_000153565.1
Fulvimarina pelagi HTCC2506	Pseudomonadota	GCF_000153705.1
Salipiger bermudensis HTCC2601	Pseudomonadota	GCF_000153725.1
Mariprofundus ferrooxydans PV-1	Pseudomonadota	GCF_000153765.1
Blautia obeum ATCC 29174	Bacillota	GCF_000153905.1
Dorea longicatena DSM 13814	Bacillota	GCF_000154065.1
Actinomyces odontolyticus ATCC 17982	Actinomycetota	GCF_000154225.1
Absiella dolichum DSM 3991	Bacillota	GCF_000154285.1
Anaerostipes caccae DSM 14662	Bacillota	GCF_000154305.1
Coprococcus eutactus ATCC 27759	Bacillota	GCF_000154425.1
Intestinibacter bartlettii DSM 16795	Bacillota	GCF_000154445.1
Alistipes putredinis DSM 17216	Bacteroidota	GCF_000154465.1
Erysipelatoclostridium ramosum DSM 1402	Bacillota	GCF_000154485.1

Anaerotruncus colihominis DSM 17241	Bacillota	GCF_000154565.1
Hoeflea phototrophica DFL-43	Pseudomonadota	GCF_000154705.2
Kordia algicida OT-1	Bacteroidota	GCF_000154725.1
Anaerofustis stercorihominis DSM 17244	Bacillota	GCF_000154825.1
Ruminococcus lactaris ATCC 29176	Bacillota	GCF_000155205.1
Coleofasciculus chthonoplastes PCC 7420	Cyanobacteriota	GCF_000155555.1
Octadecabacter arcticus 238	Pseudomonadota	GCF_000155735.2
[Bacteroides] pectinophilus ATCC 43243	Bacillota	GCF_000155855.1
Mitsuokella multacida DSM 20544	Bacillota	GCF_000155955.1
Tyzzerella nexilis DSM 1787	Bacillota	GCF_000156035.2
[Clostridium] hiranonis DSM 13275	Bacillota	GCF_000156055.1
Collinsella intestinalis DSM 13280	Actinomycetota	GCF_000156175.1
Methylophaga thiooxydans DMS010	Pseudomonadota	GCF_000156355.1
Roseburia intestinalis L1-82	Bacillota	GCF_000156535.1
Holdemanella biformis DSM 3989	Bacillota	GCF_000156655.1
Subdoligranulum variabile DSM 15176	Bacillota	GCF_000157955.1
Labrenzia alexandrii DFL-11	Pseudomonadota	GCF_000158095.2
Luminiphilus syltensis NOR5-1B	Pseudomonadota	GCF_000158175.1
Oxalobacter formigenes HOxBLS	Pseudomonadota	GCF_000158475.2
Eikenella corrodens ATCC 23834	Pseudomonadota	GCF_000158615.1
Alloprevotella tannerae ATCC 51259	Bacteroidota	GCF_000159995.1
Catonella morbi ATCC 51271	Bacillota	GCF_000160035.2
Dialister invisus DSM 15470	Bacillota	GCF_000160055.1
Abiotrophia defectiva ATCC 49176	Bacillota	GCF_000160075.2
Hungatella hathewayi DSM 13479	Bacillota	GCF_000160095.1
Shuttleworthia satelles DSM 14600	Bacillota	GCF_000160115.1
Kingella oralis ATCC 51147	Pseudomonadota	GCF_000160435.1
Acetomicrobium hydrogeniformans ATCC BAA-1850	Synergistota	GCF_000160455.2
Weissella paramesenteroides ATCC 33313	Bacillota	GCF_000160575.1
Mobiluncus mulieris ATCC 35243	Actinomycetota	GCF_000160615.1
Oribacterium sinus F0268	Bacillota	GCF_000160635.1
Cardiobacterium hominis ATCC 15826	Pseudomonadota	GCF_000160655.1
Granulicatella adiacens ATCC 49175	Bacillota	GCF_000160675.1
Aeromicrobium marinum DSM 15272	Actinomycetota	GCF_000160775.2
Eubacterium saphenum ATCC 49989	Bacillota	GCF_000161975.1
Faecalibacterium prausnitzii A2-165	Bacillota	GCF_000162015.1
Simonsiella muelleri ATCC 29453	Pseudomonadota	GCF_000163775.2
Filifactor alocis ATCC 35896	Bacillota	GCF_000163895.2
Komagataeibacter hansenii ATCC 23769	Pseudomonadota	GCF_000164395.1
Roseomonas cervicalis ATCC 49957	Pseudomonadota	GCF_000164635.1
Paenibacillus polymyxa SC2	Bacillota	GCF_000164985.3
Halanaerobium praevalens DSM 2228	Bacillota	GCF_000165465.1
Stigmatella aurantiaca DW4/3-1	Myxococcota	GCF_000165485.1
Ilyobacter polytropus DSM 2926	Fusobacteriota	GCF_000165505.1
Rubinisphaera brasiliensis DSM 5305	Planctomycetota	GCF_000165715.2
Megasphaera micronuciformis F0359	Bacillota	GCF_000165735.1
Aminomonas paucivorans DSM 12260	Synergistota	GCF_000165795.1
Rhodomicrobium vannielii ATCC 17100	Pseudomonadota	GCF_000166055.1
Methanothermus fervidus DSM 2088	Methanobacteriota	GCF_000166095.1
Mucilaginibacter paludis DSM 18603	Bacteroidota	GCF_000166195.2
Algoriphagus machipongonensis	Bacteroidota	GCF_000166275.1
Leadbetterella byssophila DSM 17132	Bacteroidota	GCF_000166395.1

Crocosphaera watsonii WH 8501	Cyanobacteriota	GCF_000167195.1
Desulfuromonas acetoxidans DSM 684	Thermodesulfobacteriota	GCF_000167355.1
Rickettsiella grylli	Pseudomonadota	GCF_000168295.1
Thermosinus carboxydivorans Nor1	Bacillota	GCF_000169155.1
Microscilla marina ATCC 23134	Bacteroidota	GCF_000169175.1
Pseudoflavonifractor capillosus ATCC 29799	Bacillota	GCF_000169255.2
Sagittula stellata E-37	Pseudomonadota	GCF_000169415.1
Caminibacter mediatlanticus TB-2	Campylobacterota	GCF_000170735.1
Lentisphaera araneosa HTCC2155	Lentisphaerota	GCF_000170755.1
Plesiocystis pacifica SIR-1	Myxococcota	GCF_000170895.1
Geminisphaera colitermitum TAV2	Verrucomicrobiota	GCF_000171235.2
Gemmata obscuriglobus UQM 2246	Planctomycetota	GCF_000171775.1
Sulfitobacter indolifex HEL-45	Pseudomonadota	GCF_000172095.1
Verrucomicrobium spinosum DSM 4136 = JCM 18804	Verrucomicrobiota	GCF_000172155.1
Pedosphaera parvula Ellin514	Verrucomicrobiota	GCF_000172555.1
Alteromonas macleodii ATCC 27126	Pseudomonadota	GCF_000172635.2
Dethiosulfovibrio peptidovorans DSM 11002	Synergistota	GCF_000172975.1
Halogeometricum borinquense DSM 11551	Methanobacteriota	GCF_000172995.2
Chthoniobacter flavus Ellin428	Verrucomicrobiota	GCF_000173075.1
Catenibacterium mitsuokai DSM 15897	Bacillota	GCF_000173795.1
Marvinbryantia formatexigens DSM 14469	Bacillota	GCF_000173815.1
Gemella haemolysans ATCC 10379	Bacillota	GCF_000173915.1
Dethiobacter alkaliphilus AHT 1	Bacillota	GCF_000174415.1
Desulfonatronospira thiodismutans ASO3-1	Thermodesulfobacteriota	GCF_000174435.1
Asticcacaulis excentricus CB 48	Pseudomonadota	GCF_000175215.2
Zymomonas mobilis subsp. mobilis ATCC 10988	Pseudomonadota	GCF_000175255.2
Citromicrobium bathyomarinum JL354	Pseudomonadota	GCF_000176355.1
Acidovorax avenae subsp. avenae ATCC 19860	Pseudomonadota	GCF_000176855.2
Pyramidobacter piscolens W5455	Synergistota	GCF_000177335.1
Bulleidia extructa W1219	Bacillota	GCF_000177375.1
Desulfurispirillum indicum S5	Chrysiogenota	GCF_000177635.2
Ethanoligenens harbinense YUAN-3	Bacillota	GCF_000178115.2
Turicibacter sanguinis PC909	Bacillota	GCF_000178255.1
Brevibacterium mcbrellneri ATCC 49030	Actinomycetota	GCF_000178455.1
Methylosinus trichosporium OB3b	Pseudomonadota	GCF_000178815.2
Clostridium lentocellum DSM 5427	Bacillota	GCF_000178835.2
Ktedonobacter racemifer DSM 44963	Chloroflexota	GCF_000178855.1
Granulicella tundricola MP5ACTX9	Acidobacteriota	GCF_000178975.2
Methanothermococcus okinawensis IH1	Methanobacteriota	GCF_000179575.2
Hungateiclostridium cellulolyticum CD2	Bacillota	GCF_000179595.2
Terriglobus saanensis SP1PR4	Acidobacteriota	GCF_000179915.2
Plautia stali symbiont	Pseudomonadota	GCF_000180175.2
Gimesia maris DSM 8797	Planctomycetota	GCF_000181475.1
Paludibacter propionicigenes WB4	Bacteroidota	GCF_000183135.1
Calditerrivibrio nitroreducens DSM 19672	Deferribacterota	GCF_000183405.1
Marivirga tractuosa DSM 4126	Bacteroidota	GCF_000183425.1
Thermaerobacter subterraneus DSM 13965	Bacillota	GCF_000183545.2
Sulfuricurvum kujiense DSM 16994	Campylobacterota	GCF_000183725.1
Oceanithermus profundus DSM 14977	Deinococcota	GCF_000183745.1
Spirochaeta thermophila DSM 6578	Spirochaetota	GCF_000184345.1
Intrasporangium calvum DSM 43043	Actinomycetota	GCF_000184685.1
Variovorax paradoxus EPS	Pseudomonadota	GCF_000184745.1

Lachnoanaerobaculum saburreum DSM 3986	Bacillota	GCF_000185385.1
Pseudoramibacter alactolyticus ATCC 23263	Bacillota	GCF_000185505.1
Thermovibrio ammonificans HB-1	Aquificota	GCF_000185805.1
Mesorhizobium ciceri biovar biserrulae WSM1271	Pseudomonadota	GCF_000185905.1
Pseudoxanthomonas suwonensis 11-1	Pseudomonadota	GCF_000185965.1
Nitratifractor salsuginis DSM 16511	Campylobacterota	GCF_000186245.1
Cellulophaga algicola DSM 14237	Bacteroidota	GCF_000186265.1
Isosphaera pallida ATCC 43644	Planctomycetota	GCF_000186345.1
Lautropia mirabilis ATCC 51599	Pseudomonadota	GCF_000186425.1
Phascolarctobacterium succinatutens YIT 12067	Bacillota	GCF_000188175.1
Succinatimonas hippei YIT 12066	Pseudomonadota	GCF_000188195.1
Weeksella virosa DSM 16922	Bacteroidota	GCF_000189415.1
Vulcanisaeta moutnovskia 768-28	Thermoproteota	GCF_000190315.1
Sphaerochaeta globosa str. Buddy	Spirochaetota	GCF_000190435.1
Ornithinibacillus scapharcae TW25	Bacillota	GCF_000190475.1
Odoribacter splanchnicus DSM 20712	Bacteroidota	GCF_000190535.1
Syntrophobotulus glycolicus DSM 8271	Bacillota	GCF_000190635.1
Pseudopedobacter saltans DSM 12145	Bacteroidota	GCF_000190735.1
Methylobacter tundripaludum SV96	Pseudomonadota	GCF_000190755.2
Desulfurobacterium thermolithotrophum DSM 11699	Aquificota	GCF_000191045.1
Acinetobacter pittii PHEA-2	Pseudomonadota	GCF_000191145.1
Methanobacterium lacus	Methanobacteriota	GCF_000191585.1
Anaerophaga thermohalophila DSM 12881	Bacteroidota	GCF_000191885.1
Polymorphum gilvum SL003B-26A1	Pseudomonadota	GCF_000192745.1
Marinomonas mediterranea MMB-1	Pseudomonadota	GCF_000192865.1
Hippea maritima DSM 10411	Campylobacterota	GCF_000194135.1
Fluviicola taffensis DSM 16823	Bacteroidota	GCF_000194605.1
Desulfobacca acetoxidans DSM 11109	Thermodesulfobacteriota	GCF_000195295.1
Coriobacterium glomerans PW2	Actinomycetota	GCF_000195315.1
Marinithermus hydrothermalis DSM 14884	Deinococcota	GCF_000195335.1
Mycobacterium bovis AF2122/97	Actinomycetota	GCF_000195835.2
Cupriavidus metallidurans CH34	Pseudomonadota	GCF_000196015.1
Rhodopirellula baltica SH 1	Planctomycetota	GCF_000196115.1
Wolinella succinogenes DSM 1740	Campylobacterota	GCF_000196135.1
Bdellovibrio bacteriovorus HD100	Bdellovibrionota	GCF_000196175.1
Photobacterium profundum SS9	Pseudomonadota	GCF_000196255.1
Croceibacter atlanticus HTCC2559	Bacteroidota	GCF_000196315.1
Nitrosospira multiformis ATCC 25196	Pseudomonadota	GCF_000196355.1
Acetoanaerobium sticklandii	Bacillota	GCF_000196455.1
'Nostoc azollae' 0708	Cyanobacteriota	GCF_000196515.1
Methanohalobium evestigatum Z-7303	Methanobacteriota	GCF_000196655.1
Pseudonocardia dioxanivorans CB1190	Actinomycetota	GCF_000196675.1
Nitrospira defluvii	Nitrospirota	GCF_000196815.1
Amycolatopsis mediterranei U32	Actinomycetota	GCF_000196835.1
Halalkalicoccus jeotgali B3	Methanobacteriota	GCF_000196895.1
Glutamicibacter arilaitensis Re117	Actinomycetota	GCF_000197735.1
Anaerolinea thermophila UNI-1	Chloroflexota	GCF_000199675.1
Microbacterium testaceum StLB037	Actinomycetota	GCF_000202635.1
Acidiphilium multivorum AIU301	Pseudomonadota	GCF_000202835.1
Streptomyces coelicolor A3(2)	Actinomycetota	GCF_000203835.1
Paracoccus denitrificans PD1222	Pseudomonadota	GCF_000203895.1
Verrucosispora maris AB-18-032	Actinomycetota	GCF_000204155.1

[Propionibacterium] humerusii P08	Actinomycetota	GCF_000204235.1
Methanothrix soehngenii GP6	Methanobacteriota	GCF_000204415.1
Alicycliphilus denitrificans K601	Pseudomonadota	GCF_000204645.1
Parasutterella excrementihominis YIT 11859	Pseudomonadota	GCF_000205025.1
Paraprevotella xylaniphila YIT 11841	Bacteroidota	GCF_000205165.1
Selenomonas sputigena ATCC 35185	Bacillota	GCF_000208405.1
Gallibacterium anatis UMN179	Pseudomonadota	GCF_000209675.1
Gordonibacter pamelaeae 7-10-1-b	Actinomycetota	GCF_000210055.1
Arthrospira platensis NIES-39	Cyanobacteriota	GCF_000210375.1
Faecalitalea cylindroides T2-87	Bacillota	GCF_000210615.1
Halobacteriovorax marinus SJ	Bdellovibrionota	GCF_000210915.2
Hylemonella gracilis ATCC 19624	Pseudomonadota	GCF_000211835.1
Thermodesulfobium narugense DSM 14796	Thermodesulfobiota	GCF_000212395.1
Haliscomenobacter hydrossis DSM 1100	Bacteroidota	GCF_000212735.1
Acidianus hospitalis W1	Thermoproteota	GCF_000213215.1
Mahella australiensis 50-1 BON	Bacillota	GCF_000213255.1
Dysgonomonas gadei ATCC BAA-286	Bacteroidota	GCF_000213555.1
Centipeda periodontii DSM 2778	Bacillota	GCF_000213975.1
Methyloversatilis universalis FAM5	Pseudomonadota	GCF_000214035.1
Hoyosella subflava DQS3-9A1	Actinomycetota	GCF_000214175.1
Methanotorris igneus Kol 5	Methanobacteriota	GCF_000214415.1
Methylomonas methanica MC09	Pseudomonadota	GCF_000214665.1
Thiomicrospira cyclica ALM1	Pseudomonadota	GCF_000214825.1
Isoptericola variabilis 225	Actinomycetota	GCF_000215105.1
Ramlibacter tataouinensis TTB310	Pseudomonadota	GCF_000215705.1
Klebsiella aerogenes KCTC 2190	Pseudomonadota	GCF_000215745.1
Haloplasma contractile SSD-17B	Mycoplasmatota	GCF_000215935.2
Salinisphaera shabanensis E1L3A	Pseudomonadota	GCF_000215955.2
Thermodesulfobacterium geofontis OPF15	Thermodesulfobacteriota	GCF_000215975.1
Halopiger xanaduensis SH-6	Methanobacteriota	GCF_000217715.1
Thermodesulfatator indicus DSM 15286	Thermodesulfobacteriota	GCF_000217795.1
Methanosalsum zhilinae DSM 4017	Methanobacteriota	GCF_000217995.1
Oligotropha carboxidovorans OM5	Pseudomonadota	GCF_000218565.1
Flexistipes sinusarabici DSM 4947	Deferribacterota	GCF_000218625.1
Caldalkalibacillus thermarum TA2.A1	Bacillota	GCF_000218765.1
Acetonema longum DSM 6540	Bacillota	GCF_000219125.1
Pseudomonas stutzeri	Pseudomonadota	GCF_000219605.1
Thermophagus xiamenensis	Bacteroidota	GCF_000220155.1
Sporosarcina newyorkensis 2681	Bacillota	GCF_000220335.1
Ochrovirga pacifica	Bacteroidota	GCF_000220525.1
Croceivirga radicis	Bacteroidota	GCF_000220585.1
Cyclobacterium marinum DSM 745	Bacteroidota	GCF_000222485.1
Ketogulonicigenium vulgare WSH-001	Pseudomonadota	GCF_000223375.1
Pyrolobus fumarii 1A	Thermoproteota	GCF_000223395.1
Haloarcula hispanica ATCC 33960	Methanobacteriota	GCF_000223905.1
Thiocapsa marina 5811	Pseudomonadota	GCF_000223985.1
Marichromatium purpuratum 984	Pseudomonadota	GCF_000224005.2
Thiorhodococcus drewsii AZ1	Pseudomonadota	GCF_000224065.1
Muricauda ruestringensis DSM 13258	Bacteroidota	GCF_000224085.1
Bizionia argentinensis JUB59	Bacteroidota	GCF_000224335.1
halophilic archaeon DL31 Archaea.	Methanobacteriota	GCF_000224475.1
Serinicoccus profundi MCCC 1A05965	Actinomycetota	GCF_000224715.1

Lentibacillus jeotgali	Bacillota	GCF_000224785.1
Dietzia alimentaria 72	Actinomycetota	GCF_000226215.1
Chloracidobacterium thermophilum B	Acidobacteriota	GCF_000226295.1
Micavibrio aeruginosavorus ARL-13	Bdellovibrionota	GCF_000226315.1
Glaciecola nitratireducens FR1064	Pseudomonadota	GCF_000226565.1
Taylorella asinigenitalis MCE3	Pseudomonadota	GCF_000226625.1
Halobiforma lacisalsi AJ5	Methanobacteriota	GCF_000226975.2
Thermobacillus composti KWC4	Bacillota	GCF_000227705.2
Thiorhodospira sibirica ATCC 700588	Pseudomonadota	GCF_000227725.1
Thiocystis violascens DSM 198	Pseudomonadota	GCF_000227745.2
Pelagibacterium halotolerans B2	Pseudomonadota	GCF_000230555.1
Rhodanobacter denitrificans	Pseudomonadota	GCF_000230695.2
Natronobacterium gregoryi SP2	Methanobacteriota	GCF_000230715.2
Natrinema pellirubrum DSM 15624	Methanobacteriota	GCF_000230735.2
Leucobacter chromiiresistens JG 31	Actinomycetota	GCF_000231305.1
Desulfosporosinus meridiei DSM 13257	Bacillota	GCF_000231385.2
Commensalibacter intestini A911	Pseudomonadota	GCF_000231445.1
Desulfobacter postgatei 2ac9	Thermodesulfobacteriota	GCF_000233695.2
Thermovirga lienii DSM 17291	Synergistota	GCF_000233775.1
Johnsonella ignava ATCC 51276	Bacillota	GCF_000235445.1
Methanolinea tarda NOBI-1	Methanobacteriota	GCF_000235685.2
Halomonas boliviensis LC1	Pseudomonadota	GCF_000236035.1
Fluoribacter dumoffii NY 23	Pseudomonadota	GCF_000236165.1
Blattabacterium sp. (Cryptocercus punctulatus) str. Cpu	Bacteroidota	GCF_000236405.1
Dechlorosoma suillum PS	Pseudomonadota	GCF_000236665.1
Owenweeksia hongkongensis DSM 17368	Bacteroidota	GCF_000236705.1
Senegalimassilia anaerobia JC110	Actinomycetota	GCF_000236865.1
Pseudomonas fluorescens F113	Pseudomonadota	GCF_000237065.1
Simkania negevensis Z	Chlamydiota	GCF_000237205.1
Jonquetella anthropi DSM 22815	Synergistota	GCF_000237805.1
Haloquadratum walsbyi C23	Methanobacteriota	GCF_000237865.1
Peptoanaerobacter stomatis	Bacillota	GCF_000238095.2
Tannerella forsythia 92A2	Bacteroidota	GCF_000238215.1
Arthrobacter globiformis NBRC 12137	Actinomycetota	GCF_000238915.1
Anaeroglobus geminatus F0357	Bacillota	GCF_000239275.1
Patulibacter medicamentivorans	Actinomycetota	GCF_000240225.1
Stomatobaculum longum	Bacillota	GCF_000242235.1
Frateuria aurantia DSM 6220	Pseudomonadota	GCF_000242255.2
Singulisphaera acidiphila DSM 18658	Planctomycetota	GCF_000242455.2
Holophaga foetida DSM 6591	Acidobacteriota	GCF_000242615.2
Solitalea canadensis DSM 3403	Bacteroidota	GCF_000242635.2
Niabella soli DSM 19437	Bacteroidota	GCF_000243115.2
Gillisia limnaea DSM 15749	Bacteroidota	GCF_000243235.1
Methanoplanus limicola DSM 2279	Methanobacteriota	GCF_000243255.1
Myroides odoratus DSM 2801	Bacteroidota	GCF_000243275.1
Leptonema illini DSM 21528	Spirochaetota	GCF_000243335.1
Beggiatoa alba B18LD	Pseudomonadota	GCF_000245015.1
Helcococcus kunzii ATCC 51366	Bacillota	GCF_000245755.1
Megamonas funiformis YIT 11815	Bacillota	GCF_000245775.1
Facklamia languida CCUG 37842	Bacillota	GCF_000245795.1
Dolosigranulum pigrum ATCC 51524	Bacillota	GCF_000245815.1
Niastella koreensis GR20-10	Bacteroidota	GCF_000246855.1

Acetobacterium woodii DSM 1030	Bacillota	GCF_000247605.1
Mobilicoccus pelagius NBRC 104925	Actinomycetota	GCF_000247995.1
Atlantibacter hermannii NBRC 105704	Pseudomonadota	GCF_000248015.1
Saprospira grandis str. Lewin	Bacteroidota	GCF_000250635.1
Nocardia brasiliensis ATCC 700358	Actinomycetota	GCF_000250675.2
Sutterella parvirubra YIT 11816	Pseudomonadota	GCF_000250875.1
Riemerella anatipestifer ATCC 11845 = DSM 15868	Bacteroidota	GCF_000252855.1
Parachlamydia acanthamoebae UV-7	Chlamydiota	GCF_000253035.1
Marinitoga piezophila KA3	Thermotogota	GCF_000255135.1
Corallococcus coralloides DSM 2259	Myxococcota	GCF_000255295.1
Aquimarina agarilytica ZC1	Bacteroidota	GCF_000255455.1
Rahnella aquatilis HX2	Pseudomonadota	GCF_000255535.1
Schlesneria paludicola DSM 18645	Planctomycetota	GCF_000255655.1
Zavarzinella formosa DSM 19928	Planctomycetota	GCF_000255705.1
Diplorickettsia massiliensis 20B	Pseudomonadota	GCF_000257395.1
Ignavibacterium album JCM 16511	Ignavibacteriota	GCF_000258405.1
Marinilabilia salmonicolor JCM 21150	Bacteroidota	GCF_000259075.1
Providencia stuartii MRSN 2154	Pseudomonadota	GCF_000259175.1
Joostella marina DSM 19592	Bacteroidota	GCF_000260115.1
Imtechella halotolerans K1	Bacteroidota	GCF_000260835.1
Ensifer sojae CCBAU 05684	Pseudomonadota	GCF_000261485.1
Pasteurella bettyae CCUG 2042	Pseudomonadota	GCF_000262245.1
Shimwellia blattae DSM 4481 = NBRC 105725	Pseudomonadota	GCF_000262305.1
Microvirga lotononidis	Pseudomonadota	GCF_000262405.1
Tistrella mobilis KA081020-065	Pseudomonadota	GCF_000264455.2
Thermogladius calderae 1633	Thermoproteota	GCF_000264495.1
Edwardsiella anguillarum ET080813	Pseudomonadota	GCF_000264765.2
Nitritalea halalkaliphila LW7	Bacteroidota	GCF_000265075.1
Aequorivita sublithincola DSM 14238	Bacteroidota	GCF_000265385.1
Belliella baltica DSM 15883	Bacteroidota	GCF_000265405.1
Ornithobacterium rhinotracheale DSM 15997	Bacteroidota	GCF_000265465.1
Bernardetia litoralis DSM 6794	Bacteroidota	GCF_000265505.1
Turneriella parva DSM 21527	Spirochaetota	GCF_000266885.1
Desulfomonile tiedjei DSM 6799	Thermodesulfobacteriota	GCF_000266945.1
Scardovia wiggsiae F0424	Actinomycetota	GCF_000269605.1
Fictibacillus macauensis ZFHKF-1	Bacillota	GCF_000269865.1
Kitasatospora setae KM-6054	Actinomycetota	GCF_000269985.1
Erysipelothrix rhusiopathiae str. Fujisawa	Bacillota	GCF_000270085.1
Microlunatus phosphovorus NM-1	Actinomycetota	GCF_000270245.1
Hydrocarboniphaga effusa AP103	Pseudomonadota	GCF_000271305.1
Pelosinus fermentans JBW45	Bacillota	GCF_000271665.2
Alishewanella agri BL06	Pseudomonadota	GCF_000272005.1
Methanofollis liminatans DSM 4140	Methanobacteriota	GCF_000275865.1
Moritella dasanensis ArB 0140	Pseudomonadota	GCF_000276805.1
Pseudopropionibacterium propionicum F0230a	Actinomycetota	GCF_000277715.1
Melioribacter roseus P3M-2	Ignavibacteriota	GCF_000279145.1
Catenovulum agarivorans YM01	Pseudomonadota	GCF_000281085.1
Caldilinea aerophila DSM 14535 = NBRC 104270	Chloroflexota	GCF_000281175.1
Melaminivora alkalimesophila	Pseudomonadota	GCF_000282995.1
Arenitalea lutea	Bacteroidota	GCF_000283015.1
Methylocystis parvus OBBP	Pseudomonadota	GCF_000283235.1
Halogranum salarium B-1	Methanobacteriota	GCF_000283335.1

Oscillibacter valericigenes Sjm18-20	Bacillota	GCF_000283575.1
Tetragenococcus halophilus NBRC 12172	Bacillota	GCF_000283615.1
Blastococcus saxobsidens DD2	Actinomycetota	GCF_000284015.1
Phycisphaera mikurensis NBRC 102666	Planctomycetota	GCF_000284115.1
Rubrivivax gelatinosus IL144	Pseudomonadota	GCF_000284255.1
Actinoplanes missouriensis 431	Actinomycetota	GCF_000284295.1
Leptospirillum ferrooxidans C2-3	Nitrospirota	GCF_000284315.1
Caldisericum exile AZM16c01	Caldisericota	GCF_000284335.1
Pararhodospirillum photometricum DSM 122	Pseudomonadota	GCF_000284415.1
Halobacillus halophilus DSM 2266	Bacillota	GCF_000284515.1
Marinobacter hydrocarbonoclasticus ATCC 49840	Pseudomonadota	GCF_000284615.1
Ureibacillus thermosphaericus str. Thermo-BF	Bacillota	GCF_000284835.1
Kurthia massiliensis	Bacillota	GCF_000285555.1
Morganella morganii subsp. morganii KT	Pseudomonadota	GCF_000286435.2
secondary endosymbiont of Ctenarytaina eucalypti	Pseudomonadota	GCF_000287335.1
Phaeospirillum molischianum DSM 120	Pseudomonadota	GCF_000294655.1
Indibacter alkaliphilus LW1	Bacteroidota	GCF_000295935.2
Corynebacterium otitidis ATCC 51513	Actinomycetota	GCF_000296405.1
Barnesiella intestinihominis YIT 11860	Bacteroidota	GCF_000296465.1
Rheinheimera nanhaiensis E407-8	Pseudomonadota	GCF_000296695.1
Fibrisoma limi BUZ 3	Bacteroidota	GCF_000296815.2
Sulfuricella denitrificans skB26	Pseudomonadota	GCF_000297055.2
Caloramator australicus RC3	Bacillota	GCF_000297115.1
Nitrolancea hollandica Lb	Thermomicrobiota	GCF_000297255.1
Janibacter hoylei PVAS-1	Actinomycetota	GCF_000297495.1
Austwickia chelonae NBRC 105200	Actinomycetota	GCF_000298175.1
Kineosphaera limosa NBRC 100340	Actinomycetota	GCF_000298215.1
Cecembia lonarensis LW9	Bacteroidota	GCF_000298295.1
Leptospirillum ferriphilum ML-04	Nitrospirota	GCF_000299235.1
Gottschalkia acidurici 9a	Bacillota	GCF_000299355.1
Oceaniovalibus guishaninsula JLT2003	Pseudomonadota	GCF_000299575.1
Gallaecimonas xiamenensis 3-C-1	Pseudomonadota	GCF_000299915.1
Oceanibaculum indicum P24	Pseudomonadota	GCF_000299935.1
Thalassospira xiamenensis M-5 = DSM 17429	Pseudomonadota	GCF_000300235.2
Piscirickettsia salmonis LF-89 = ATCC VR-1361	Pseudomonadota	GCF_000300295.4
Nitratireductor pacificus pht-3B	Pseudomonadota	GCF_000300335.1
Kosakonia sacchari SP1	Pseudomonadota	GCF_000300455.3
Italian clover phyllody phytoplasma str. MA1	Mycoplasmatota	GCF_000300695.1
Gayadomonas joobiniege G7	Pseudomonadota	GCF_000300815.1
Galbibacter marinus	Bacteroidota	GCF_000300875.1
Bergeyella zoohelcum ATCC 43767	Bacteroidota	GCF_000301075.1
Pseudaminobacter salicylatoxidans KCT001	Pseudomonadota	GCF_000304395.1
Cardinium endosymbiont cEper1 of Encarsia pergandiella	Bacteroidota	GCF_000304455.1
Simiduia agarivorans SA1 = DSM 21679	Pseudomonadota	GCF_000305785.2
Thermacetogenium phaeum DSM 12270	Bacillota	GCF_000305935.1
Methanolobus psychrophilus R15	Methanobacteriota	GCF_000306725.1
Modestobacter marinus	Actinomycetota	GCF_000306785.1
Amphibacillus jilinensis Y1	Bacillota	GCF_000306965.1
Desulfobacula toluolica Tol2	Thermodesulfobacteriota	GCF_000307105.1
Pusillimonas noertemannii BS8	Pseudomonadota	GCF_000308195.1
Methanomassiliicoccus luminyensis B10	Thermoplasmatota	GCF_000308215.1
Levyella massiliensis	Bacillota	GCF_000308275.2

Afipia birgiae 34632	Pseudomonadota	GCF_000308295.2
Occidentia massiliensis	Pseudomonadota	GCF_000309075.1
Nodosilinea nodulosa PCC 7104	Cyanobacteriota	GCF_000309385.1
Oscillatoriales cyanobacterium JSC-12	Cyanobacteriota	GCF_000309945.1
Thauera aminoaromatica S2	Pseudomonadota	GCF_000310185.1
Enorma massiliensis phI	Actinomycetota	GCF_000311845.1
Kallipyga massiliensis ph2	Bacillota	GCF_000311985.1
Noviherbaspirillum massiliense JC206	Pseudomonadota	GCF_000312045.1
Timonella senegalensis JC301	Actinomycetota	GCF_000312125.1
Reyranella massiliensis 521	Pseudomonadota	GCF_000312425.1
Phocaeicola abscessus CCUG 55929	Bacteroidota	GCF_000312445.1
Fenollaria massiliensis	Bacillota	GCF_000312505.2
Dielma fastidiosa	Bacillota	GCF_000313565.1
Catellicoccus marimammalium M35/04/3	Bacillota	GCF_000313915.1
Spirulina subsalsa PCC 9445	Cyanobacteriota	GCF_000314005.1
Aliiglaciecola lipolytica E3	Pseudomonadota	GCF_000314975.1
Paraglaciecola arctica BSs20135	Pseudomonadota	GCF_000314995.1
Alloiococcus otitis ATCC 51267	Bacillota	GCF_000315445.1
Mastigocladopsis repens PCC 10914	Cyanobacteriota	GCF_000315565.1
Caenispirillum salinarum AK4	Pseudomonadota	GCF_000315795.1
Cyanobium gracile PCC 6307	Cyanobacteriota	GCF_000316515.1
Pleurocapsa sp. PCC 7327	Cyanobacteriota	GCF_000317025.1
Oscillatoria acuminata PCC 6304	Cyanobacteriota	GCF_000317105.1
Chroococcidiopsis thermalis PCC 7203	Cyanobacteriota	GCF_000317125.1
Chamaesiphon minutus PCC 6605	Cyanobacteriota	GCF_000317145.1
Fischerella thermalis PCC 7521	Cyanobacteriota	GCF_000317225.1
Chlorogloeopsis fritschii PCC 6912	Cyanobacteriota	GCF_000317285.1
Calothrix sp. PCC 6303	Cyanobacteriota	GCF_000317435.1
Crinalium epipsammum PCC 9333	Cyanobacteriota	GCF_000317495.1
Cylindrospermum stagnale PCC 7417	Cyanobacteriota	GCF_000317535.1
Stanieria cyanosphaera PCC 7437	Cyanobacteriota	GCF_000317575.1
Dactylococcopsis salina PCC 8305	Cyanobacteriota	GCF_000317615.1
Cyanobacterium aponinum PCC 10605	Cyanobacteriota	GCF_000317675.1
Anabaena cylindrica PCC 7122	Cyanobacteriota	GCF_000317695.1
Caldisphaera lagunensis DSM 15908	Thermoproteota	GCF_000317795.1
Carnobacterium maltaromaticum LMA28	Bacillota	GCF_000317975.2
Candidatus Kinetoplastibacterium blastocrithidii (ex Strigomonas culicis)	Pseudomonadota	GCF_000319245.1
Spiribacter salinus M19-40	Pseudomonadota	GCF_000319575.2
Moraxella macacae 0408225	Pseudomonadota	GCF_000320365.1
Echinicola vietnamensis DSM 17526	Bacteroidota	GCF_000325705.1
Liberibacter crescens BT-1	Pseudomonadota	GCF_000325745.1
Thioflavicoccus mobilis 8321	Pseudomonadota	GCF_000327045.1
Holdemania massiliensis AP2	Bacillota	GCF_000327285.1
Halobacteroides halobius DSM 5150	Bacillota	GCF_000328625.1
Methanomethylovorans hollandica DSM 15978	Methanobacteriota	GCF_000328665.1
Natronococcus occultus SP4	Methanobacteriota	GCF_000328685.1
Saccharothrix espanaensis DSM 44229	Actinomycetota	GCF_000328705.1
Tepidanaerobacter acetatoxydans Re1	Bacillota	GCF_000328765.2
Salinicoccus carnicancri Crm	Bacillota	GCF_000330705.1
Mariniradius saccharolyticus AK6	Bacteroidota	GCF_000330725.2
Fibrella aestuarina BUZ 2	Bacteroidota	GCF_000331105.1
Fulvivirga imtechensis AK7	Bacteroidota	GCF_000331535.1

Nonlabens dokdonensis DSW-6	Bacteroidota	GCF_000332115.1
Kamptonema formosum PCC 6407	Cyanobacteriota	GCF_000332155.1
Pseudanabaena biceps PCC 7429	Cyanobacteriota	GCF_000332215.1
Geminocystis herdmanii PCC 6308	Cyanobacteriota	GCF_000332235.1
cyanobacterium PCC 7702	Cyanobacteriota	GCF_000332255.1
Fortiea contorta PCC 7126	Cyanobacteriota	GCF_000332295.1
Prochlorothrix hollandica PCC 9006 = CALU 1027	Cyanobacteriota	GCF_000332315.1
Grimontia indica	Pseudomonadota	GCF_000333895.2
Halococcus agarilyticus	Methanobacteriota	GCF_000334895.1
Cystobacter fuscus DSM 2262	Myxococcota	GCF_000335475.2
Natronolimnobius innermongolicus JCM 12255	Methanobacteriota	GCF_000337215.1
Halosimplex carlsbadense 2-9-1	Methanobacteriota	GCF_000337455.1
Halovivax asiaticus JCM 14624	Methanobacteriota	GCF_000337515.1
Natronorubrum bangense JCM 10635	Methanobacteriota	GCF_000337715.1
Eggerthia catenaformis OT 569 = DSM 20559	Bacillota	GCF_000340375.1
Nodularia spumigena CCY9414	Cyanobacteriota	GCF_000340565.2
Desulfocapsa sulfexigens DSM 10523	Thermodesulfobacteriota	GCF_000341395.1
Nitrospina gracilis 3/211	Nitrospinota	GCF_000341545.2
Methylomicrobium buryatense 5G	Pseudomonadota	GCF_000341735.1
Pacificimonas flava	Pseudomonadota	GCF_000342165.1
Scytonema hofmannii PCC 7110	Cyanobacteriota	GCF_000346485.2
Nafulsella turpanensis ZLM-10	Bacteroidota	GCF_000346615.1
Bibersteinia trehalosi USDA-ARS-USMARC-192	Pseudomonadota	GCF_000347595.1
Xanthomarina gelatinilytica	Bacteroidota	GCF_000348685.1
Bhargavaea cecembensis DSE10	Bacillota	GCF_000348905.1
Cesiribacter andamanensis AMV16	Bacteroidota	GCF_000348925.1
Paeniglutamicibacter gangotriensis Lz1y	Actinomycetota	GCF_000348945.1
Curtobacterium flaccumfaciens UCD-AKU	Actinomycetota	GCF_000349565.1
Richelia intracellularis HH01	Cyanobacteriota	GCF_000350105.1
Ilumatobacter nonamiensis YM16-303	Actinomycetota	GCF_000350145.1
Thermoplasmatales archaeon BRNA1	Thermoplasmatota	GCF_000350305.1
Thalassolituus oleivorans MIL-1	Pseudomonadota	GCF_000355675.1
Gracilibacillus halophilus YIM-C55.5	Bacillota	GCF_000359605.1
Peanut witches'-broom phytoplasma NTU2011	Mycoplasmatota	GCF_000364425.1
Pseudomonas thermotolerans DSM 14292	Pseudomonadota	GCF_000364625.1
Robiginitomaculum antarcticum DSM 21748	Pseudomonadota	GCF_000365025.1
Tetrasphaera elongata Lp2	Actinomycetota	GCF_000367525.1
Acinetobacter bohemicus ANC 3994	Pseudomonadota	GCF_000367925.1
Actinopolyspora halophila DSM 43834	Actinomycetota	GCF_000371785.1
Thiolinea disciformis DSM 14473	Pseudomonadota	GCF_000371925.1
Oligella urethralis DSM 7531	Pseudomonadota	GCF_000372065.1
Methylosarcina fibrata AML-C10	Pseudomonadota	GCF_000372865.1
Lewinella persica DSM 23188	Bacteroidota	GCF_000373105.1
Pontibacter roseus DSM 17521	Bacteroidota	GCF_000373265.1
Dasania marina DSM 21967	Pseudomonadota	GCF_000373485.1
Actinopolymorpha alba DSM 45243	Actinomycetota	GCF_000373925.1
Uliginosibacterium gangwonense DSM 18521	Pseudomonadota	GCF_000373965.1
Kiloniella laminariae DSM 19542	Pseudomonadota	GCF_000374005.1
Segetibacter koreensis DSM 18137	Bacteroidota	GCF_000374045.1
Spirosoma luteum DSM 19990	Bacteroidota	GCF_000374065.1
Neomegalonema perideroedes DSM 15528	Pseudomonadota	GCF_000374145.1
Actinomadura flavalba DSM 45200	Actinomycetota	GCF_000374305.1

Allofustis seminis DSM 15817	Bacillota	GCF_000374325.1
Actinokineospora enzanensis DSM 44649	Actinomycetota	GCF_000374445.1
Ahrensia kielensis DSM 5890	Pseudomonadota	GCF_000374465.1
Algicola sagamiensis DSM 14643	Pseudomonadota	GCF_000374485.1
Amorphus coralli DSM 19760	Pseudomonadota	GCF_000374525.1
Brachymonas chironomi DSM 19884	Pseudomonadota	GCF_000374625.1
Arhodomonas aquaeolei DSM 8974	Pseudomonadota	GCF_000374645.1
Chitiniphilus shinanonensis DSM 23277	Pseudomonadota	GCF_000374805.1
Gracilimonas tropica DSM 19535	Balneolota	GCF_000375425.1
Balneola vulgaris DSM 17893	Balneolota	GCF_000375465.1
Kordiimonas gwangyangensis DSM 19435 = JCM 12864	Pseudomonadota	GCF_000375545.1
Effusibacillus pohliae DSM 22757	Bacillota	GCF_000376225.1
Acaricomes phytoseiuli DSM 14247	Actinomycetota	GCF_000376245.1
Desulfurispora thermophila DSM 16022	Bacillota	GCF_000376385.1
Haladaptatus paucihalophilus DX253	Methanobacteriota	GCF_000376445.1
Mannheimia haemolytica M42548	Pseudomonadota	GCF_000376645.1
Henriciella marina DSM 19595	Pseudomonadota	GCF_000376805.1
Demetria terragena DSM 11295	Actinomycetota	GCF_000376825.1
Alloscardovia criceti DSM 17774	Actinomycetota	GCF_000376885.1
Leeia oryzae DSM 17879	Pseudomonadota	GCF_000376945.1
Salsuginibacillus kocurii DSM 18087	Bacillota	GCF_000377705.1
Gilvimarinus chinensis DSM 19667	Pseudomonadota	GCF_000377745.1
Sporolactobacillus vineae DSM 21990 = SL153	Bacillota	GCF_000377985.1
Marinobacterium rhizophilum DSM 18822	Pseudomonadota	GCF_000378045.1
Rubritalea marina DSM 17716	Verrucomicrobiota	GCF_000378105.1
Rudaea cellulosilytica DSM 22992	Pseudomonadota	GCF_000378125.1
Saccharibacillus kuerlensis DSM 22868	Bacillota	GCF_000378145.1
Saccharibacter floricola DSM 15669	Pseudomonadota	GCF_000378165.1
Salinimonas chungwhensis DSM 16280	Pseudomonadota	GCF_000378185.1
Methylophilus methylotrophus DSM 46235 = ATCC 53528	Pseudomonadota	GCF_000378225.1
Azoarcus toluclasticus ATCC 700605	Pseudomonadota	GCF_000378245.1
Succinimonas amylolytica DSM 2873	Pseudomonadota	GCF_000378405.1
Cohnella laeviribosi DSM 21336	Bacillota	GCF_000378425.1
Elioraea tepidiphila DSM 17972	Pseudomonadota	GCF_000378465.1
Orenia marismortui DSM 5156	Bacillota	GCF_000379025.1
Fangia hongkongensis FSC776 = DSM 21703	Pseudomonadota	GCF_000379445.1
Sciscionella marina DSM 45152	Actinomycetota	GCF_000379465.1
Wenxinia marina DSM 24838	Pseudomonadota	GCF_000379485.1
Lamprocystis purpurea DSM 4197	Pseudomonadota	GCF_000379525.1
Actinomycetospora chiangmaiensis DSM 45062	Actinomycetota	GCF_000379625.1
Butyricimonas synergistica DSM 23225	Bacteroidota	GCF_000379665.1
Catelliglobosispora koreensis DSM 44566	Actinomycetota	GCF_000379685.1
Flexithrix dorotheae DSM 6795	Bacteroidota	GCF_000379765.1
Longispora albida DSM 44784	Actinomycetota	GCF_000379825.1
Nonomuraea coxensis DSM 45129	Actinomycetota	GCF_000379885.1
Azotobacter vinelandii CA	Pseudomonadota	GCF_000380335.1
Kaistia granuli DSM 23481	Pseudomonadota	GCF_000380505.1
Microbulbifer variabilis ATCC 700307	Pseudomonadota	GCF_000380565.1
Smaragdicoccus niigatensis DSM 44881 = NBRC 103563	Actinomycetota	GCF_000380645.1
Propionispira raffinosivorans DSM 20765	Bacillota	GCF_000381065.1
Caldimonas manganoxidans ATCC BAA-369	Pseudomonadota	GCF_000381125.1
Curvibacter lanceolatus ATCC 14669	Pseudomonadota	GCF_000381265.1

Rhodonellum psychrophilum GCM71 = DSM 17998	Bacteroidota	GCF_000381545.1
[Zimmermannella] faecalis ATCC 13722	Actinomycetota	GCF_000381765.1
Amphritea japonica ATCC BAA-1530	Pseudomonadota	GCF_000381785.1
Oceanimonas smirnovii ATCC BAA-899	Pseudomonadota	GCF_000381965.1
Novispirillum itersonii subsp. itersonii ATCC 12639	Pseudomonadota	GCF_000381985.1
Eudoraea adriatica DSM 19308	Bacteroidota	GCF_000382125.1
Hymenobacter aerophilus DSM 13606	Bacteroidota	GCF_000382225.1
Kushneria aurantia DSM 21353	Pseudomonadota	GCF_000382245.1
Vitreoscilla stercoraria DSM 513	Pseudomonadota	GCF_000382305.1
Woodsholea maritima DSM 17123	Pseudomonadota	GCF_000382325.1
Massilia niastensis DSM 21313	Pseudomonadota	GCF_000382345.1
Empedobacter brevis NBRC 14943 = ATCC 43319	Bacteroidota	GCF_000382425.1
Aureimonas ureilytica DSM 18598 = NBRC 106430	Pseudomonadota	GCF_000382705.1
Caldibacillus debilis DSM 16016	Bacillota	GCF_000383875.1
Duganella zoogloeoides ATCC 25935	Pseudomonadota	GCF_000383895.1
Rudanella lutea DSM 19387	Bacteroidota	GCF_000383955.1
Methylovulum miyakonense HT12	Pseudomonadota	GCF_000384075.1
Sporichthya polymorpha DSM 43042	Actinomycetota	GCF_000384115.1
Succinispira mobilis DSM 6222	Bacillota	GCF_000384135.1
Allobaculum stercoricanis DSM 13633	Bacillota	GCF_000384195.1
Geopsychrobacter electrodiphilus DSM 16401	Thermodesulfobacteriota	GCF_000384395.1
Cycloclasticus pugetii PS-1	Pseudomonadota	GCF_000384415.1
Promicromonospora sukumoe 327MFSha3.1	Actinomycetota	GCF_000385135.1
Methyloferula stellata AR4	Pseudomonadota	GCF_000385335.1
Caldisalinibacter kiritimatiensis	Bacillota	GCF_000387765.1
Holospora undulata HU1	Pseudomonadota	GCF_000388175.3
Lunatimonas lonarensis	Bacteroidota	GCF_000390185.1
Butyricicoccus pullicaecorum 1.2	Bacillota	GCF_000398925.1
Spiroplasma chrysopicola DF-1	Mycoplasmatota	GCF_000400935.1
Arcticibacter svalbardensis MN12-7	Bacteroidota	GCF_000403135.1
Enterorhabdus caecimuris B7	Actinomycetota	GCF_000403355.2
Euryhalocaulis caribicus	Pseudomonadota	GCF_000412185.1
Agarivorans albus MKT 106	Pseudomonadota	GCF_000414175.1
Gryllotalpicola ginsengisoli DSM 22003	Actinomycetota	GCF_000419445.1
Leucothrix mucor DSM 2157	Pseudomonadota	GCF_000419525.1
Salisaeta longa DSM 21114	Rhodothermota	GCF_000419585.1
Perlucidibaca piscinae DSM 21586	Pseudomonadota	GCF_000420045.1
Varibaculum cambriense DSM 15806	Actinomycetota	GCF_000420065.1
Desulfospira joergensenii DSM 10085	Thermodesulfobacteriota	GCF_000420085.1
Oceanicaulis alexandrii DSM 11625	Pseudomonadota	GCF_000420265.1
Spongiibacter tropicus DSM 19543	Pseudomonadota	GCF_000420325.1
Faecalicoccus pleomorphus DSM 20574	Bacillota	GCF_000420345.1
Actinotignum urinale DSM 15805	Actinomycetota	GCF_000420445.1
Aquaspirillum serpens DSM 68	Pseudomonadota	GCF_000420525.1
Arenimonas oryziterrae DSM 21050 = YC6267	Pseudomonadota	GCF_000420545.1
Fodinicurvata sediminis DSM 21159	Pseudomonadota	GCF_000420625.1
Nevskia ramosa DSM 11499	Pseudomonadota	GCF_000420645.1
Ponticaulis koreensis DSM 19734	Pseudomonadota	GCF_000420665.1
Marinococcus halotolerans DSM 16375	Bacillota	GCF_000420725.1
Pseudorhodobacter ferrugineus DSM 5888	Pseudomonadota	GCF_000420745.1
Sandarakinorhabdus limnophila DSM 17366	Pseudomonadota	GCF_000420765.1
Yaniella halotolerans DSM 15476	Actinomycetota	GCF_000420805.1

Glaciibacter superstes DSM 21135	Actinomycetota	GCF_000421145.1
Ornithinimicrobium pekingense DSM 21552	Actinomycetota	GCF_000421185.1
Ruania albidiflava DSM 18029	Actinomycetota	GCF_000421225.1
Desulfotignum balticum DSM 7044	Thermodesulfobacteriota	GCF_000421285.1
Methylohalobius crimeensis 10Ki	Pseudomonadota	GCF_000421465.1
Aestuariimicrobium kwangyangense DSM 21549	Actinomycetota	GCF_000421525.1
Agromyces italicus DSM 16388	Actinomycetota	GCF_000421545.1
Bavariicoccus seileri DSM 19936	Bacillota	GCF_000421665.1
Nesterenkonia alba DSM 19423	Actinomycetota	GCF_000421745.1
Humibacter albus DSM 18994	Actinomycetota	GCF_000421825.1
Serratia liquefaciens ATCC 27592	Pseudomonadota	GCF_000422085.1
Chryseobacterium palustre DSM 21579	Bacteroidota	GCF_000422265.1
Desulfatiglans anilini DSM 4660	Thermodesulfobacteriota	GCF_000422285.1
Luteimonas mephitis DSM 12574	Pseudomonadota	GCF_000422305.1
Marinospirillum minutulum DSM 6287	Pseudomonadota	GCF_000422325.1
Mesoflavibacter zeaxanthinifaciens DSM 18436	Bacteroidota	GCF_000422365.1
Oceanospirillum beijerinckii DSM 7166	Pseudomonadota	GCF_000422425.1
Halodesulfovibrio aestuarii DSM 10141	Thermodesulfobacteriota	GCF_000422525.1
Neptunomonas japonica DSM 18939	Pseudomonadota	GCF_000422765.1
Nesiotobacter exalbescens DSM 16456	Pseudomonadota	GCF_000422785.1
Nocardioides halotolerans DSM 19273	Actinomycetota	GCF_000422805.1
Oceanobacter kriegii DSM 6294	Pseudomonadota	GCF_000422845.1
Ottowia thiooxydans DSM 14619	Pseudomonadota	GCF_000422885.1
Paludibacterium yongneupense DSM 18731	Pseudomonadota	GCF_000422925.1
Pleomorphomonas oryzae DSM 16300	Pseudomonadota	GCF_000422965.1
Porphyrobacter cryptus DSM 12079	Pseudomonadota	GCF_000422985.1
Gelidibacter mesophilus DSM 14095	Bacteroidota	GCF_000423005.1
Granulicoccus phenolivorans DSM 17626	Actinomycetota	GCF_000423085.1
Halalkalibacillus halophilus DSM 18494	Bacillota	GCF_000423105.1
Haliea salexigens DSM 19537	Pseudomonadota	GCF_000423125.1
Inquilinus limosus DSM 16000	Pseudomonadota	GCF_000423185.1
Lysobacter defluvii IMMIB APB-9 = DSM 18482	Pseudomonadota	GCF_000423325.1
Marinimicrobium agarilyticum DSM 16975	Pseudomonadota	GCF_000423345.1
Maritalea myrionectae DSM 19524	Pseudomonadota	GCF_000423365.1
Mesonia mobilis DSM 19841	Bacteroidota	GCF_000423405.1
Acidipropionibacterium thoenii DSM 20276	Actinomycetota	GCF_000423445.1
Propionicicella superfundia DSM 22317	Actinomycetota	GCF_000423465.1
Propionimicrobium lymphophilum DSM 4903	Actinomycetota	GCF_000423485.1
Proteocatella sphenisci DSM 23131	Bacillota	GCF_000423525.1
Runella zeae DSM 19591	Bacteroidota	GCF_000423565.1
Salinimicrobium xinjiangense DSM 19287	Bacteroidota	GCF_000423585.1
Saccharospirillum impatiens DSM 12546	Pseudomonadota	GCF_000423605.1
Sediminimonas qiaohouensis DSM 21189	Pseudomonadota	GCF_000423645.1
Solirubrobacter soli DSM 22325	Actinomycetota	GCF_000423665.1
Stappia stellulata DSM 5886	Pseudomonadota	GCF_000423705.1
Thalassobaculum salexigens DSM 19539	Pseudomonadota	GCF_000423805.1
Thermithiobacillus tepidarius DSM 3134	Pseudomonadota	GCF_000423825.1
Thermomonas fusca DSM 15424	Pseudomonadota	GCF_000423885.1
Lachnospira multipara ATCC 19207	Bacillota	GCF_000424105.1
Arenibacter latericius DSM 15913	Bacteroidota	GCF_000424985.1
Solobacterium moorei DSM 22971	Bacillota	GCF_000425005.1
Agrococcus lahaulensis DSM 17612	Actinomycetota	GCF_000425105.1

Pontibacillus halophilus JSM 076056 = DSM 19796	Bacillota	GCF_000425205.1
Psychroserpens burtonensis DSM 12212	Bacteroidota	GCF_000425305.1
Rubritepida flocculans DSM 14296	Pseudomonadota	GCF_000425365.1
Pseudoduganella violaceinigra DSM 15887	Pseudomonadota	GCF_000425385.1
Tepidiphilus margaritifer DSM 15129	Pseudomonadota	GCF_000425565.1
Terrimonas ferruginea DSM 30193	Bacteroidota	GCF_000425585.1
Gaetbulibacter saemankumensis DSM 17032	Bacteroidota	GCF_000425645.1
Gulosibacter molinativorax DSM 13485	Actinomycetota	GCF_000425685.1
Jeotgalicoccus marinus DSM 19772	Bacillota	GCF_000425825.1
Lacticigenium naphtae DSM 19658	Bacillota	GCF_000425865.1
Desulforegula conservatrix Mb1Pa	Thermodesulfobacteriota	GCF_000426225.1
Anaerovorax odorimutans DSM 5092	Bacillota	GCF_000426305.1
Arsenicicoccus bolidensis DSM 15745	Actinomycetota	GCF_000426385.1
Nisaea denitrificans DSM 18348	Pseudomonadota	GCF_000426505.1
Psychrilyobacter atlanticus DSM 19335	Fusobacteriota	GCF_000426625.1
Solimonas flava DSM 18980	Pseudomonadota	GCF_000426685.1
Sporocytophaga myxococcoides DSM 11118	Bacteroidota	GCF_000426725.1
Thermonema rossianum DSM 10300	Bacteroidota	GCF_000426825.1
Dolichospermum circinale AWQC310F	Cyanobacteriota	GCF_000426925.1
Chthonomonas calidirosea T49	Armatimonadota	GCF_000427095.1
Winogradskyella psychrotolerans RS-3	Bacteroidota	GCF_000427335.1
Rikenella microfusus DSM 15922	Bacteroidota	GCF_000427365.1
Methylocaldum szegediense O-12	Pseudomonadota	GCF_000427385.1
Flectobacillus major DSM 103	Bacteroidota	GCF_000427405.1
Caldanaerobius polysaccharolyticus DSM 13641	Bacillota	GCF_000427425.1
Methylocapsa acidiphila B2	Pseudomonadota	GCF_000427445.1
Geminicoccus roseus DSM 18922	Pseudomonadota	GCF_000427665.1
Haloplanus natans DSM 17983	Methanobacteriota	GCF_000427685.1
Budvicia aquatica DSM 5075 = ATCC 35567	Pseudomonadota	GCF_000427805.1
Thermocrispum municipale DSM 44069	Actinomycetota	GCF_000427825.1
Glycomyces tenuis DSM 44171	Actinomycetota	GCF_000427885.1
Olivibacter sitiensis DSM 17696	Bacteroidota	GCF_000427965.1
Sedimenticola selenatireducens DSM 17993	Pseudomonadota	GCF_000428045.1
Shimazuella kribbensis DSM 45090	Bacillota	GCF_000428065.1
Chitinilyticum litopenaei DSM 21440	Pseudomonadota	GCF_000428145.1
Chitinimonas koreensis DSM 17726	Pseudomonadota	GCF_000428465.1
Zooshikella ganghwensis DSM 15267	Pseudomonadota	GCF_000428585.1
Conchiformibius kuhniae DSM 17694	Pseudomonadota	GCF_000428785.1
Eremococcus coleocola DSM 15696	Bacillota	GCF_000428865.1
Geothrix fermentans DSM 14018	Acidobacteriota	GCF_000428885.1
Hamadaea tsunoensis DSM 44101	Actinomycetota	GCF_000428945.1
Salinarimonas rosea DSM 21201	Pseudomonadota	GCF_000429045.1
Silanimonas lenta DSM 16282	Pseudomonadota	GCF_000429065.1
Aestuariibacter salexigens DSM 15300	Pseudomonadota	GCF_000429145.1
Acetobacter nitrogenifigens DSM 23921 = LMG 23498	Pseudomonadota	GCF_000429165.1
Desulfovirgula thermocuniculi DSM 16036	Bacillota	GCF_000429345.1
Pseudodonghicola xiamenensis DSM 18339	Pseudomonadota	GCF_000429365.1
Aliagarivorans marinus DSM 23064	Pseudomonadota	GCF_000429485.1
Atopococcus tabaci DSM 17538	Bacillota	GCF_000429585.1
Azonexus hydrophilus DSM 23864	Pseudomonadota	GCF_000429605.1
Azospirillum halopraeferens DSM 3675	Pseudomonadota	GCF_000429625.1
Niveispirillum irakense DSM 11586	Pseudomonadota	GCF_000429645.1

Azovibrio restrictus DSM 23866	Pseudomonadota	GCF_000429665.1
Gemmobacter nectariphilus DSM 15620	Pseudomonadota	GCF_000429765.1
Chitinibacter tainanensis DSM 15459	Pseudomonadota	GCF_000429785.1
Comamonas composti DSM 21721	Pseudomonadota	GCF_000429845.1
Cucumibacter marinus DSM 18995	Pseudomonadota	GCF_000429865.1
Dermatophilus congolensis DSM 44180 = NBRC 105199	Actinomycetota	GCF_000429885.1
Desulfatibacillum aliphaticivorans DSM 15576	Thermodesulfobacteriota	GCF_000429905.1
Desulfatirhabdium butyrativorans DSM 18734	Thermodesulfobacteriota	GCF_000429925.1
Desulfobulbus japonicus DSM 18378	Thermodesulfobacteriota	GCF_000429945.1
Tenacibaculum ovolyticum DSM 18103	Bacteroidota	GCF_000430545.1
Anaeroarcus burkinensis DSM 6283	Bacillota	GCF_000430605.1
Aneurinibacillus terranovensis DSM 18919	Bacillota	GCF_000430625.1
Tuberibacillus calidus DSM 17572	Bacillota	GCF_000430685.1
Azohydromonas australica DSM 1124	Pseudomonadota	GCF_000430725.1
Thermobrachium celere DSM 8682	Bacillota	GCF_000430995.1
Nosocomiicoccus massiliensis	Bacillota	GCF_000438455.1
Leminorella grimontii ATCC 33999 = DSM 5078	Pseudomonadota	GCF_000439085.1
Terrisporobacter glycolicus ATCC 14880 = DSM 1288	Bacillota	GCF_000439105.1
Tatumella saanichensis	Pseudomonadota	GCF_000439375.1
Salipiger mucosus DSM 16094	Pseudomonadota	GCF_000442255.1
Litoreibacter arenae DSM 19593	Pseudomonadota	GCF_000442275.2
Rubellimicrobium thermophilum DSM 16684	Pseudomonadota	GCF_000442315.1
Avibacterium paragallinarum JF4211	Pseudomonadota	GCF_000442905.1
Sporomusa ovata DSM 2662	Bacillota	GCF_000445445.1
haloarchaeon 3A1_DGR	Methanobacteriota	GCF_000447865.2
Paraclostridium bifermentans ATCC 638	Bacillota	GCF_000452245.2
Gorillibacterium massiliense	Bacillota	GCF_000455485.1
Siccibacter turicensis LMG 23730	Pseudomonadota	GCF_000463155.2
Streptococcus intermedius B196	Bacillota	GCF_000463355.1
Limimaricola cinnabarinus LL-001	Pseudomonadota	GCF_000466965.1
Leifsonia aquatica ATCC 14665	Actinomycetota	GCF_000469485.1
Chrysiogenes arsenatis DSM 11915	Chrysiogenota	GCF_000469585.1
Leisingera daeponensis DSM 23529	Pseudomonadota	GCF_000473145.1
Pseudophaeobacter arcticus DSM 23566	Pseudomonadota	GCF_000473205.1
Sedimentitalea nanhaiensis DSM 24252	Pseudomonadota	GCF_000473225.1
Adhaeribacter aquaticus DSM 16391	Bacteroidota	GCF_000473365.1
Rubidibacter lacunae KORDI 51-2	Cyanobacteriota	GCF_000473895.1
Coprobacter fastidiosus NSB1	Bacteroidota	GCF_000473955.1
Halarchaeum acidiphilum MH1-52-1	Methanobacteriota	GCF_000474235.1
Chitinivibrio alkaliphilus ACht1	Fibrobacterota	GCF_000474745.1
Lyngbya aestuarii BL J	Cyanobacteriota	GCF_000478195.2
Adlercreutzia equolifaciens DSM 19450	Actinomycetota	GCF_000478885.1
Cetobacterium somerae ATCC BAA-474	Fusobacteriota	GCF_000479045.1
Paucisalibacillus globulus DSM 18846	Bacillota	GCF_000482485.1
Derxia gummosa DSM 723	Pseudomonadota	GCF_000482785.1
Anaerobiospirillum succiniciproducens DSM 6400	Pseudomonadota	GCF_000482845.1
Mucispirillum schaedleri ASF457	Deferribacterota	GCF_000487995.1
Youngiibacter fragilis 232.1	Bacillota	GCF_000495435.3
Elizabethkingia anophelis NUHP1	Bacteroidota	GCF_000495935.2
Lutibaculum baratangense AMV1	Pseudomonadota	GCF_000496075.1
Methyloglobulus morosus KoM1	Pseudomonadota	GCF_000496735.2
Thalassobacillus devorans MSP14	Bacillota	GCF_000496835.1

Vagococcus lutrae LBD1	Bacillota	GCF_000498295.1
[Clostridium] dakarense	Bacillota	GCF_000499525.1
Actinospica robiniae DSM 44927	Actinomycetota	GCF_000504285.1
Pelistega indica	Pseudomonadota	GCF_000506865.1
Salinispira pacifica	Spirochaetota	GCF_000507245.1
Thermicanus aegyptius DSM 12793	Bacillota	GCF_000510645.1
Ignatzschineria larvae DSM 13226	Pseudomonadota	GCF_000510805.1
Sediminibacterium salmoneum NBRC 103935	Bacteroidota	GCF_000511175.1
Phaeobacter gallaeciensis DSM 26640	Pseudomonadota	GCF_000511385.1
[Clostridium] ultunense DSM 10521	Bacillota	GCF_000511955.1
Syntrophorhabdus aromaticivorans UI	Thermodesulfobacteriota	GCF_000512235.1
Dehalobacter restrictus DSM 9455	Bacillota	GCF_000512895.1
Rhodovibrio salinarum DSM 9154	Pseudomonadota	GCF_000515255.1
Jiangella gansuensis DSM 44835	Actinomycetota	GCF_000515395.1
Halonatronum saccharophilum DSM 13868	Bacillota	GCF_000517025.1
Alkaliflexus imshenetskii DSM 15055	Bacteroidota	GCF_000517065.1
Saccharicrinis fermentans DSM 9555 = JCM 21142	Bacteroidota	GCF_000517085.1
Sodalis praecaptivus	Pseudomonadota	GCF_000517425.1
Desulfurella acetivorans A63	Campylobacterota	GCF_000517565.1
Halostagnicola larsenii XH-48	Methanobacteriota	GCF_000517625.1
Entomoplasma lucivorax ATCC 49196	Mycoplasmatota	GCF_000518285.1
Olleya marilimosa CAM030	Bacteroidota	GCF_000518485.1
Phaseolibacter flectens ATCC 12775	Pseudomonadota	GCF_000518745.1
Desulfocurvus vexinensis DSM 17965	Thermodesulfobacteriota	GCF_000519125.1
Desulfonatronum lacustre DSM 10312	Thermodesulfobacteriota	GCF_000519265.1
Roseivivax marinus	Pseudomonadota	GCF_000520615.1
Aphanizomenon flos-aquae NIES-81	Cyanobacteriota	GCF_000521175.1
Advenella mimigardefordensis DPN7	Pseudomonadota	GCF_000521505.1
Tomitella biformata AHU 1821	Actinomycetota	GCF_000524475.1
Kutzneria albida DSM 43870	Actinomycetota	GCF_000525635.1
Mogibacterium timidum ATCC 33093	Bacillota	GCF_000525775.1
Brochothrix campestris FSL F6-1037	Bacillota	GCF_000525915.1
Aminiphilus circumscriptus DSM 16581	Synergistota	GCF_000526375.1
bacterium JKG1 Bacteria.	Chloroflexota	GCF_000526415.1
Caldicoprobacter oshimai DSM 21659	Bacillota	GCF_000526435.1
Atopobacter phocae ATCC BAA-285	Bacillota	GCF_000526675.1
Carnimonas nigrificans ATCC BAA-78	Pseudomonadota	GCF_000526695.1
Haloglycomyces albus DSM 45210	Actinomycetota	GCF_000527155.1
Salinivibrio costicola subsp. costicola ATCC 33508 = LMG 11651	Pseudomonadota	GCF_000565345.1
Skermanella stibiiresistens SB22	Pseudomonadota	GCF_000576635.1
[Scytonema hofmanni] UTEX 2349	Cyanobacteriota	GCF_000582685.1
Nitrincola nitratireducens	Pseudomonadota	GCF_000585235.1
Cloacibacillus evryensis DSM 19522	Synergistota	GCF_000585335.1
Cryptosporangium arvum DSM 44712	Actinomycetota	GCF_000585375.1
Aquamicrobium defluvii	Pseudomonadota	GCF_000585625.1
Roseibacterium elongatum DSM 19469	Pseudomonadota	GCF_000590925.1
Cobetia crustatorum	Pseudomonadota	GCF_000591415.1
Peptoclostridium acidaminophilum DSM 3953	Bacillota	GCF_000597865.1
Gilliamella apicola	Pseudomonadota	GCF_000599985.1
Snodgrassella alvi wkB2	Pseudomonadota	GCF_000600005.1
Fervidicella metallireducens AeB	Bacillota	GCF_000601455.1
Chondromyces apiculatus DSM 436	Myxococcota	GCF_000601485.1

Actinotalea ferrariae CF5-4	Actinomycetota	GCF_000603945.1
Trueperella pyogenes	Actinomycetota	GCF_000612055.1
Castellaniella defragrans 65Phen	Pseudomonadota	GCF_000612685.1
Asaia astilbis JCM 15831	Pseudomonadota	GCF_000613845.1
Sneathiella glossodoripedis JCM 23214	Pseudomonadota	GCF_000616095.1
Acidomonas methanolica NBRC 104435	Pseudomonadota	GCF_000617865.1
Methylobacillus glycogenes JCM 2850	Pseudomonadota	GCF_000617925.1
Alkanindiges illinoisensis DSM 15370	Pseudomonadota	GCF_000619845.1
Balneatrix alpica DSM 16621	Pseudomonadota	GCF_000619885.1
Zymobacter palmae DSM 10491	Pseudomonadota	GCF_000620025.1
Microvirgula aerodenitrificans DSM 15089	Pseudomonadota	GCF_000620105.1
Clostridiisalibacter paucivorans DSM 22131	Bacillota	GCF_000620125.1
Deefgea rivuli DSM 18356	Pseudomonadota	GCF_000620145.1
Anoxybacillus tepidamans PS2	Bacillota	GCF_000620165.1
Desulfitibacter alkalitolerans DSM 16504	Bacillota	GCF_000620305.1
Dyella japonica UNC79MFTsu3.2	Pseudomonadota	GCF_000620485.1
Desulfovermiculus halophilus DSM 18834	Thermodesulfobacteriota	GCF_000620765.1
Brackiella oedipodis DSM 13743	Pseudomonadota	GCF_000621025.1
Maribacter antarcticus DSM 21422	Bacteroidota	GCF_000621125.1
Enterovibrio calviensis DSM 14347	Pseudomonadota	GCF_000621165.1
Franconibacter pulveris DSM 19144	Pseudomonadota	GCF_000621185.1
Crocinitomix catalasitica ATCC 23190	Bacteroidota	GCF_000621625.1
Prolixibacter bellariivorans ATCC BAA-1284	Bacteroidota	GCF_000621705.1
Sulfitobacter geojensis	Pseudomonadota	GCF_000622325.1
Draconibacterium orientale	Bacteroidota	GCF_000626635.1
Parageobacillus caldoxylosilyticus NBRC 107762	Bacillota	GCF_000632715.1
Ectothiorhodospira haloalkaliphila ATCC 51935	Pseudomonadota	GCF_000633935.1
Actibacterium mucosum KCTC 23349	Pseudomonadota	GCF_000647975.1
Citrobacter freundii CFNIH1	Pseudomonadota	GCF_000648515.1
Desulfonatronovibrio hydrogenovorans DSM 9292	Thermodesulfobacteriota	GCF_000686525.1
Thermoanaerobaculum aquaticum	Acidobacteriota	GCF_000687145.1
Acidocella facilis ATCC 35904	Pseudomonadota	GCF_000687875.1
Terasakiella pusilla DSM 6293	Pseudomonadota	GCF_000688235.1
Simplicispira psychrophila DSM 11588	Pseudomonadota	GCF_000688255.1
Afifella pfennigii DSM 17143	Pseudomonadota	GCF_000688515.1
Lonsdalea quercina subsp. quercina	Pseudomonadota	GCF_000688655.1
Sphaerotilus natans subsp. natans DSM 6575	Pseudomonadota	GCF_000689195.1
Caballeronia zhejiangensis	Pseudomonadota	GCF_000698575.1
Nitrososphaera viennensis EN76	Nitrososphaerota	GCF_000698785.1
Rhodoluna lacicola	Actinomycetota	GCF_000699505.1
Paraoerskovia marina DSM 21750	Actinomycetota	GCF_000701465.1
Bilophila wadsworthia ATCC 49260	Thermodesulfobacteriota	GCF_000701705.1
Proteiniclasticum ruminis DSM 24773	Bacillota	GCF_000701905.1
Kandleria vitulina DSM 20405	Bacillota	GCF_000702065.1
Sharpea azabuensis DSM 18934	Bacillota	GCF_000702165.1
Bryobacter aggregatus MPL3	Acidobacteriota	GCF_000702445.1
Thermogemmatispora carboxidivorans	Chloroflexota	GCF_000702505.1
Pseudobutyrivibrio ruminis HUN009	Bacillota	GCF_000703005.1
Virgibacillus alimentarius	Bacillota	GCF_000709085.1
Planktothrix agardhii NIVA-CYA 126/8	Cyanobacteriota	GCF_000710505.1
Endozoicomonas elysicola	Pseudomonadota	GCF_000710775.1
Methanomicrobium mobile BP	Methanobacteriota	GCF_000711215.1

Polycyclovorans algicola TG408	Pseudomonadota	GCF_000711245.1
Hydrogenovibrio marinus DSM 11271	Pseudomonadota	GCF_000711315.1
Andreprevotia chitinilytica DSM 18519	Pseudomonadota	GCF_000711875.1
Methermicoccus shengliensis DSM 18856	Methanobacteriota	GCF_000711905.1
Synergistes jonesii	Synergistota	GCF_000712295.1
Thioclava indica	Pseudomonadota	GCF_000714545.1
Anditalea andensis	Bacteroidota	GCF_000714815.1
Tumebacillus flagellatus	Bacillota	GCF_000714935.1
Dactylosporangium aurantiacum	Actinomycetota	GCF_000716715.1
Streptomyces atroolivaceus	Actinomycetota	GCF_000717025.1
Catenuloplanes japonicus	Actinomycetota	GCF_000717135.1
Actinoalloteichus cyanogriseus	Actinomycetota	GCF_000718005.1
Mycetocola saprophilus	Actinomycetota	GCF_000718085.1
Spirillospora albida	Actinomycetota	GCF_000718255.1
Oerskovia turbata	Actinomycetota	GCF_000718325.1
Lentzea albidocapillata	Actinomycetota	GCF_000719115.1
Streptomyces albus subsp. albus	Actinomycetota	GCF_000719865.1
Prauserella rugosa	Actinomycetota	GCF_000719975.1
Marmoricola aequoreus	Actinomycetota	GCF_000720335.1
Phycicoccus jejuensis	Actinomycetota	GCF_000720925.1
Actinocatenispora sera	Actinomycetota	GCF_000720965.1
Pseudorhizobium pelagicum	Pseudomonadota	GCF_000722615.1
Janthinobacterium agaricidamnosum NBRC 102515 = DSM 9628	Pseudomonadota	GCF_000723165.1
Thaumarchaeota archaeon N4	Nitrososphaerota	GCF_000723185.1
Formosa agariphila KMM 3901	Bacteroidota	GCF_000723205.1
Hydrogenophaga intermedia	Pseudomonadota	GCF_000723405.1
Mucinivorans hirudinis	Bacteroidota	GCF_000723505.1
Fimbriimonas ginsengisoli Gsoil 348	Armatimonadota	GCF_000724625.1
Lysinibacillus fusiformis	Bacillota	GCF_000724775.3
Terribacillus goriensis	Bacillota	GCF_000725365.1
Dermacoccus nishinomiyaensis	Actinomycetota	GCF_000725405.1
Palaeococcus pacificus DY20341	Methanobacteriota	GCF_000725425.1
Nitrospirillum amazonense CBAmc	Pseudomonadota	GCF_000730165.1
Neorhizobium galegae bv. orientalis str. HAMBI 540	Pseudomonadota	GCF_000731315.1
Mangrovimonas yunxiaonensis	Bacteroidota	GCF_000733475.1
Algiphilus aromaticivorans DG1253	Pseudomonadota	GCF_000733765.1
Methylomarinum vadi	Pseudomonadota	GCF_000733935.1
Ferrovum myxofaciens	Pseudomonadota	GCF_000735045.1
Chryseobacterium halperniae	Bacteroidota	GCF_000735105.1
Kluyvera ascorbata ATCC 33433	Pseudomonadota	GCF_000735365.1
Yokenella regensburgei ATCC 49455	Pseudomonadota	GCF_000735455.1
Schleiferia thermophila str. Yellowstone	Bacteroidota	GCF_000736515.1
Hyalangium minutum	Myxococcota	GCF_000737315.1
Sandaracinus amylolyticus	Myxococcota	GCF_000737325.1
Halolamina rubra	Methanobacteriota	GCF_000739555.1
Halobellus rufus	Methanobacteriota	GCF_000739575.1
Tepidicaulis marinus	Pseudomonadota	GCF_000739695.1
Paenirhodobacter enshiensis	Pseudomonadota	GCF_000740785.1
Haematobacter massiliensis	Pseudomonadota	GCF_000740795.1
Devosia riboflavina	Pseudomonadota	GCF_000743575.1
Basilea psittacipulmonis DSM 24701	Pseudomonadota	GCF_000743945.1
Zobellia uliginosa	Bacteroidota	GCF_000744555.1

Streptacidiphilus rugosus AM-16	Actinomycetota	GCF_000744655.1
Porticoccus hydrocarbonoclasticus MCTG13d	Pseudomonadota	GCF_000744735.1
Thermorudis peleae	Thermomicrobiota	GCF_000744775.1
Mesoaciditoga lauensis cd-1655R = DSM 25116	Thermotogota	GCF_000745455.1
Belnapia moabensis DSM 16746	Pseudomonadota	GCF_000745835.1
Xenophilus azovorans DSM 13620	Pseudomonadota	GCF_000745855.1
Stenoxybacter acetivorans DSM 19021	Pseudomonadota	GCF_000745895.1
Ferrimicrobium acidiphilum DSM 19497	Actinomycetota	GCF_000745905.1
Alysiella crassa DSM 2578	Pseudomonadota	GCF_000745955.1
Edaphobacter aggregans DSM 19364	Acidobacteriota	GCF_000745965.1
Altibacter lentus	Bacteroidota	GCF_000746845.1
Melissococcus plutonius S1	Bacillota	GCF_000747585.1
Criblamydia sequanensis CRIB-18	Chlamydiota	GCF_000750955.1
Anaerosalibacter massiliensis	Bacillota	GCF_000751555.1
Acidihalobacter prosperus	Pseudomonadota	GCF_000754095.2
Halapricum salinum	Methanobacteriota	GCF_000755225.1
Pluralibacter gergoviae	Pseudomonadota	GCF_000757785.1
Cedecea neteri	Pseudomonadota	GCF_000757825.1
Arsenophonus endosymbiont str. Hangzhou of Nilaparvata lugens	Pseudomonadota	GCF_000757905.1
Pseudoglutamicibacter albus DNF00011	Actinomycetota	GCF_000758985.1
Phaeodactylibacter xiamenensis	Bacteroidota	GCF_000759025.1
Lysinimicrobium mangrovi	Actinomycetota	GCF_000759715.1
Oleiagrimonas soli	Pseudomonadota	GCF_000761445.1
Thermoactinomyces daqus	Bacillota	GCF_000763315.1
Pseudohaliea rubra DSM 19751	Pseudomonadota	GCF_000764025.1
Cryobacterium roopkundense	Actinomycetota	GCF_000764165.1
Jejuia pallidilutea	Bacteroidota	GCF_000764775.1
Pandoraea pnomenusa	Pseudomonadota	GCF_000767615.3
Knoellia flava TL1	Actinomycetota	GCF_000768675.1
Vibrio tubiashii ATCC 19109	Pseudomonadota	GCF_000772105.1
Chelonobacter oris	Pseudomonadota	GCF_000772535.1
Neosynechococcus sphagnicola sy1	Cyanobacteriota	GCF_000775285.1
bacterium endosymbiont of Mortierella elongata FMR23-6	Pseudomonadota	GCF_000775775.1
Pimelobacter simplex	Actinomycetota	GCF_000785495.1
Flaviramulus ichthyoenteri Th78	Bacteroidota	GCF_000789235.1
Geoglobus acetivorans	Methanobacteriota	GCF_000789255.1
Lacinutrix jangbogonensis	Bacteroidota	GCF_000797445.1
Robinsoniella peoriensis	Bacillota	GCF_000797495.1
Parvimonas micra	Bacillota	GCF_000800295.1
endosymbiont of unidentified scaly snail isolate Monju	Pseudomonadota	GCF_000801295.1
Sinomonas humi	Actinomycetota	GCF_000802235.1
Tepidimonas taiwanensis	Pseudomonadota	GCF_000807215.1
Frischella perrara	Pseudomonadota	GCF_000807275.1
Flammeovirga pacifica	Bacteroidota	GCF_000807855.2
Halocynthiibacter arcticus	Pseudomonadota	GCF_000812665.2
Flavihumibacter solisilvae	Bacteroidota	GCF_000814475.1
Allofrancisella guangzhouensis	Pseudomonadota	GCF_000815225.1
Pseudomonas balearica DSM 6083	Pseudomonadota	GCF_000818015.1
Jeotgalibacillus malaysiensis	Bacillota	GCF_000818095.1
Celeribacter indicus	Pseudomonadota	GCF_000819565.1
Geoalkalibacter ferrihydriticus DSM 17813	Thermodesulfobacteriota	GCF_000820505.1
Pyrinomonas methylaliphatogenes	Acidobacteriota	GCF_000820845.2

Tessaracoccus massiliensis	Actinomycetota	GCF_000826065.2
Streptomonospora alba	Actinomycetota	GCF_000826685.1
Necropsobacter massiliensis	Pseudomonadota	GCF_000827595.2
Methyloceanibacter caenitepidi	Pseudomonadota	GCF_000828475.1
Thiolapillus brandeum	Pseudomonadota	GCF_000828615.1
Sulfuritalea hydrogenivorans sk43H	Pseudomonadota	GCF_000828635.1
Thioploca ingrica	Pseudomonadota	GCF_000828835.1
Beduini massiliensis	Bacillota	GCF_000829905.1
Sanguibacteroides justesenii	Bacteroidota	GCF_000832075.1
Jannaschia aquimarina	Pseudomonadota	GCF_000877395.1
Dethiosulfatarculus sandiegensis	Thermodesulfobacteriota	GCF_000931935.2
Agreia bicolorata	Actinomycetota	GCF_000938265.1
Gynuella sunshinyii YC6258	Pseudomonadota	GCF_000940805.1
Siansivirga zeaxanthinifaciens CC-SAMT-1	Bacteroidota	GCF_000941055.1
Risungbinella massiliensis	Bacillota	GCF_000942395.1
Tamlana nanhaiensis	Bacteroidota	GCF_000943555.1
Syntrophaceticus schinkii	Bacillota	GCF_000946815.1
Thalassomonas actiniarum	Pseudomonadota	GCF_000948975.1
Acidithrix ferrooxidans	Actinomycetota	GCF_000949295.1
Ruthenibacterium lactatiformans	Bacillota	GCF_000949455.1
Rouxiella chamberiensis	Pseudomonadota	GCF_000951135.1
Aliterella atlantica CENA595	Cyanobacteriota	GCF_000952155.1
Aequorivita vladivostokensis	Bacteroidota	GCF_000952855.1
Tatlockia micdadei	Pseudomonadota	GCF_000953635.1
Defluviitoga tunisiensis	Thermotogota	GCF_000953715.1
Mizugakiibacter sediminis	Pseudomonadota	GCF_000953855.2
Caldicellulosiruptor naganoensis NA10	Bacillota	GCF_000955735.1
Burkholderia ubonensis MSMB22	Pseudomonadota	GCF_000959245.1
Martelella endophytica	Pseudomonadota	GCF_000960975.1
Elstera litoralis	Pseudomonadota	GCF_000963705.1
Tanticharoenia sakaeratensis NBRC 103193	Pseudomonadota	GCF_000963885.1
Williamsia herbipolensis	Actinomycetota	GCF_000964005.1
Luteibacter yeojuensis	Pseudomonadota	GCF_000964085.1
Acidisphaera rubrifaciens HS-AP3	Pseudomonadota	GCF_000964365.1
Alcaligenes faecalis	Pseudomonadota	GCF_000967305.2
Oleispira antarctica RB-8	Pseudomonadota	GCF_000967895.1
Aquincola tertiaricarbonis	Pseudomonadota	GCF_000969605.1
Nitriliruptor alkaliphilus DSM 45188	Actinomycetota	GCF_000969705.1
Methanosarcina thermophila TM-1	Methanobacteriota	GCF_000969885.1
Domibacillus tundrae	Bacillota	GCF_000970675.1
Limnoraphis robusta CS-951	Cyanobacteriota	GCF_000972705.2
Sneathia amnii	Fusobacteriota	GCF_000973085.1
Blochmannia endosymbiont of Polyrhachis (Hedomyrma) turneri	Pseudomonadota	GCF_000973505.1
Geofilum rubicundum JCM 15548	Bacteroidota	GCF_000974365.1
Ferriphaselus amnicola	Pseudomonadota	GCF_000974685.2
Demequina salsinemoris	Actinomycetota	GCF_000974805.1
Catabacter hongkongensis	Bacillota	GCF_000981035.1
Allosalinactinospora lopnorensis	Actinomycetota	GCF_000981225.1
Cronobacter sakazakii	Pseudomonadota	GCF_000982825.1
Kerstersia gyiorum	Pseudomonadota	GCF_000988095.1
Lampropedia cohaerens	Pseudomonadota	GCF_001005215.1
Silvibacterium bohemicum	Acidobacteriota	GCF_001006305.1

Altererythrobacter atlanticus	Pseudomonadota	GCF_001008165.2
Photorhabdus temperata subsp. thracensis	Pseudomonadota	GCF_001010285.1
Halanaeroarchaeum sulfurireducens	Methanobacteriota	GCF_001011115.1
[Polyangium] brachysporum	Pseudomonadota	GCF_001017435.1
Kiritimatiella glycovorans	Kiritimatiellota	GCF_001017655.1
Pragia fontium	Pseudomonadota	GCF_001026985.1
Archangium gephyra	Myxococcota	GCF_001027285.1
Endomicrobium proavitum	Elusimicrobiota	GCF_001027545.1
Muribacter muris	Pseudomonadota	GCF_001038205.1
Anaerobacillus macyae	Bacillota	GCF_001039475.1
Parascardovia denticolens DSM 10105 = JCM 12538	Actinomycetota	GCF_001042675.1
Marinovum algicola DG 898	Pseudomonadota	GCF_001046955.1
Fructobacillus ficulneus	Bacillota	GCF_001047075.2
Brenneria goodwinii	Pseudomonadota	GCF_001049335.1
Nereida ignava	Pseudomonadota	GCF_001049735.1
Rubeoparvulum massiliense	Bacillota	GCF_001049895.1
Longilinea arvoryzae	Chloroflexota	GCF_001050235.1
Leptolinea tardivitalis	Chloroflexota	GCF_001050275.1
Dakarella massiliensis	Pseudomonadota	GCF_001182045.1
Desulfocarbo indianensis	Thermodesulfobacteriota	GCF_001184205.1
Wenzhouxiangella marina	Pseudomonadota	GCF_001187785.1
Luteipulveratus mongoliensis	Actinomycetota	GCF_001190945.1
Caedimonas varicaedens	Pseudomonadota	GCF_001192655.1
Flexilinea flocculi	Chloroflexota	GCF_001192795.1
Lentimicrobium saccharophilum	Bacteroidota	GCF_001192835.1
Pseudaestuariivita atlantica	Pseudomonadota	GCF_001205715.1
Phaeobacter italicus	Pseudomonadota	GCF_001258055.1
Anaeromassilibacillus senegalensis	Bacillota	GCF_001261775.1
Pseudobacteroides cellulosolvens ATCC 35603 = DSM 2933	Bacillota	GCF_001262605.1
Vulgatibacter incomptus	Myxococcota	GCF_001263175.1
Thermincola ferriacetica	Bacillota	GCF_001263415.1
Oblitimonas alkaliphila	Pseudomonadota	GCF_001267175.1
Limnohabitans planktonicus II-D5	Pseudomonadota	GCF_001270065.2
Sunxiuqinia dokdonensis	Bacteroidota	GCF_001270965.1
Viridibacillus arvi	Bacillota	GCF_001274945.1
Halorubrum tropicale	Methanobacteriota	GCF_001280455.1
Sellimonas intestinalis	Bacillota	GCF_001280875.1
bacterium 336/3 Bacteria.		GCF_001281695.1
Lascolabacillus massiliensis	Bacteroidota	GCF_001282625.1
Marinagarivorans algicola	Pseudomonadota	GCF_001292705.1
Lawsonella clevelandensis	Actinomycetota	GCF_001293125.1
Ideonella sakaiensis	Pseudomonadota	GCF_001293525.1
Ardenticatena maritima	Chloroflexota	GCF_001293545.1
Desulfatitalea tepidiphila	Thermodesulfobacteriota	GCF_001293685.1
Amantichitinum ursilacus	Pseudomonadota	GCF_001294205.1
Moellerella wisconsensis ATCC 35017	Pseudomonadota	GCF_001294465.1
Trabulsiella odontotermitis	Pseudomonadota	GCF_001297765.1
Bosea vaviloviae	Pseudomonadota	GCF_001298265.1
Neofamilia massiliensis	Bacillota	GCF_001299475.1
Kibdelosporangium phytohabitans	Actinomycetota	GCF_001302585.1
Prosthecomicrobium hirschii	Pseudomonadota	GCF_001305515.1
Levilinea saccharolytica	Chloroflexota	GCF_001306035.1

Bellilinea caldifistulae	Chloroflexota	GCF_001306055.1
Ornatilinea apprima	Chloroflexota	GCF_001306115.1
Herpetosiphon geysericola	Chloroflexota	GCF_001306135.1
Thermanaerothrix daxensis	Chloroflexota	GCF_001306145.1
Croceitalea dokdonensis DOKDO 023	Bacteroidota	GCF_001306415.1
Mycobacterium fortuitum	Actinomycetota	GCF_001307545.1
Aliiroseovarius crassostreae	Pseudomonadota	GCF_001307765.1
Rufibacter tibetensis	Bacteroidota	GCF_001310085.1
Algibacter alginicilyticus	Bacteroidota	GCF_001310225.1
Stenotrophomonas pictorum JCM 9942	Pseudomonadota	GCF_001310775.1
Marinifilum fragile JCM 15579	Bacteroidota	GCF_001310955.1
Desulfosarcina cetonica JCM 12296	Thermodesulfobacteriota	GCF_001311845.1
Calditerricola satsumensis JCM 14719	Bacillota	GCF_001311905.1
Methylogaea oryzae JCM 16910	Pseudomonadota	GCF_001312345.1
Stenotrophomonas acidaminiphila	Pseudomonadota	GCF_001314305.1
Methanogenium cariaci JCM 10550	Methanobacteriota	GCF_001315945.1
Aeropyrum camini SY1 = JCM 12091	Thermoproteota	GCF_001316065.1
Oxobacter pfennigii	Bacillota	GCF_001317355.1
Flaviflexus massiliensis	Actinomycetota	GCF_001375495.1
Numidum massiliense	Bacillota	GCF_001375555.1
Massilibacterium senegalense	Bacillota	GCF_001375675.1
Thiohalorhabdus denitrificans	Pseudomonadota	GCF_001399755.1
Blastochloris viridis	Pseudomonadota	GCF_001402875.1
Acidiplasma aeolicum	Thermoplasmatota	GCF_001402945.1
Desnuesiella massiliensis	Bacillota	GCF_001403615.1
Fusicatenibacter saccharivorans	Bacillota	GCF_001405555.1
Lagierella massiliensis	Bacillota	GCF_001407835.1
Burkholderia cepacia ATCC 25416	Pseudomonadota	GCF_001411495.1
Pyrodictium delaneyi	Thermoproteota	GCF_001412615.1
Flagellimonas eckloniae	Bacteroidota	GCF_001413955.1
Chelatococcus sambhunathii	Pseudomonadota	GCF_001418005.1
Gulbenkiania indica	Pseudomonadota	GCF_001418035.1
Apibacter mensalis	Bacteroidota	GCF_001418685.1
Pseudohongiella spirulinae	Pseudomonadota	GCF_001444425.1
Aquabacterium parvum	Pseudomonadota	GCF_001447195.1
Terracidiphilus gabretensis	Acidobacteriota	GCF_001449115.1
Intestinimonas butyriciproducens	Bacillota	GCF_001454945.1
Mastigocoleus testarum BC008	Cyanobacteriota	GCF_001456025.1
Actinobaculum massiliense	Actinomycetota	GCF_001457435.1
Achromobacter xylosoxidans	Pseudomonadota	GCF_001457475.1
Fenollaria timonensis	Bacillota	GCF_001457835.1
Shimia marina	Pseudomonadota	GCF_001458175.1
Thalassobacter stenotrophicus	Pseudomonadota	GCF_001458315.1
Thalassobius gelatinovorus	Pseudomonadota	GCF_001458355.1
Epibacterium multivorans	Pseudomonadota	GCF_001458415.1
Haloparvum sedimenti	Methanobacteriota	GCF_001462205.1
Planococcus rifietoensis	Bacillota	GCF_001465795.2
Rathayibacter toxicus	Actinomycetota	GCF_001465855.1
Defluviitalea phaphyphila	Bacillota	GCF_001466305.1
Lacimicrobium alkaliphilum	Pseudomonadota	GCF_001466725.1
Haloprofundus marisrubri	Methanobacteriota	GCF_001469955.1
Pseudacidovorax intermedius	Pseudomonadota	GCF_001476815.1

Solirubrum puertoriconensis	Bacteroidota	GCF_001483135.1
Roseateles depolymerans	Pseudomonadota	GCF_001483865.1
Pannonibacter phragmitetus	Pseudomonadota	GCF_001484065.1
Bittarella massiliensis	Bacillota	GCF_001486165.1
Candidatus Protochlamydia naegleriophila	Chlamydiota	GCF_001499655.1
Pseudoruegeria marinistellae	Pseudomonadota	GCF_001509585.1
Aquitalea pelogenes	Pseudomonadota	GCF_001515305.1
Piscicoccus intestinalis NBRC 104926	Actinomycetota	GCF_001515525.1
Roseibaca calidilacus	Pseudomonadota	GCF_001517585.1
Caviibacter abscessus	Fusobacteriota	GCF_001517835.1
Oceanivirga salmonicida	Fusobacteriota	GCF_001517915.1
Microtetraspora malaysiensis	Actinomycetota	GCF_001528665.1
Microterricola viridarii	Actinomycetota	GCF_001542775.1
Aerococcus christensenii	Bacillota	GCF_001543105.1
Lutibacter profundi	Bacteroidota	GCF_001543325.1
Limnochorda pilosa	Bacillota	GCF_001544015.1
Thermosulfidibacter takaii ABI70S6	Thermosulfidibacterota	GCF_001547735.1
Caldimicrobium thiodismutans	Thermodesulfobacteriota	GCF_001548275.1
Kribbia dieselivorans NBRC 106261	Actinomycetota	GCF_001552435.1
Millisia brevis NBRC 105863	Actinomycetota	GCF_001552615.1
Skermania piniformis NBRC 15059	Actinomycetota	GCF_001552855.1
Xylophilus ampelinus	Pseudomonadota	GCF_001556675.1
Paramesorhizobium deserti	Pseudomonadota	GCF_001558695.1
methanogenic archaeon ISO4-H5	Thermoplasmatota	GCF_001560915.1
Tepidibacillus decaturensis	Bacillota	GCF_001561915.1
Pseudovibrio hongkongensis	Pseudomonadota	GCF_001561995.1
Thermotalea metallivorans	Bacillota	GCF_001562415.1
Fervidicola ferrireducens	Bacillota	GCF_001562425.1
Faecalibaculum rodentium	Bacillota	GCF_001564455.1
Herbidospora sakaeratensis	Actinomycetota	GCF_001570525.1
Herbiconiux solani NBRC 106740	Actinomycetota	GCF_001571005.1
Steroidobacter denitrificans	Pseudomonadota	GCF_001579945.1
Cephaloticoccus primus	Verrucomicrobiota	GCF_001580015.1
Ventosimonas gracilis	Pseudomonadota	GCF_001580025.1
Collimonas pratensis	Pseudomonadota	GCF_001584185.1
endosymbiont 'TC1' of Trimyema compressum	Bacillota	GCF_001584725.1
Obesumbacterium proteus	Pseudomonadota	GCF_001586165.1
Solibacillus silvestris	Bacillota	GCF_001586195.1
Zhongshania aliphaticivorans	Pseudomonadota	GCF_001586255.1
Bergeriella denitrificans NBRC 102155	Pseudomonadota	GCF_001592185.1
Roseivirga echinicomitans	Bacteroidota	GCF_001592935.1
Aggregatibacter actinomycetemcomitans	Pseudomonadota	GCF_001594265.1
Sulfurovum riftiae	Campylobacterota	GCF_001595645.1
Neptuniibacter marinus	Pseudomonadota	GCF_001597725.1
Mitsuaria chitosanitabida NBRC 102408	Pseudomonadota	GCF_001598255.1
Neokomagataea thailandica NBRC 106555	Pseudomonadota	GCF_001598495.1
Thermovenabulum gondwanense	Bacillota	GCF_001601575.1
Aminobacter aminovorans	Pseudomonadota	GCF_001605015.1
Geobacillus thermoleovorans	Bacillota	GCF_001610955.1
Anaerosporomusa subterranea	Bacillota	GCF_001611555.1
Haematospirillum jordaniae	Pseudomonadota	GCF_001611975.1
Terrimicrobium sacchariphilum	Verrucomicrobiota	GCF_001613545.1

Halofilum ochraceum	Pseudomonadota	GCF_001614315.2
Rummeliibacillus stabekisii	Bacillota	GCF_001617605.1
Tardiphaga robiniae	Pseudomonadota	GCF_001618955.1
Crenobacter luteus	Pseudomonadota	GCF_001619695.1
Defluviimonas alba	Pseudomonadota	GCF_001620265.1
Aeribacillus pallidus	Bacillota	GCF_001629795.1
Wohlfahrtiimonas chitiniclastica	Pseudomonadota	GCF_001632485.1
Dokdonella koreensis DS-123	Pseudomonadota	GCF_001632775.1
Mycobacterium chelonae CCUG 47445	Actinomycetota	GCF_001632805.1
Rhodovulum sulfidophilum DSM 1374	Pseudomonadota	GCF_001633165.1
Leptolyngbya valderiana BDU 20041	Cyanobacteriota	GCF_001637395.1
Acidibacillus ferrooxidans	Bacillota	GCF_001642725.1
Mariniblastus fucicola	Planctomycetota	GCF_001642875.1
Roseimaritima ulvae	Planctomycetota	GCF_001642915.1
Rubripirellula obstinata	Planctomycetota	GCF_001642955.1
Domibacillus aminovorans	Bacillota	GCF_001643245.1
Denitrobacterium detoxificans	Actinomycetota	GCF_001643775.1
Halotalea alkalilenta	Pseudomonadota	GCF_001648175.1
Thermosulfurimonas dismutans	Thermodesulfobacteriota	GCF_001652585.1
Christensenella minuta	Bacillota	GCF_001652705.1
Planomonospora sphaerica	Actinomycetota	GCF_001653075.1
Hydrogenibacillus schlegelii	Bacillota	GCF_001653195.1
Dokdonia donghaensis DSW-1	Bacteroidota	GCF_001653755.1
Buttiauxella ferragutiae ATCC 51602	Pseudomonadota	GCF_001654915.1
Mangrovibacter phragmitis	Pseudomonadota	GCF_001655675.1
Enteractinococcus helveticum	Actinomycetota	GCF_001657475.1
Labilibacter marinus	Bacteroidota	GCF_001659685.2
Croceicoccus marinus	Pseudomonadota	GCF_001661675.2
Cylindrospermopsis raciborskii CS-505	Cyanobacteriota	GCF_001676585.1
Woeseia oceani	Pseudomonadota	GCF_001677435.1
Dermabacter vaginalis	Actinomycetota	GCF_001678905.1
Salimicrobium jeotgali	Bacillota	GCF_001685435.2
Dissulfuribacter thermophilus	Thermodesulfobacteriota	GCF_001687335.1
Pararhizobium polonicum	Pseudomonadota	GCF_001687365.1
Flavonifractor plautii	Bacillota	GCF_001688625.2
Planobispora rosea	Actinomycetota	GCF_001696485.1
Wenyingzhuangia fucanilytica	Bacteroidota	GCF_001697185.1
Caryophanon tenue	Bacillota	GCF_001700315.1
Acidiferrobacter thiooxydans	Pseudomonadota	GCF_001705075.1
Methyloligella halotolerans	Pseudomonadota	GCF_001708935.1
Pantoea agglomerans	Pseudomonadota	GCF_001709315.1
Terasakiispira papahanaumokuakeensis	Pseudomonadota	GCF_001709345.1
Rhodohalobacter halophilus	Balneolota	GCF_001715195.1
Eisenbergiella tayi	Bacillota	GCF_001717135.1
Methylobrevis pamukkalensis	Pseudomonadota	GCF_001720135.1
Cellulosimicrobium cellulans	Actinomycetota	GCF_001722485.1
Desulfuribacillus alkaliarsenatis	Bacillota	GCF_001730225.1
Vulcanibacillus modesticaldus	Bacillota	GCF_001730235.1
Caloranaerobacter ferrireducens	Bacillota	GCF_001730685.1
Magnetovibrio blakemorei	Pseudomonadota	GCF_001746755.1
Lacunisphaera limnophila	Verrucomicrobiota	GCF_001746835.1
Fabibacter misakiensis	Bacteroidota	GCF_001747105.1

Desulfoplanes formicivorans	Thermodesulfobacteriota	GCF_001748225.1
Geosporobacter ferrireducens	Bacillota	GCF_001750685.1
Moorea producens PAL-8-15-08-1	Cyanobacteriota	GCF_001767235.1
Pediococcus acidilactici	Bacillota	GCF_001767275.1
Kozakia baliensis	Pseudomonadota	GCF_001787335.1
Thalassotalea crassostreae	Pseudomonadota	GCF_001831495.1
Gloeomargarita lithophora Alchichica-D10	Cyanobacteriota	GCF_001870225.1
Oceanisphaera psychrotolerans	Pseudomonadota	GCF_001870485.1
Rhodobaca barguzinensis	Pseudomonadota	GCF_001870665.2
Roseinatronobacter thiooxidans	Pseudomonadota	GCF_001870675.1
Arsenicibacter rosenii	Bacteroidota	GCF_001870735.1
Pseudodesulfovibrio hydrargyri	Thermodesulfobacteriota	GCF_001874525.1
Arcobacter lekithochrous	Campylobacterota	GCF_001878855.1
Nioella sediminis	Pseudomonadota	GCF_001879695.1
Couchioplanes caeruleus subsp. caeruleus	Actinomycetota	GCF_001884705.1
Natronohydrobacter thiooxidans	Pseudomonadota	GCF_001884735.1
Streptomyces cinnamoneus	Actinomycetota	GCF_001885705.1
Caldithrix abyssi DSM 13497	Calditrichota	GCF_001886815.1
Halodesulfurarchaeum formicicum	Methanobacteriota	GCF_001886955.1
Neomicrococcus aestuarii	Actinomycetota	GCF_001887245.1
Rappaport israeli	Pseudomonadota	GCF_001888055.1
Suttonella ornithocola	Pseudomonadota	GCF_001889065.1
Planktotalea frisia	Pseudomonadota	GCF_001890925.1
Phormidesmis priestleyi ULC007	Cyanobacteriota	GCF_001895925.1
Limnothrix rosea IAM M-220	Cyanobacteriota	GCF_001904615.1
Chroogloeocystis siderophila 5.2 s.c.1	Cyanobacteriota	GCF_001904655.1
Phormidium ambiguum IAM M-71	Cyanobacteriota	GCF_001904725.1
Actinophytocola xanthii	Actinomycetota	GCF_001921205.1
Acuticoccus yangtzensis	Pseudomonadota	GCF_001927245.1
Pajaroellobacter abortibovis	Myxococcota	GCF_001931505.1
Salinicola socius	Pseudomonadota	GCF_001937195.1
Paenisporosarcina indica	Bacillota	GCF_001939075.1
Tissierella creatinophila DSM 6911	Bacillota	GCF_001940565.1
Ileibacterium valens	Bacillota	GCF_001945525.1
Dubosiella newyorkensis	Bacillota	GCF_001945605.1
Paeniclostridium sordellii	Bacillota	GCF_001950115.1
Halioglobus pacificus	Pseudomonadota	GCF_001953075.1
Cnuella takakiae	Bacteroidota	GCF_001953305.1
Paludisphaera borealis	Planctomycetota	GCF_001956985.1
Tersicoccus phoenicis	Actinomycetota	GCF_001968835.1
Tateyamaria omphalii	Pseudomonadota	GCF_001969365.1
Thiobacimonas profunda	Pseudomonadota	GCF_001969385.1
Brevirhabdus pacifica	Pseudomonadota	GCF_001981245.1
Fuerstia marisgermanicae	Planctomycetota	GCF_001983935.1
Auricoccus indicus	Bacillota	GCF_001989575.1
Rodentibacter heylii	Pseudomonadota	GCF_001998905.1
Caenibacillus caldisaponilyticus	Bacillota	GCF_002003465.1
Novibacillus thermophilus	Bacillota	GCF_002005165.1
Paenalcaligenes hominis	Pseudomonadota	GCF_002005365.1
Jeotgalibaca dankookensis	Bacillota	GCF_002005405.1
Acetobacter aceti	Pseudomonadota	GCF_002005445.1
Neoasaia chiangmaiensis	Pseudomonadota	GCF_002005465.1

[Haemophilus] felis	Pseudomonadota	GCF_002015125.1
Plantibacter flavus	Actinomycetota	GCF_002024505.1
Thiomonas intermedia	Pseudomonadota	GCF_002028405.1
Methyloprofundus sedimenti	Pseudomonadota	GCF_002072955.1
Halomicronema hongdechloris C2206	Cyanobacteriota	GCF_002075285.3
Metallibacterium scheffleri	Pseudomonadota	GCF_002077135.1
Parasaccharibacter apium	Pseudomonadota	GCF_002079945.1
Oceanococcus atlanticus	Pseudomonadota	GCF_002088235.1
Pseudomonas nitroreducens NBRC 12694	Pseudomonadota	GCF_002091755.1
Thermoactinospora rubra	Actinomycetota	GCF_002093975.1
Cnuibacter physcomitrellae	Actinomycetota	GCF_002096055.1
Mycolicibacter hiberniae	Actinomycetota	GCF_002101655.1
Magnetofaba australis IT-1	Pseudomonadota	GCF_002109495.1
unicellular cyanobacterium SU2	Cyanobacteriota	GCF_002110465.1
Marivita cryptomonadis	Pseudomonadota	GCF_002115725.1
Rhizobacter gummiphilus	Pseudomonadota	GCF_002116905.1
Oceanicoccus sagamiensis	Pseudomonadota	GCF_002117105.1
Pseudorhodoplanes sinuspersici	Pseudomonadota	GCF_002119765.1
Macrococcus canis	Bacillota	GCF_002119805.1
Thioflexothrix psekupsii	Pseudomonadota	GCF_002149925.1
Methanonatronarchaeum thermophilum	Methanobacteriota	GCF_002153915.1
Crossiella equi	Actinomycetota	GCF_002155995.1
Natronolimnobius aegyptiacus	Methanobacteriota	GCF_002156705.1
Thaumasiovibrio subtropicus	Pseudomonadota	GCF_002157755.1
Massiliomicrobiota timonensis	Bacillota	GCF_002160865.1
Oleiphilus messinensis	Pseudomonadota	GCF_002162375.1
Nostocales cyanobacterium HT-58-2	Cyanobacteriota	GCF_002163975.1
Neiella marina	Pseudomonadota	GCF_002165625.1
Planomicrobium flavidum	Bacillota	GCF_002167005.1
Fimbriiglobus ruber	Planctomycetota	GCF_002197845.1
Pseudomonas aestusnigri	Pseudomonadota	GCF_002197985.1
Acutalibacter muris	Bacillota	GCF_002201475.1
Muribaculum intestinale	Bacteroidota	GCF_002201515.1
Alkalitalea saponilacus	Bacteroidota	GCF_002201795.1
Pelomonas puraquae	Pseudomonadota	GCF_002205845.1
Calderihabitans maritimus	Bacillota	GCF_002207765.1
Candidimonas nitroreducens	Pseudomonadota	GCF_002209565.1
Marinibacterium profundimaris	Pseudomonadota	GCF_002210095.1
Noviherbaspirillum denitrificans	Pseudomonadota	GCF_002211445.1
Diaphorobacter polyhydroxybutyrativorans	Pseudomonadota	GCF_002214645.1
Granulosicoccus antarcticus IMCC3135	Pseudomonadota	GCF_002215215.1
Sphingorhabdus flavimaris	Pseudomonadota	GCF_002218195.1
Xanthomonas citri pv. vignicola	Pseudomonadota	GCF_002218245.1
Turicimonas muris	Pseudomonadota	GCF_002221595.1
Dehalobacterium formicoaceticum	Bacillota	GCF_002224645.1
Neglecta timonensis	Bacillota	GCF_900048895.1
Murdochiella massiliensis	Bacillota	GCF_900059565.1
Trichococcus palustris	Bacillota	GCF_900067125.1
Devriesea agamarum	Actinomycetota	GCF_900070355.1
Emergencia timonensis	Bacillota	GCF_900086585.1
Bariatricus massiliensis	Bacillota	GCF_900086725.1
Plesiomonas shigelloides	Pseudomonadota	GCF_900087055.1

Orrella dioscoreae	Pseudomonadota	GCF_900089455.2
Hydrotalea flava	Bacteroidota	GCF_900089565.1
Cuniculiplasma divulgatum	Thermoplasmatota	GCF_900090055.1
secondary endosymbiont of Trabutina mannipara	Pseudomonadota	GCF_900090215.1
Culturomica massiliensis	Bacteroidota	GCF_900091655.1
Raineyella antarctica	Actinomycetota	GCF_900092135.1
Proteiniphilum saccharofermentans	Bacteroidota	GCF_900095135.1
Petrimonas mucosa	Bacteroidota	GCF_900095795.1
Colibacter massiliensis	Bacillota	GCF_900095855.1
Williamwhitmania taraxaci	Bacteroidota	GCF_900096565.1
Pelagirhabdus alkalitolerans	Bacillota	GCF_900096905.1
Anaerobium acetethylicum	Bacillota	GCF_900096945.1
Microbacteriaceae bacterium	Actinomycetota	GCF_900098805.1
Alteribacillus persepolensis	Bacillota	GCF_900099605.1
Propionivibrio dicarboxylicus	Pseudomonadota	GCF_900099695.1
Alloactinosynnema album	Actinomycetota	GCF_900099755.1
Lutimaribacter saemankumensis	Pseudomonadota	GCF_900100005.1
Salipiger marinus	Pseudomonadota	GCF_900100085.1
Dolosicoccus paucivorans	Bacillota	GCF_900100125.1
Ancylobacter rudongensis	Pseudomonadota	GCF_900100155.1
Natribacillus halophilus	Bacillota	GCF_900100185.1
Halovenus aranensis	Methanobacteriota	GCF_900100385.1
Roseospirillum parvum	Pseudomonadota	GCF_900100455.1
Sinosporangium album	Actinomycetota	GCF_900100605.1
Limimonas halophila	Pseudomonadota	GCF_900100655.1
Catalinimonas alkaloidigena	Bacteroidota	GCF_900100765.1
Natronincola ferrireducens	Bacillota	GCF_900100845.1
Halopelagius longus	Methanobacteriota	GCF_900100875.1
Methylophilus rhizosphaerae	Pseudomonadota	GCF_900100975.1
Sediminibacillus albus	Bacillota	GCF_900101125.1
Desulfoluna spongiiphila	Thermodesulfobacteriota	GCF_900101345.1
Mameliella alba	Pseudomonadota	GCF_900101505.1
Marinilactibacillus psychrotolerans	Bacillota	GCF_900101525.1
Auraticoccus monumenti	Actinomycetota	GCF_900101785.1
Pricia antarctica	Bacteroidota	GCF_900101815.1
Aquimonas voraii	Pseudomonadota	GCF_900101825.1
Peptococcus niger	Bacillota	GCF_900101835.1
Rhodospira trueperi	Pseudomonadota	GCF_900101965.1
Ulvibacter litoralis	Bacteroidota	GCF_900102055.1
Fontibacillus panacisegetis	Bacillota	GCF_900102215.1
Halorientalis regularis	Methanobacteriota	GCF_900102305.1
Geotoga petraea	Thermotogota	GCF_900102615.1
Melghirimyces thermohalophilus	Bacillota	GCF_900102685.1
Epibacterium ulvae	Pseudomonadota	GCF_900102795.1
Thiohalomonas denitrificans	Pseudomonadota	GCF_900102855.1
Albidovulum xiamenense	Pseudomonadota	GCF_900102905.1
Acidaminobacter hydrogenoformans DSM 2784	Bacillota	GCF_900103005.1
Halarsenatibacter silvermanii	Bacillota	GCF_900103135.1
Kriegella aquimaris	Bacteroidota	GCF_900103215.1
Siphonobacter aquaeclarae	Bacteroidota	GCF_900103285.1
Allisonella histaminiformans	Bacillota	GCF_900103425.1
Haloarchaeobius iranensis	Methanobacteriota	GCF_900103505.1

Allokutzneria albata	Actinomycetota	GCF_900103775.1
Acetanaerobacterium elongatum	Bacillota	GCF_900103835.1
Tenuibacillus multivorans	Bacillota	GCF_900103915.1
Desulfonauticus submarinus	Thermodesulfobacteriota	GCF_900104045.1
Cloacibacterium normanense	Bacteroidota	GCF_900104195.1
Filomicrobium insigne	Pseudomonadota	GCF_900104305.1
Desulforhopalus singaporensis	Thermodesulfobacteriota	GCF_900104445.1
Dendrosporobacter quercicolus	Bacillota	GCF_900104455.1
Bauldia litoralis	Pseudomonadota	GCF_900104485.1
Phocea massiliensis	Bacillota	GCF_900104615.1
Traorella massiliensis	Bacillota	GCF_900104665.1
Friedmanniella luteola	Actinomycetota	GCF_900105065.1
Streptomyces melanosporofaciens	Actinomycetota	GCF_900105695.1
Haloactinobacterium album	Actinomycetota	GCF_900105765.1
Albimonas donghaensis	Pseudomonadota	GCF_900106695.1
Tepidimicrobium xylanilyticum	Bacillota	GCF_900106765.1
Marininema mesophilum	Bacillota	GCF_900106775.1
Prevotellamassilia timonensis	Bacteroidota	GCF_900106785.1
Aidingimonas halophila	Pseudomonadota	GCF_900106955.1
Hydrobacter penzbergensis	Bacteroidota	GCF_900106985.1
Roseicitreum antarcticum	Pseudomonadota	GCF_900107025.1
Citreimonas salinaria	Pseudomonadota	GCF_900107235.1
Lachnobacterium bovis DSM 14045	Bacillota	GCF_900107245.1
Lentibacter algarum	Pseudomonadota	GCF_900107355.1
Tindallia californiensis	Bacillota	GCF_900107405.1
Asanoa ishikariensis	Actinomycetota	GCF_900107455.1
Proteiniborus ethanoligenes	Bacillota	GCF_900107485.1
Rubrimonas cliftonensis	Pseudomonadota	GCF_900107585.1
Desulfuromusa kysingii	Thermodesulfobacteriota	GCF_900107645.1
Arachidicoccus rhizosphaerae	Bacteroidota	GCF_900107765.1
Alkalimonas amylolytica	Pseudomonadota	GCF_900107845.1
Thermoleophilum album	Actinomycetota	GCF_900108055.1
Halopenitus malekzadehii	Methanobacteriota	GCF_900108505.1
Pseudospirillum japonicum	Pseudomonadota	GCF_900108915.1
Cribrihabitans marinus	Pseudomonadota	GCF_900109035.1
Halohasta litchfieldiae	Methanobacteriota	GCF_900109065.1
Alkalibacterium gilvum	Bacillota	GCF_900109085.1
Xiangella phaseoli	Actinomycetota	GCF_900109115.1
Parapedobacter koreensis	Bacteroidota	GCF_900109365.1
Pacificibacter marinus	Pseudomonadota	GCF_900109555.1
Pseudosphingobacterium domesticum	Bacteroidota	GCF_900109575.1
Nitrosovibrio tenuis	Pseudomonadota	GCF_900109785.1
Hydrogenoanaerobacterium saccharovorans	Bacillota	GCF_900110045.1
Maribius pelagius	Pseudomonadota	GCF_900110115.1
Lihuaxuella thermophila	Bacillota	GCF_900110165.1
Salinihabitans flavidus	Pseudomonadota	GCF_900110425.1
Propionispora vibrioides	Bacillota	GCF_900110485.1
Aquisalimonas asiatica	Pseudomonadota	GCF_900110585.1
Lewinella agarilytica	Bacteroidota	GCF_900110645.1
Ignavigranum ruoffiae	Bacillota	GCF_900110675.1
Piscibacillus halophilus	Bacillota	GCF_900110685.1
Litorimicrobium taeanense	Pseudomonadota	GCF_900110775.1

Aliicoccus persicus	Bacillota	GCF_900110815.1
Hyunsoonleella jejuensis	Bacteroidota	GCF_900111025.1
Rosenbergiella nectarea	Pseudomonadota	GCF_900111105.1
Giesbergeria anulus	Pseudomonadota	GCF_900111115.1
Salisediminibacterium haloalkalitolerans	Bacillota	GCF_900111285.1
Salipaludibacillus aurantiacus	Bacillota	GCF_900111295.1
Isobaculum melis	Bacillota	GCF_900111355.1
Thorsellia anophelis DSM 18579	Pseudomonadota	GCF_900111395.1
Salinibacillus kushneri	Bacillota	GCF_900111405.1
Poseidonocella pacifica	Pseudomonadota	GCF_900111875.1
Acetitomaculum ruminis DSM 5522	Bacillota	GCF_900112085.1
Brevinema andersonii	Spirochaetota	GCF_900112165.1
Tropicimonas isoalkanivorans	Pseudomonadota	GCF_900112335.1
Thiohalospira halophila DSM 15071	Pseudomonadota	GCF_900112605.1
Succiniclasticum ruminis DSM 9236	Bacillota	GCF_900112895.1
Sunxiuqinia elliptica	Bacteroidota	GCF_900113005.1
Thermoflexibacter ruber	Bacteroidota	GCF_900113045.1
Fontimonas thermophila	Pseudomonadota	GCF_900113085.1
Planifilum fulgidum	Bacillota	GCF_900113175.1
Planctomicrobium piriforme	Planctomycetota	GCF_900113665.1
Pisciglobus halotolerans	Bacillota	GCF_900113675.1
Thermoflavimicrobium dichotomicum	Bacillota	GCF_900114055.1
Halomicrobium zhouii	Methanobacteriota	GCF_900114435.1
Rugamonas rubra	Pseudomonadota	GCF_900114705.1
Salibacterium qingdaonense	Bacillota	GCF_900114715.1
Thermodesulforhabdus norvegica	Thermodesulfobacteriota	GCF_900114975.1
Algoriella xinjiangensis	Bacteroidota	GCF_900115015.1
Izhakiella capsodis	Pseudomonadota	GCF_900115045.1
Formivibrio citricus	Pseudomonadota	GCF_900115065.1
Salegentibacter flavus	Bacteroidota	GCF_900115145.1
Cohaesibacter marisflavi	Pseudomonadota	GCF_900115225.1
Anaerocolumna aminovalerica	Bacillota	GCF_900115365.1
Halolactibacillus alkaliphilus	Bacillota	GCF_900115465.1
Yuhushiella deserti	Actinomycetota	GCF_900115565.1
Tranquillimonas alkanivorans	Pseudomonadota	GCF_900115595.1
Hydrogenimonas thermophila	Campylobacterota	GCF_900115615.1
Ruminobacter amylophilus	Pseudomonadota	GCF_900115655.1
Pseudarcicella hirudinis	Bacteroidota	GCF_900115665.1
Parafilimonas terrae	Bacteroidota	GCF_900115755.1
Desemzia incerta	Bacillota	GCF_900115825.1
Donghicola eburneus	Pseudomonadota	GCF_900115865.1
Yangia pacifica	Pseudomonadota	GCF_900116195.1
Zhouia amylolytica	Bacteroidota	GCF_900116365.1
Lishizhenia tianjinensis	Bacteroidota	GCF_900116425.1
Crenotalea thermophila	Bacteroidota	GCF_900116565.1
Pustulibacterium marinum	Bacteroidota	GCF_900116665.1
Sinomicrobium oceani	Bacteroidota	GCF_900119185.1
Ezakiella massiliensis	Bacillota	GCF_900120165.1
Ndongobacter massiliensis	Bacillota	GCF_900120375.1
Mediterranea massiliensis	Bacteroidota	GCF_900128475.1
Alkalibacter saccharofermentans DSM 14828	Bacillota	GCF_900128885.1
Sphingorhabdus marina DSM 22363	Pseudomonadota	GCF_900128895.1

Ferrithrix thermotolerans DSM 19514	Actinomycetota	GCF_900128965.1
Mariniphaga anaerophila	Bacteroidota	GCF_900129025.1
Atopostipes suicloacalis DSM 15692	Bacillota	GCF_900129085.1
Lactonifactor longoviformis DSM 17459	Bacillota	GCF_900129135.1
Flavisolibacter ginsengisoli DSM 18119	Bacteroidota	GCF_900129295.1
Desulfacinum infernum DSM 9756	Thermodesulfobacteriota	GCF_900129305.1
Aliifodinibius roseus	Balneolota	GCF_900129315.1
Seinonella peptonophila	Bacillota	GCF_900129355.1
Streptoalloteichus hindustanus	Actinomycetota	GCF_900129375.1
Jatrophihabitans endophyticus	Actinomycetota	GCF_900129455.1
Flagellimonas flava	Bacteroidota	GCF_900129665.1
Chryseolinea serpens	Bacteroidota	GCF_900129725.1
Halobaculum gomorrense	Methanobacteriota	GCF_900129775.1
Thermosyntropha lipolytica DSM 11003	Bacillota	GCF_900129805.1
Asaccharospora irregularis DSM 2635	Bacillota	GCF_900129815.1
Tepidibacter thalassicus DSM 15285	Bacillota	GCF_900129915.1
Anaerosphaera aminiphila DSM 21120	Bacillota	GCF_900129925.1
Desulfofustis glycolicus DSM 9705	Thermodesulfobacteriota	GCF_900130015.1
Sporanaerobacter acetigenes DSM 13106	Bacillota	GCF_900130025.1
Sporobacter termitidis DSM 10068	Bacillota	GCF_900130065.1
Nissabacter archeti	Pseudomonadota	GCF_900130115.1
Cruoricaptor ignavus	Bacteroidota	GCF_900141665.1
Sulfurivirga caldicuralii	Pseudomonadota	GCF_900141795.1
Anaerovibrio lipolyticus DSM 3074	Bacillota	GCF_900141865.1
Tangfeifania diversioriginum	Bacteroidota	GCF_900141875.1
Pseudopelagicola gijangensis	Pseudomonadota	GCF_900142085.1
Lutispora thermophila DSM 19022	Bacillota	GCF_900142105.1
Malonomonas rubra DSM 5091	Thermodesulfobacteriota	GCF_900142125.1
Hespellia stercorisuis DSM 15480	Bacillota	GCF_900142165.1
Reichenbachiella agariperforans	Bacteroidota	GCF_900142205.1
Hathewaya proteolytica DSM 3090	Bacillota	GCF_900142225.1
Paramaledivibacter caminithermalis DSM 15212	Bacillota	GCF_900142245.1
Anaerobranca californiensis DSM 14826	Bacillota	GCF_900142275.1
Chishuiella changwenlii	Bacteroidota	GCF_900142565.1
Anaerosporobacter mobilis DSM 15930	Bacillota	GCF_900142955.1
Caldanaerovirga acetigignens	Bacillota	GCF_900142995.1
Oceanicella actignis	Pseudomonadota	GCF_900143155.1
Libanicoccus massiliensis	Actinomycetota	GCF_900143685.1
Desulfopila aestuarii DSM 18488	Thermodesulfobacteriota	GCF_900143695.1
Pseudoxanthobacter soli DSM 19599	Pseudomonadota	GCF_900148505.1
Merdibacter massiliensis	Bacillota	GCF_900155395.1
Negativicoccus massiliensis	Bacillota	GCF_900155405.1
Mobilibacterium timonense	Bacillota	GCF_900155555.1
Intestinibacillus massiliensis	Bacillota	GCF_900155735.1
Ectothiorhodosinus mongolicus	Pseudomonadota	GCF_900156225.1
Edaphobacillus lindanitolerans	Bacillota	GCF_900156305.1
Microbispora rosea	Actinomycetota	GCF_900156315.1
Pontibaca methylaminivorans	Pseudomonadota	GCF_900156525.1
Insolitispirillum peregrinum	Pseudomonadota	GCF_900156605.1
Kroppenstedtia eburnea	Bacillota	GCF_900156615.1
Oleibacter marinus	Pseudomonadota	GCF_900156675.1
Filimonas lacunae	Bacteroidota	GCF_900156765.1

Crenothrix polyspora	Pseudomonadota	GCF_900163755.1
Succinivibrio dextrinosolvens DSM 3072	Pseudomonadota	GCF_900167015.1
Carboxydocella sporoproducens DSM 16521	Bacillota	GCF_900167165.1
Selenihalanaerobacter shriftii	Bacillota	GCF_900167185.1
Garciella nitratireducens DSM 15102	Bacillota	GCF_900167305.1
Pilibacter termitis	Bacillota	GCF_900167335.1
Anaerorhabdus furcosa	Bacillota	GCF_900167375.1
Globicatella sulfidifaciens DSM 15739	Bacillota	GCF_900167405.1
Marinactinospora thermotolerans DSM 45154	Actinomycetota	GCF_900167435.1
Maledivibacter halophilus	Bacillota	GCF_900167445.1
Enhydrobacter aerosaccus	Pseudomonadota	GCF_900167455.1
Krasilnikoviella flava	Actinomycetota	GCF_900167525.1
Prosthecobacter debontii	Verrucomicrobiota	GCF_900167535.1
Gemmiger formicilis	Bacillota	GCF_900167555.1
Okibacterium fritillariae	Actinomycetota	GCF_900167575.1
Soonwooa buanensis	Bacteroidota	GCF_900167905.1
Ohtaekwangia koreensis	Bacteroidota	GCF_900167975.1
Paraliobacillus ryukyuensis	Bacillota	GCF_900168775.1
Phoenicibacter massiliensis	Actinomycetota	GCF_900169485.1
Arabia massiliensis	Actinomycetota	GCF_900169505.1
Desulfamplus magnetovallimortis	Thermodesulfobacteriota	GCF_900170035.1
Pseudooctadecabacter jejudonensis	Pseudomonadota	GCF_900172275.1
Pelagicola litorisediminis	Pseudomonadota	GCF_900172295.1
Palleronia marisminoris	Pseudomonadota	GCF_900172315.1
Oceanibacterium hippocampi	Pseudomonadota	GCF_900172325.1
Roseisalinus antarcticus	Pseudomonadota	GCF_900172355.1
Aquimixticola soesokkakensis	Pseudomonadota	GCF_900172375.1
Thermanaeromonas toyohensis ToBE	Bacillota	GCF_900176005.1
Desulfonispora thiosulfatigenes DSM 11270	Bacillota	GCF_900176035.1
Nitratiruptor tergarcus DSM 16512	Campylobacterota	GCF_900176045.1
Sulfobacillus thermosulfidooxidans DSM 9293	Bacillota	GCF_900176145.1
Papillibacter cinnamivorans DSM 12816	Bacillota	GCF_900176335.1
Moheibacter sediminis	Bacteroidota	GCF_900176425.1
Aquiflexum balticum DSM 16537	Bacteroidota	GCF_900176595.1
Pseudogulbenkiania subflava DSM 22618	Pseudomonadota	GCF_900177275.1
Tistlia consotensis USBA 355	Pseudomonadota	GCF_900177295.1
Brevefilum fermentans	Chloroflexota	GCF_900184705.1
Rhodoblastus acidophilus	Pseudomonadota	GCF_900187365.1
Thermoflexus hugenholtzii JAD2	Chloroflexota	GCF_900187885.1
Methylobacillus rhizosphaerae	Pseudomonadota	GCF_900187985.1
Puniceibacterium sediminis	Pseudomonadota	GCF_900188035.1
Haloechinothrix alba	Actinomycetota	GCF_900188115.1
Anaerovirgula multivorans	Bacillota	GCF_900188145.1
Ekhidna lutea	Bacteroidota	GCF_900188325.1
Antarctobacter heliothermus	Pseudomonadota	GCF_900188425.1
Amphiplicatus metriothermophilus	Pseudomonadota	GCF_900199215.1
Pelotomaculum thermopropionicum SI	Bacillota	GCA_000010565.1
